# Author Correction: Chemically triggered drug release from an antibody-drug conjugate leads to potent antitumour activity in mice

**DOI:** 10.1038/s41467-019-08376-x

**Published:** 2019-01-16

**Authors:** Raffaella Rossin, Ron M. Versteegen, Jeremy Wu, Alisher Khasanov, Hans J. Wessels, Erik J. Steenbergen, Wolter ten Hoeve, Henk M. Janssen, Arthur H. A. M. van Onzen, Peter J. Hudson, Marc S. Robillard

**Affiliations:** 1 Tagworks Pharmaceuticals, Geert Grooteplein Zuid 10, 6525 GA Nijmegen, The Netherlands; 2 SyMO-Chem B.V., Den Dolech 2, 5612 AZ Eindhoven, The Netherlands; 3 Avipep Pty Ltd, 343 Royal Parade, Parkville, VIC 3052 Australia; 4 Levena Biopharma, 4955 Directors Place, Suite 300, San Diego, CA 92121 USA; 5 Radboud Proteomics Centre, Department of Laboratory Medicine, Radboud University Medical Center, P.O. Box 9101, 6500 HB Nijmegen, The Netherlands; 6 Department of Pathology, Radboud University Medical Center, P.O. Box 9101, 6500 HB Nijmegen, The Netherlands; 7 Syncom B.V., Kadijk 3, 9747 AT Groningen, The Netherlands

Correction to: *Nature Communications*; 10.1038/s41467-018-03880-y; published online 04 May 2018.

The original version of this Article omitted the following from the Acknowledgements: ‘This work was supported by the Office of the Assistant Secretary of Defense for Health Affairs, through the Breast Cancer Research Program under Award No. W81XWH-15-1-0692. Opinions, interpretations, conclusions and recommendations are those of the author and are not necessarily endorsed by the Department of Defense’. This error has now been corrected in the PDF and HTML versions of the Article.

