# Supplementary material for: Chemically triggered drug release from an antibody-drug conjugate leads to potent antitumour activity in mice
Source: Nat Commun. 2018 May 4;9:1484. doi: 10.1038/s41467-018-03880-y (PMC5935733; doi:10.1038/s41467-018-03880-y)
Supplement: Supplementary file 1 — Supplementary Information [file 41467_2018_3880_MOESM1_ESM.pdf]

Supplementary Information

Tetrazine-triggered drug release from an antibody−drug conjugate leads to potent antitumour activity in mice

Rossin et al.

Supplementary Figures

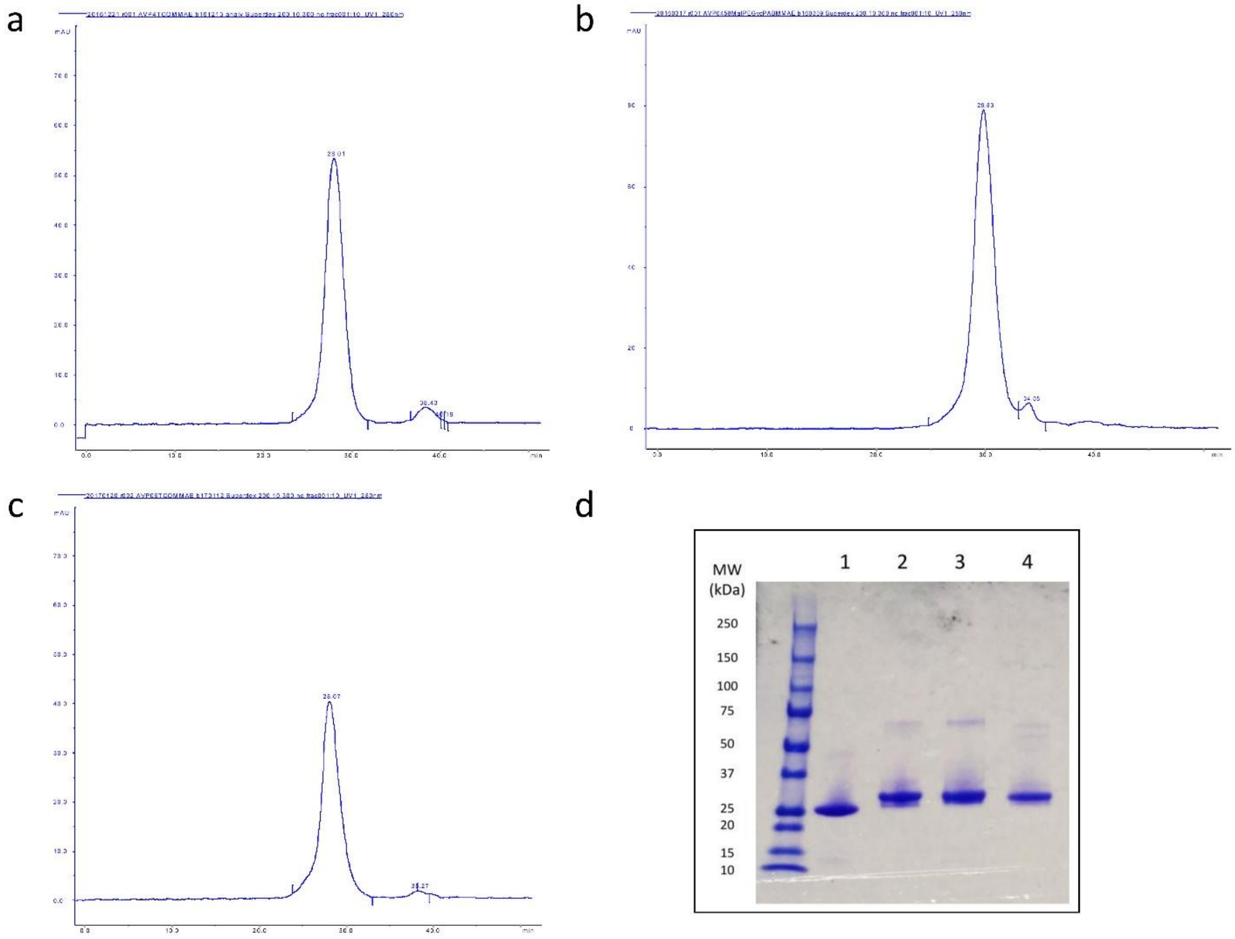

Supplementary Figure 1. ADC characterization. Size exclusion chromatography (SEC) analysis of purified (a) **tc-ADC**, (b) **vc-ADC** and (c) **nb-ADC**. (d) SDS-PAGE analysis of (1) AVP04-58, (2) **tc-ADC**, (3) **vc-ADC**, and (4) **nb-ADC**, showing the diabody monomers at 25-30 kDa.

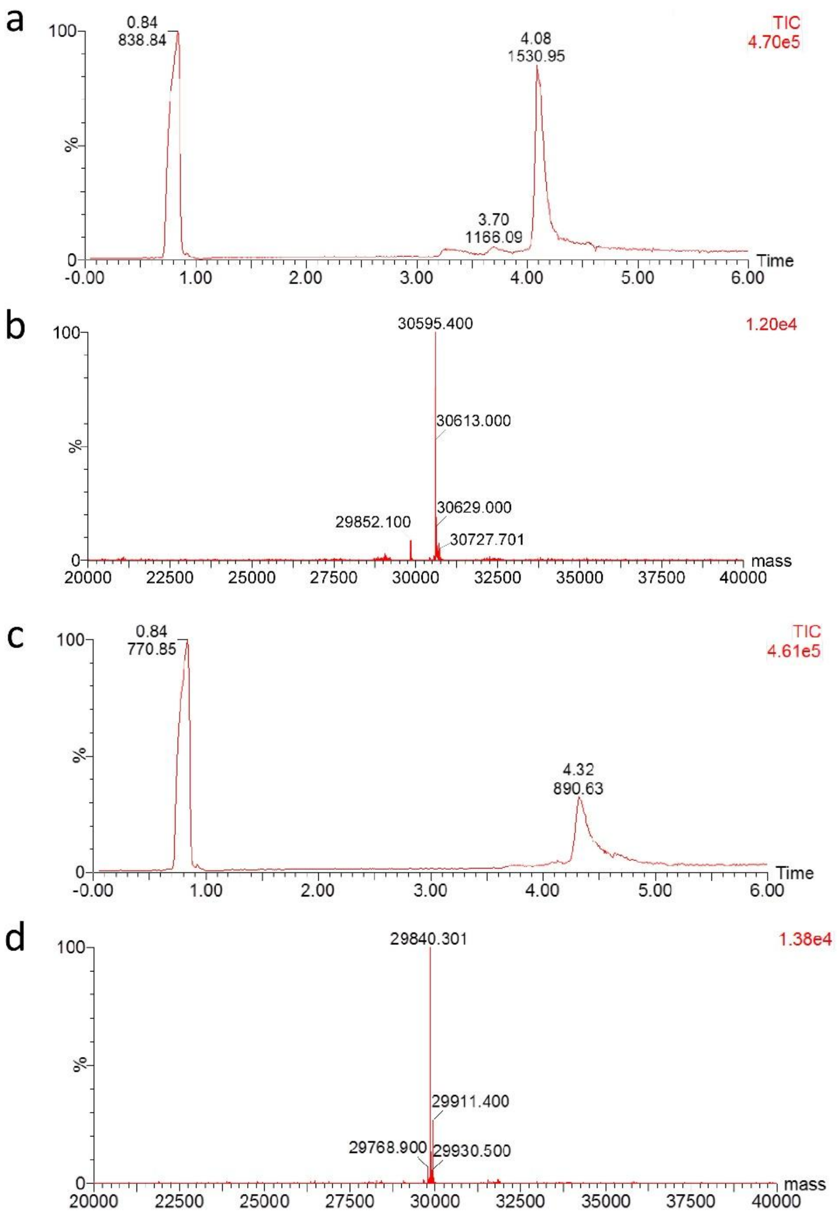

Supplementary Figure 2. HPLC-QTOF-MS analysis of tc-ADC and nb-ADC stock solutions. Diabody conjugate **tc-ADC** stored at 4°C in 5% DMSO/EDTA-PB: (a) HPLC chromatogram; (b) MS spectrum after deconvolution, showing mass of the diabody monomer with DAR of 2. Diabody conjugate **nb-ADC** storage at 4°C in 5% DMSO/EDTA-PB: (c) HPLC chromatogram; (d) MS spectrum after deconvolution, showing mass of the diabody monomer with DAR of 2. Spectra unchanged for at least 6 months.

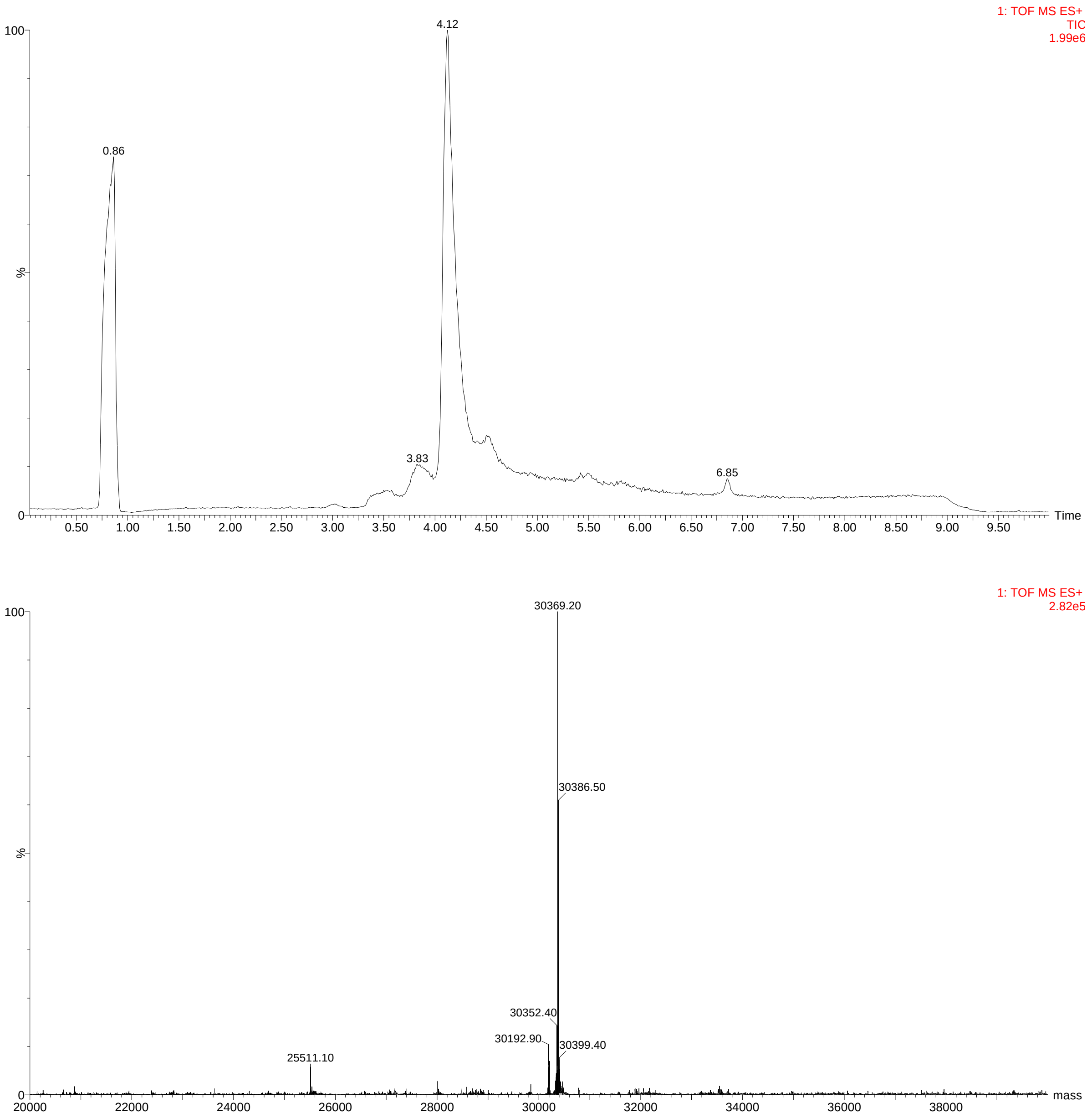

Supplementary Figure 3. HPLC-QTOF-MS analysis of vc-ADC stock solution. After storage for 18 months at 4°C in 5% DMSO/EDTA-PB; top: HPLC chromatogram; bottom: MS spectrum after deconvolution, showing mass of the diabody monomer with DAR of 2.

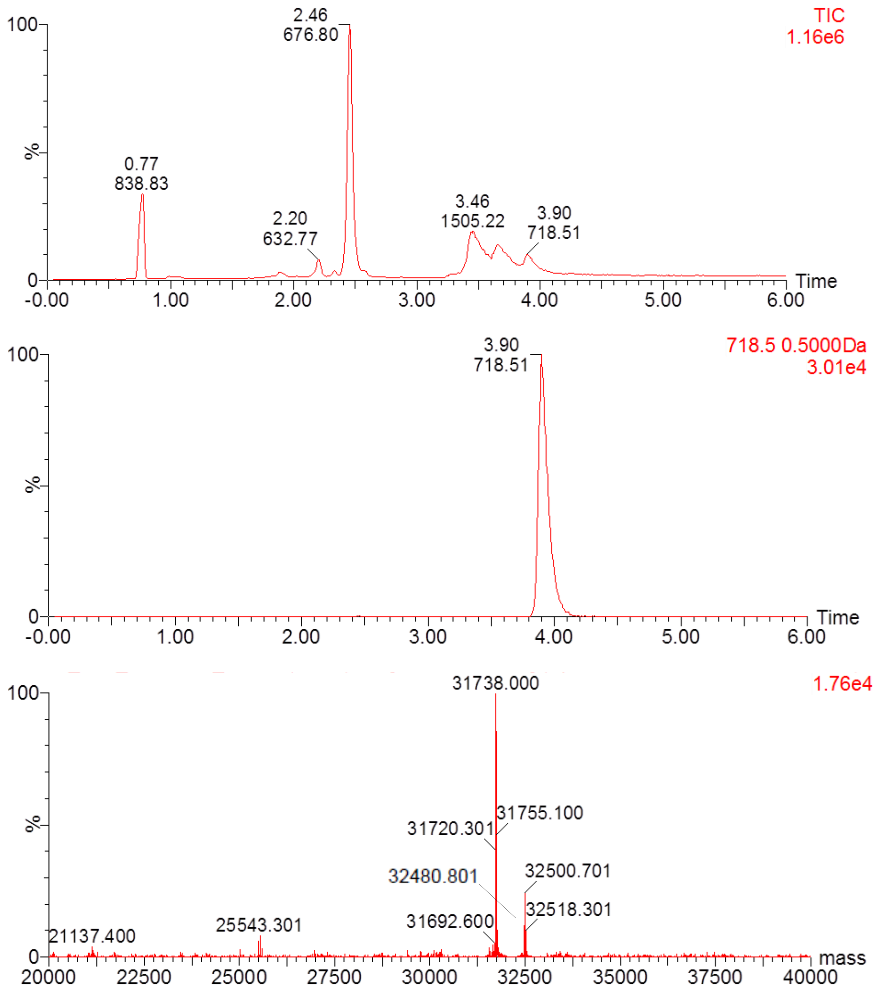

Supplementary Figure 4. HPLC-QTOF-MS analysis of tc-ADC activation mixture. Diabody conjugate **tc-ADC** with activator **3** in PBS; top: HPLC chromatogram (peak at 2.46 min is excess activator and at 3.90 min is free MMAE); middle: HPLC chromatogram filtered for *m/z*=718.51 Da (free MMAE); bottom: MS spectrum of the diabody conjugate after summation of the range from 3.2 – 4.2 min and subsequent deconvolution, showing fully reacted ADC with 2 × MMAE release (31720 Da) and a minor amount of fully reacted ADC with 1 × MMAE release (32481 Da).

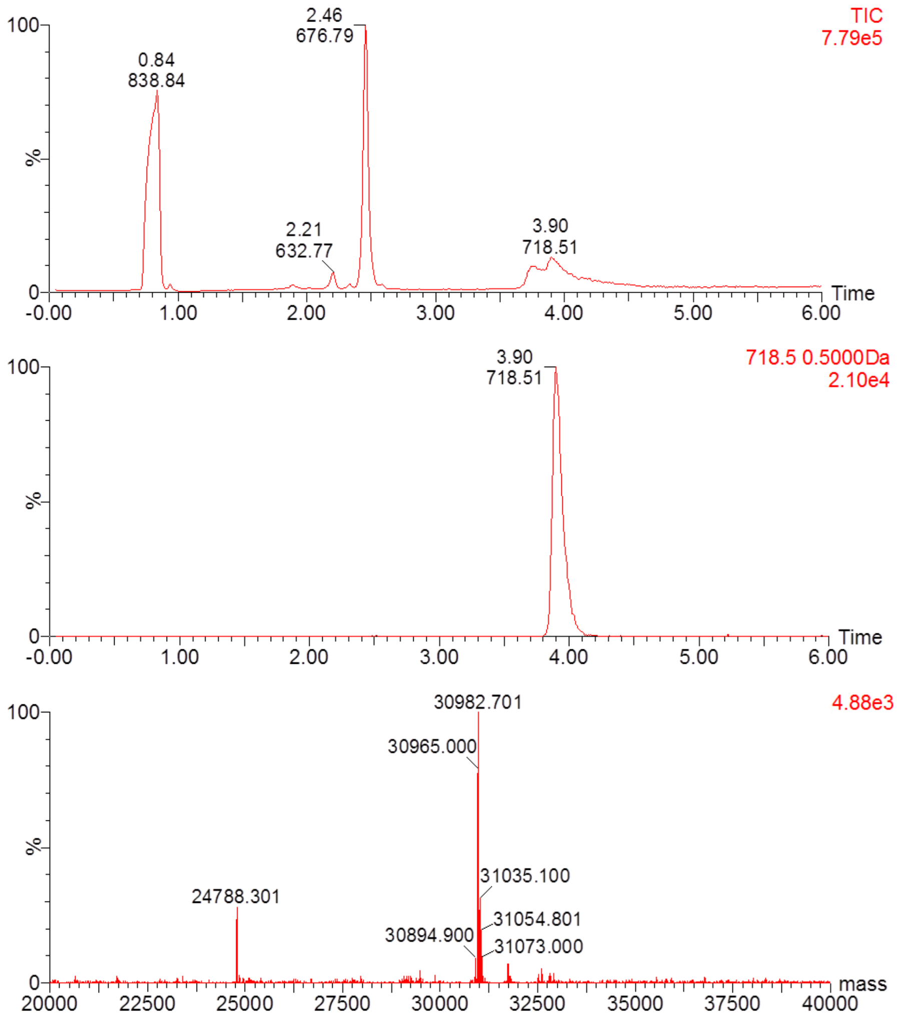

Supplementary Figure 5. HPLC-QTOF-MS analysis of nb-ADC activation mixture. Diabody conjugate **nb-ADC** with activator **3** in PBS; top: HPLC chromatogram (peak at 2.46 min is excess activator and at 3.90 min is free MMAE); middle: HPLC chromatogram filtered for *m/z*=718.51 Da (free MMAE); bottom: MS spectrum of the diabody conjugate after summation of the range from 3.5 – 4.5 minute, and subsequent deconvolution, showing fully reacted ADC with 2 × MMAE release (30965 Da).

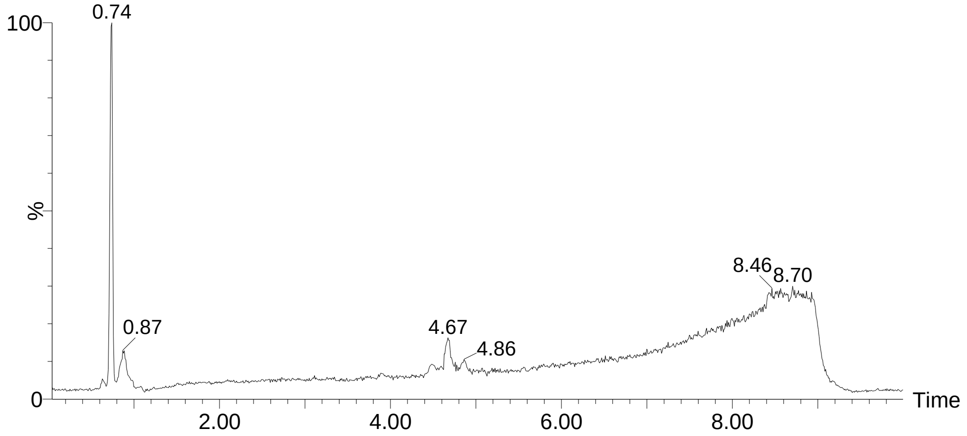

Supplementary Figure 6. Stability of diabody conjugate in mouse serum. LCMS analysis of deproteinized supernatant originating from a **tc-ADC** solution in 50% mouse serum after 24 h incubation at 37°C. The peak at 4.5-4.9 min corresponds to residual serum proteins. An aliquot of the stock **tc-ADC** solution (50 µL 2 µg µL^{-1} in 5% DMSO/EDTA-PB) was two-fold diluted with mouse serum and incubated at 37°C. After 1, 5 and 24 h incubation aliquots of the solution were taken and proteins were precipitated by adding two parts of ice-cold acetonitrile. After vortexing, 30 min standing at 4°C and centrifugation (13,000 rpm, 5 min), the supernatants were separated from the protein pellets, diluted with two parts of water and analysed by HPLC-QTOF-MS. In these conditions, no free MMAE (R_t = 3.82 min) or other degradation products were observed up to 24 h incubation. The limit of detection for MMAE in this assay is 1 × 10^{-4} µg µl^{-1}. As the concentration of total MMAE in the assay is 0.0472 µg µl^{-1} it follows that the percentage of bound MMAE at 24 h is >99.8%.

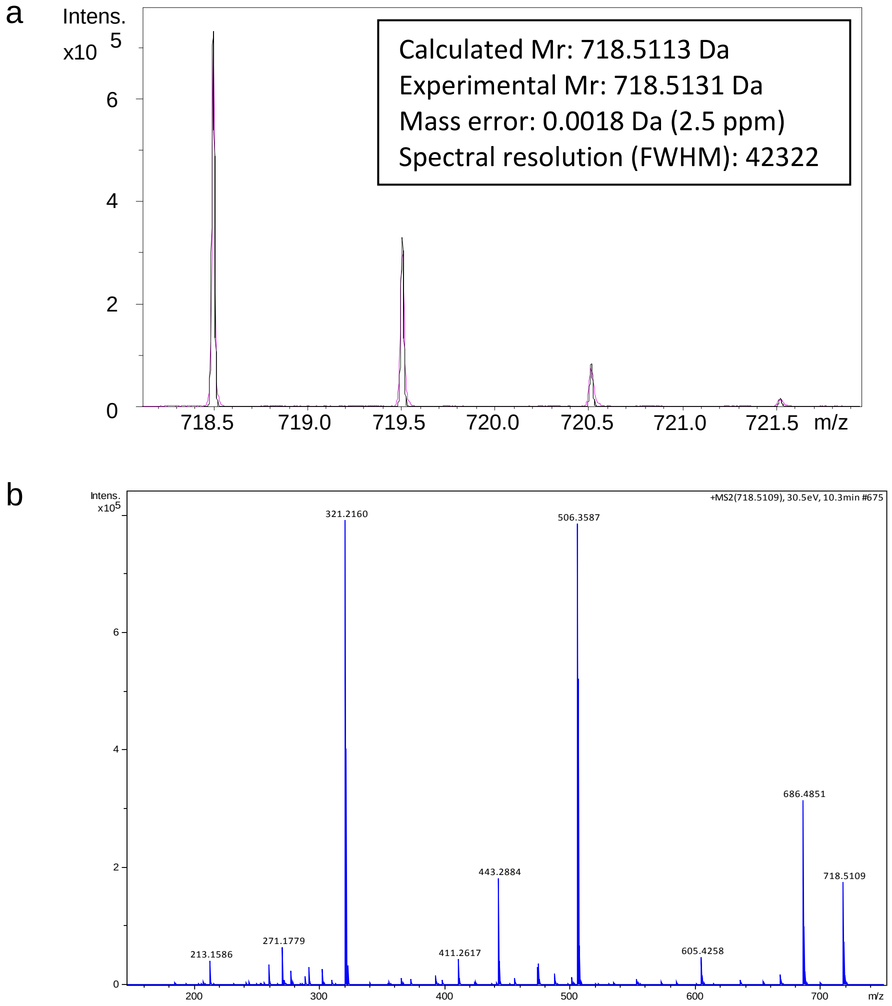

Supplementary Figure 7. HPLC-QTOF-MS analysis of ADC activation in serum. (a) MS analysis of MMAE released from **tc-ADC** in 50% mouse serum at 37°C (black: simulated spectrum; pink: measured spectrum); (b) MS-MS analysis of MMAE released from **tc-ADC** in 50% mouse serum at 37°C. MMAE release yields were determined by LC-MS using a nano-Advance ultra-high pressure nanoflow liquid chromatograph coupled online to an ultra-high resolution QTOF mass spectrometer (maXis 5G, Bruker Daltonics) *via* an axial desolvation vacuum assisted nanoflow electrospray ionization source (Captive Sprayer, Bruker Daltonics). Samples were injected onto a C_{18} RP trapping column (Acclaim PepMap RSLC C_{18}, Thermo Fisher Scientific: 75µm ID × 2 cm length, 3µm particles, 100Å pore size) at 7 µL min^{-1} using 2 sample loop volumes (40 µl) of 0.1% acetic acid. Analytes were separated by C_{18} RP chromatography (Acclaim PepMap RSLC C_{18}: 75µm ID × 15 cm length, 2 µm particles, 100Å pore size) at 45°C in 15 min using a linear gradient of 10-60% acetonitrile in 0.1% acetic acid at a flow rate of 500 nL min^{-1}. The mass spectrometer was operated in positive ion mode at 1 Hz spectral acquisition rate. Acquired data files were internally mass calibrated using sodium acetate clusters in DataAnalysis 4.2 software (Bruker Daltonics). MMAE was readily identified in LC-MS data by accurate monoisotopic mass measurement (*m/z* 718.5131 Da $[M+H]^{1+}$, mass error: 0.0015 Da / 2.5 ppm) and the simulated isotope distribution pattern of MMAE ($C_{39}H_{67}N_5O_7$). Quantification of MMAE was performed against external standards (MMAE concentration range in PBS/serum) in QuantAnalysis software (Bruker Daltonics) using integrated chromatographic peak areas from high-resolution extracted ion current chromatograms for the second isotope of MMAE (*m/z* 720.52 ± 0.02 Th) at retention time 10.4 ± 0.5 min.

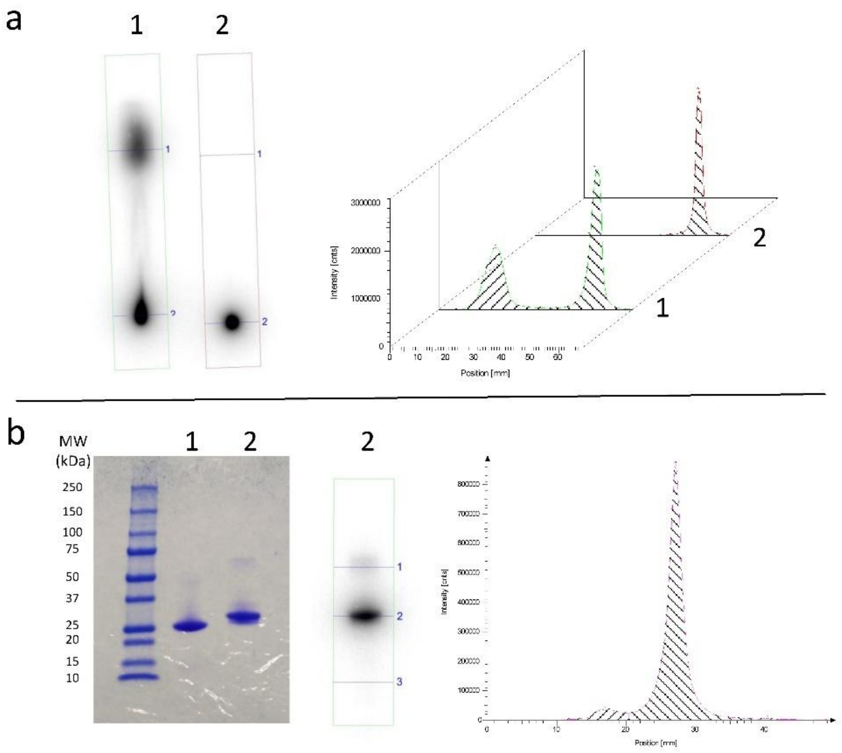

Supplementary Figure 8. Diabody conjugate tc-ADC radioiodination and analysis. (a) Radio-ITLC analysis of (1) crude and (2) purified ^{125}I-labelled **tc-ADC**; (b) SDS-PAGE analysis (protein stain and radiogram) of (1) AVP04-58 monomer and (2) ^{125}I-labelled **tc-ADC** monomer showing <5% aggregates. The diabody-based ADCs (100 µg) were labelled with iodine-125 (5-10 MBq) with the Bolton-Hunter reagent, as previously described[1]. The crude labelling mixtures were passed twice over Zeba desalting spin columns (7 kDa MW cut-off) equilibrated with PBS affording ^{125}I-labelled ADCs with greater than 95% radiochemical purity, as confirmed by radio-ITLC and SDS-PAGE (panels a-b, lane 2). For animal experiments the ^{125}I-labelled ADCs were combined with the respective non-radioactive compound to the desired molar activity (*vide infra*) and diluted with sterile saline prior to injection.

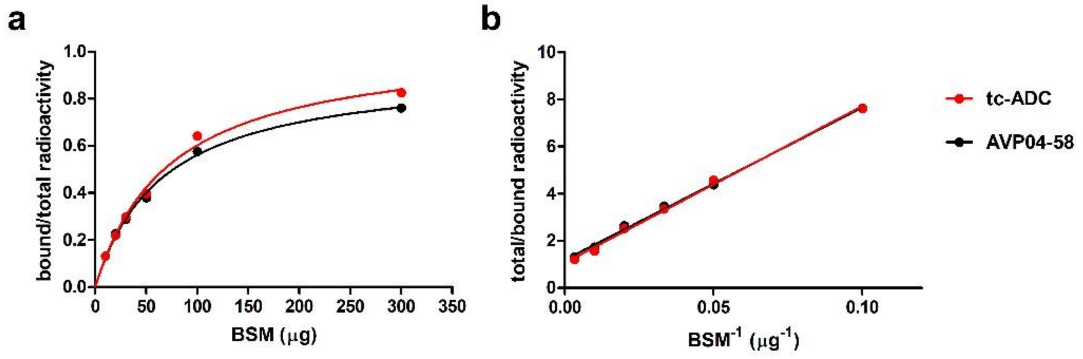

Supplementary Figure 9. Immunoreactivity assay. (a) Binding of ^{125}I-labelled **tc-ADC** or AVP04-58 (1 μg) to increasing amounts of bovine submaxillary mucin (BSM) as determined with size exclusion chromatography. (b) Plot of total-to-bound radioactivity ratio *vs.* the inverse BSM concentration showing a linear correlation. The immunoreactivity of **tc-ADC** was evaluated as described by Ngai and Reilly[2], with some modifications. The ^{125}I-labelled ADC (1 μg) was incubated with increasing amounts of bovine submaxillary mucin (BSM; 10-300 μg) in a 1% bovine serum albumin (BSA) solution in PBS containing 3% DMSO for 2 h at room temperature, followed by SEC (Akta system equipped with a Superdex 200 column eluted with PBS containing 1% DMSO at 0.5 mL min^{-1}). The amount of BSM-bound and total radioactivity in the eluate were measured by collecting 0.5 mL fractions followed by γ-counting. The results show saturatable binding of **tc-ADC** to BSM (a) and when plotted as total/bound radioactivity *vs.* the inverse of BSM concentration, as described by Lindmo *et al.*[3], a good linear relationship is found (b; R^2 = 0.996) with an extrapolated ordinate intercept (r) of 1.06. This value yields an immunoreactive fraction at infinite antigen (r^{-1}) of 94%. In comparison, the native AVP04-58 (containing 5% aggregates) shows 84% immunoreactivity (r = 1.195; R^2 = 0.998).

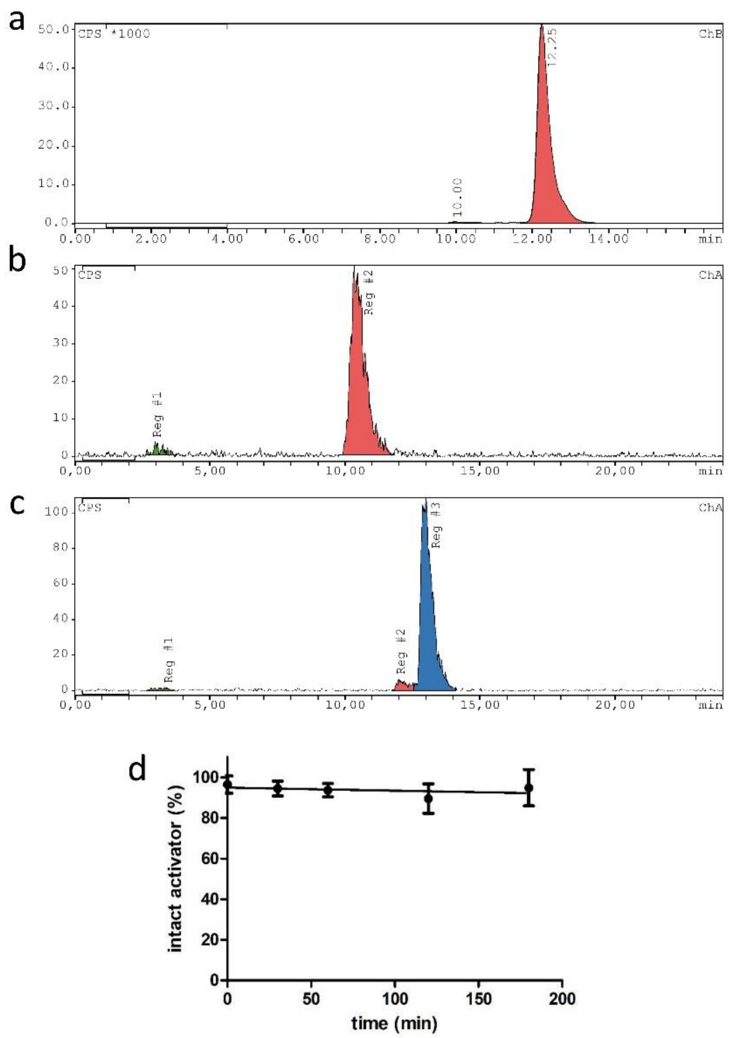

Supplementary Figure 10. Analysis of radiolabelled tetrazines. Radio-RP-HPLC analysis of (a) purified [^{18}F]F-**S24** (R_t = 12.3 min), (b) [^{177}Lu]Lu-**3** (R_t = 10.3 min), and (c) [^{177}Lu]Lu-**4** (R_t = 13.0 min) in PBS. (d) Radio-RP-HPLC analysis of the in vitro stability of [^{177}Lu]Lu-**3** in 50% mouse serum at 37°C. The data are the mean with s.d. (n=3).

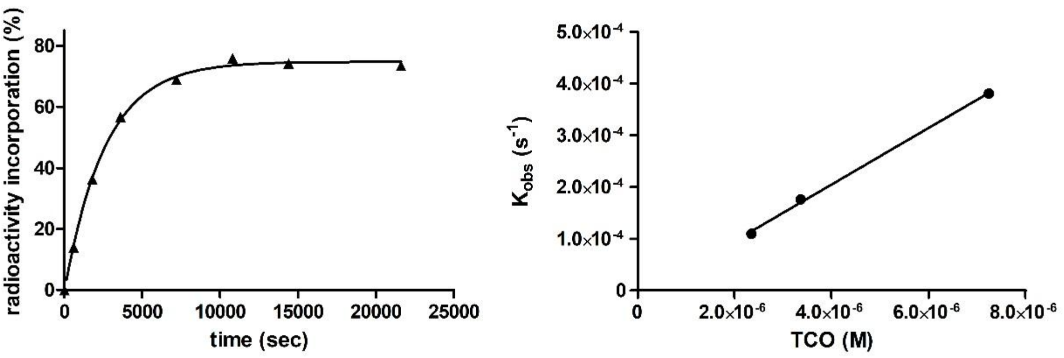

Supplementary Figure 11. Kinetics measurements. Reaction between **tc-ADC** and [^{177}Lu]Lu-**3** (<0.1 eq. with respect to TCO) in PBS at 37°C; (left) reaction of [^{177}Lu]Lu-**3** with **tc-ADC** (1.8 µM) in time, monitored by radio-TLC, and fitted to a first order exponential. The reaction kinetics between **tc-ADC** and activator **3** was determined as previously published[4] with minor modifications. Briefly, precursor **S4** was radiolabelled with no-carrier added lutetium-177 at 2.6 MBq nmol^{-1} molar activity. The obtained radioactive activator [^{177}Lu]Lu-**3** (0.2 µM) was then reacted with increasing concentrations of **tc-ADC** (0.6-1.8 µM) in PBS at 37°C. At various times between 10 min and 6 h, aliquots of the reaction mixture were taken and quenched with a large excess of a 3,6-bispyridine-tetrazine derivative[4]. After 20 min incubation at room temperature, these aliquots were analysed by RP-TLC and phosphor imager. A pseudo first order rate constant (K_{obs}) was determined from the fit. (Right) plot of K_{obs} vs. the TCO concentration, fitted using linear regression. Based on K_{obs}=[TCO](k_2), the second order rate constant is the slope of the line, and was found to be 54.7 ± 2.2 M^{-1} s^{-1}.

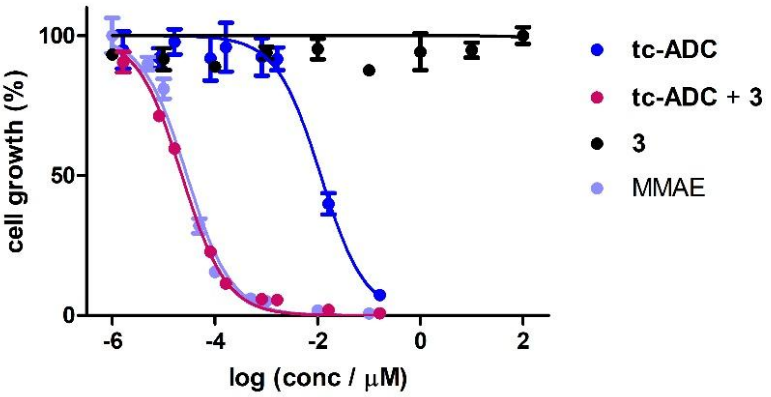

Supplementary Figure 12. Cell proliferation assay. Cell proliferation assay on TAG72-negative HT-29 human colon carcinoma cells treated with **tc-ADC** alone or in combination with activator **3**, activator **3** alone or free MMAE. Data are the mean with s.e.m. (n=3).

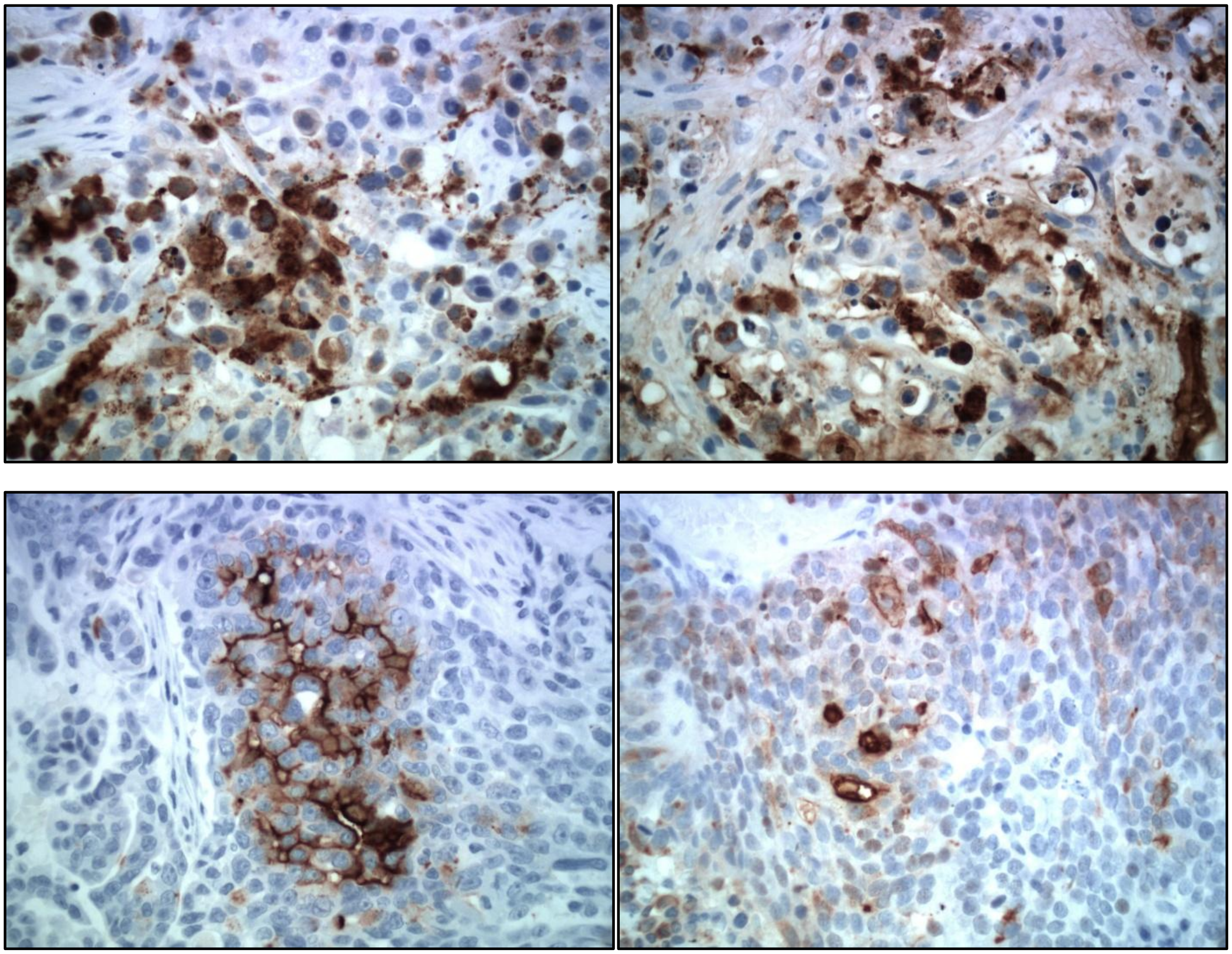

Supplementary Figure 13. Immunohistochemistry. TAG72 expression in (top) two representative LS174T xenograft slices and (bottom) two representative OVCAR-3 xenograft slices. Formaldehyde-fixed LS174T and OVCAR-3 tumours were embedded in paraffin and sliced at 3 µm with a Sakura Accu-Cut SRM microtome and the tumour slices were mounted on Leica Superfrost Plus slides. Sample processing was performed using a Leica BondMax Automated Staining System. The samples were incubated with biotinylated AVP04-58 (1:1600 in Bond Primary Antibody Diluent) for 30 min, washed and then incubated with streptavidin-HRP (BD Pharmingen) for 30 min. Leica Bond polymer was added to the specimens (8 min) followed by tissue peroxidase blocking reagent (5 min) and diaminobenzene (10 min), with washing between each step. The immunostained slices were stained with hematoxylin (5 min), washed, dried, covered and reviewed by an experienced pathologist.

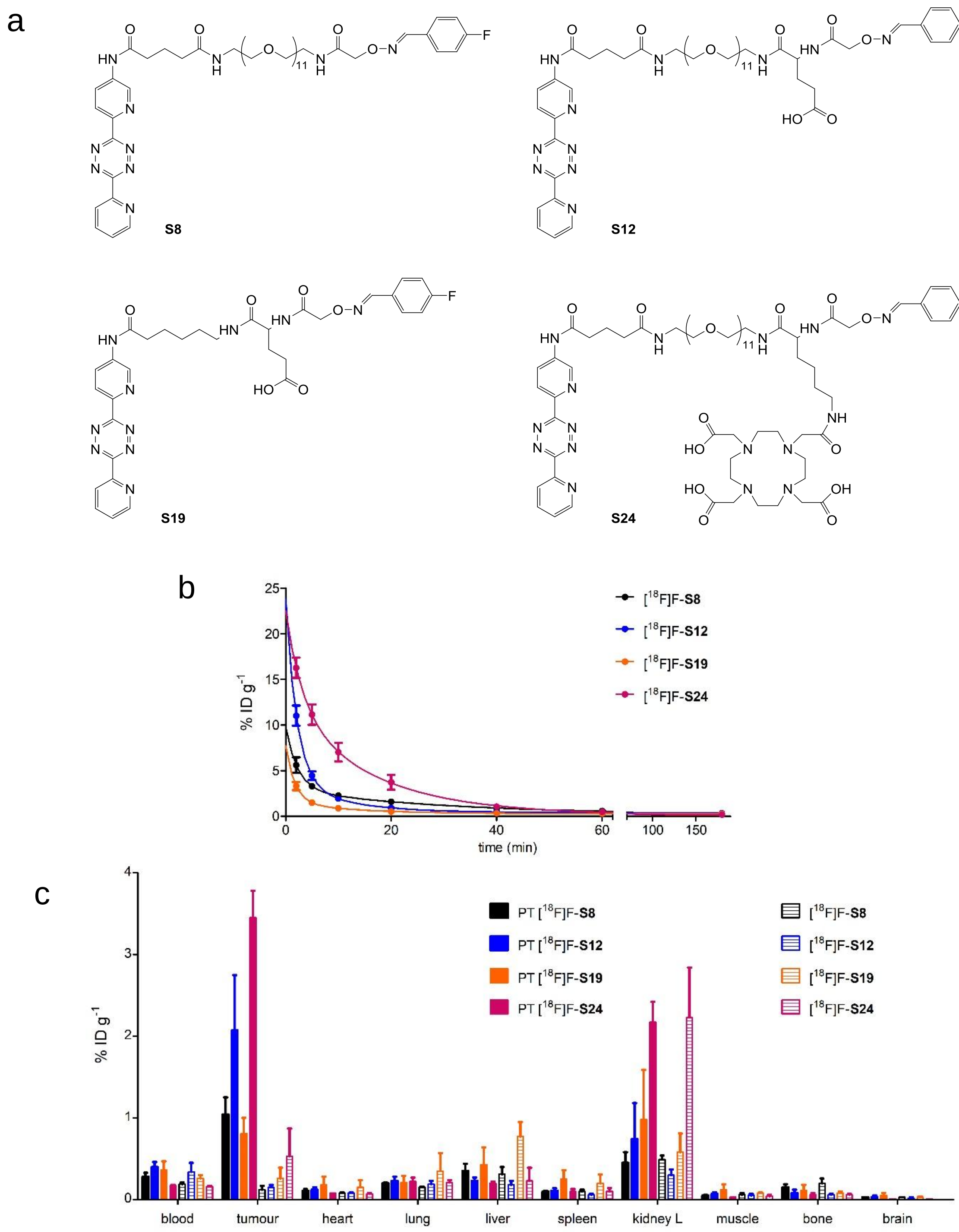

Supplementary Figure 14. In vivo behaviour of ^{18}F-labelled tetrazines. (a) Chemical structures; (b) blood kinetics of ^{18}F-labelled tetrazine probes in LS174T tumour-bearing mice; (c) biodistribution of ^{18}F-labelled tetrazines in LS174T tumour-bearing mice (solid bars: mice pretreated with CC49-TCO; striped bars: non-pretreated mice). Data are the mean % ID g^{-1} with s.d. (n=4). Tetrazines **S8**, **S12** and **S19** exhibited hepatobiliary elimination and most of the injected activity (40-70 % ID) was found in the gut 3 h post-injection. On the contrary, the mice injected with [^{18}F]F-**S24** showed low amounts of radioactivity in the intestine (<3 % ID).

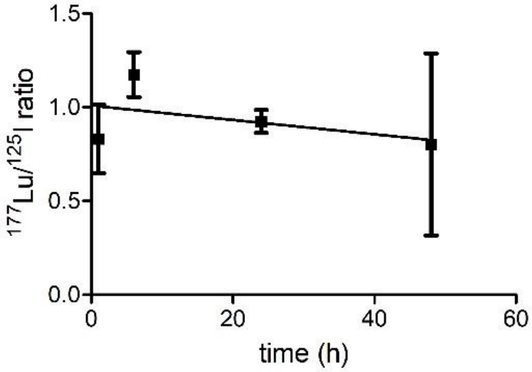

Supplementary Figure 15. In vivo TCO deactivation in blood. Change of ^{177}Lu/^{125}I ratio over time measured after the *ex vivo* reaction of blood samples containing [^{125}I]I-**tc-ADC** with an excess [^{177}Lu]Lu-**4**. Data represent the mean with s.d. (n=4). Blood samples obtained from mice injected with 5 mg kg^{-1} **tc-ADC** were analysed as previously described[5] with minor modifications. Briefly, the samples (15-85 mg) were diluted to 100 mg with PBS and were incubated *ex vivo* (20 min at 37°C) with an excess of no-carrier added [^{177}Lu]Lu-**4** (ca. 0.13 MBq/nmol molar activity). Subsequently 40 µL of each mixture was passed through a Zeba desalting column (40 kDa MW cut-off, 0.5 mL) and the eluate radioactivity was measured in a γ-counter using a dual-isotope protocol (10-40 keV and 155-380 keV energy windows for ^{125}I and ^{177}Lu, respectively, with cross-contamination correction). [^{177}Lu]Lu-**4** in 50% mouse serum was used to correct the values for ^{177}Lu-breakthrough from the Zeba columns (in triplicate). The change in ^{177}Lu/^{125}I cpm ratio over time was used to determine the amount of intact TCO in each blood sample and linear fitting of the data afforded an in vivo TCO deactivation half-life of ca. 5.5 days. This value is in good agreement with the 5 days deactivation half-life that was previously measured for the same TCO linker conjugated to an intact mAb (CC49-TCO-Dox) without PEG[6].

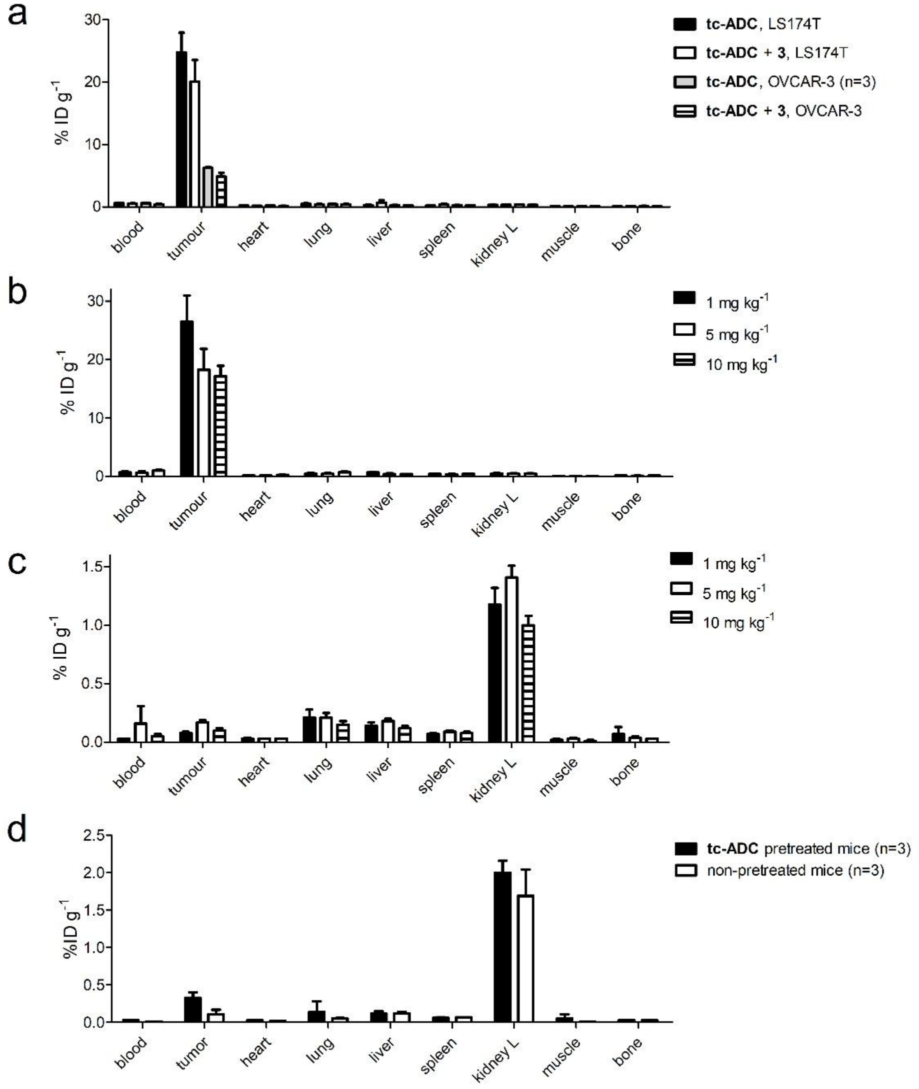

Supplementary Figure 16. Tumour blocking and direct binding studies of the activator. (a) Biodistribution of ^{125}I-labelled **tc-ADC** (2 mg kg^{-1}) in tumour bearing mice with and without activator administration. (b) Biodistribution of ^{125}I-labelled **tc-ADC** injected at different doses (1, 5, and 10 mg kg^{-1}) in LS174T tumour bearing mice. A slight decrease in **tc-ADC** tumour uptake was observed going from 1 to 5 mg kg^{-1} dose (18.27±3.59 *vs.* 26.51±4.49 % ID g^{-1}; $P<0.05$. one-way ANOVA with Bonferroni's post-test) while no further decrease was observed at a 10 mg kg^{-1} dose. The absolute amount of tumour-bound TCO increased with the increasing injected ADC mass (0.11 nmol, 0.49 nmol and 0.53 nmol at 1, 5 and 10 mg kg^{-1} **tc-ADC** dose, respectively. (c) Biodistribution of [^{177}Lu]Lu-**4** in mice that received different doses of **tc-ADC** (1, 5, and 10 mg kg^{-1}) followed by 0.335 mmol kg^{-1} activator **3** in LS174T tumour bearing mice. Despite the increase in local TCO concentration, activator **3** effectively blocked the subsequent uptake of probe **4** in all groups. (d) Biodistribution of [^{177}Lu]Lu-**3** at low dose (13 nmol kg^{-1}) in LS174T tumour-bearing mice pretreated with **tc-ADC** or non-pretreated. The tumour uptake of 0.33±0.07 % ID g^{-1} for ^{177}Lu-labelled **3** in mice pretreated with **tc-ADC** was significantly higher than the nonspecific [^{177}Lu]Lu-**3** retention in the tumour of non-pretreated mice (0.11±0.06 % ID g^{-1}; P = 0.0145, t-test). Data represent the mean % ID g^{-1} with s.d. (n=3-4).

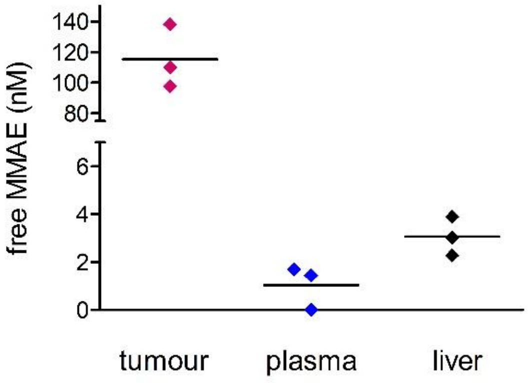

Supplementary Figure 17. In vivo concentration of free MMAE. MMAE concentration in tumor, plasma and liver samples harvested from mice that were injected with 2 mg kg^{-1} **vc-ADC** and euthanized 24 h later.

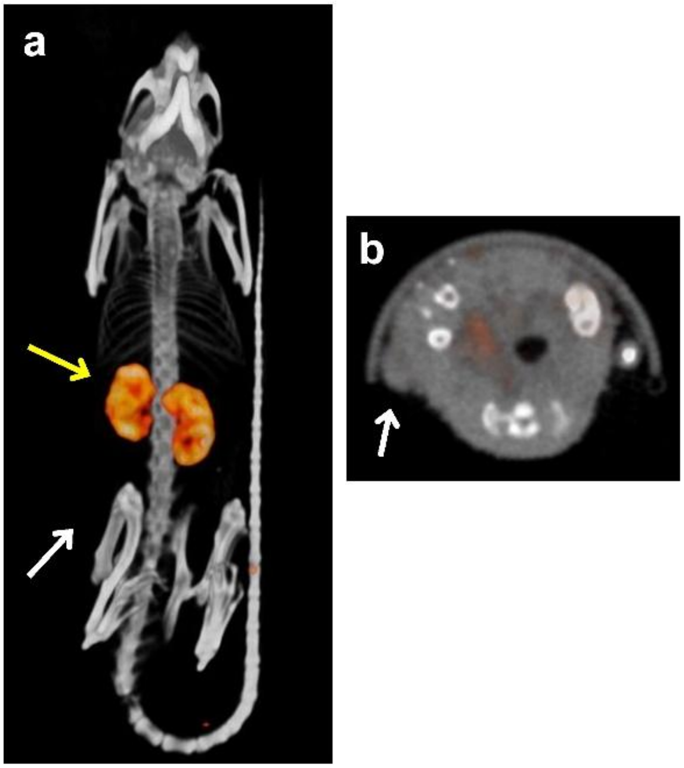

Supplementary Figure 18. Small-animal imaging of control mouse. (a) Post-mortem SPECT/CT projection of a LS174T tumour-bearing mouse injected with ^{111}In-labelled activator precursor **S4** (ca. 13 nmol kg^{-1}, 13 MBq) without **tc-ADC** pretreatment; the mouse was euthanized 3 h post-activator injection and the bladder was voided before imaging. (b) Single transverse slice passing through the tumour of the mouse in (a) showing no radioactivity uptake in the tumour. White arrows indicate the tumour; yellow arrow indicates the kidneys.

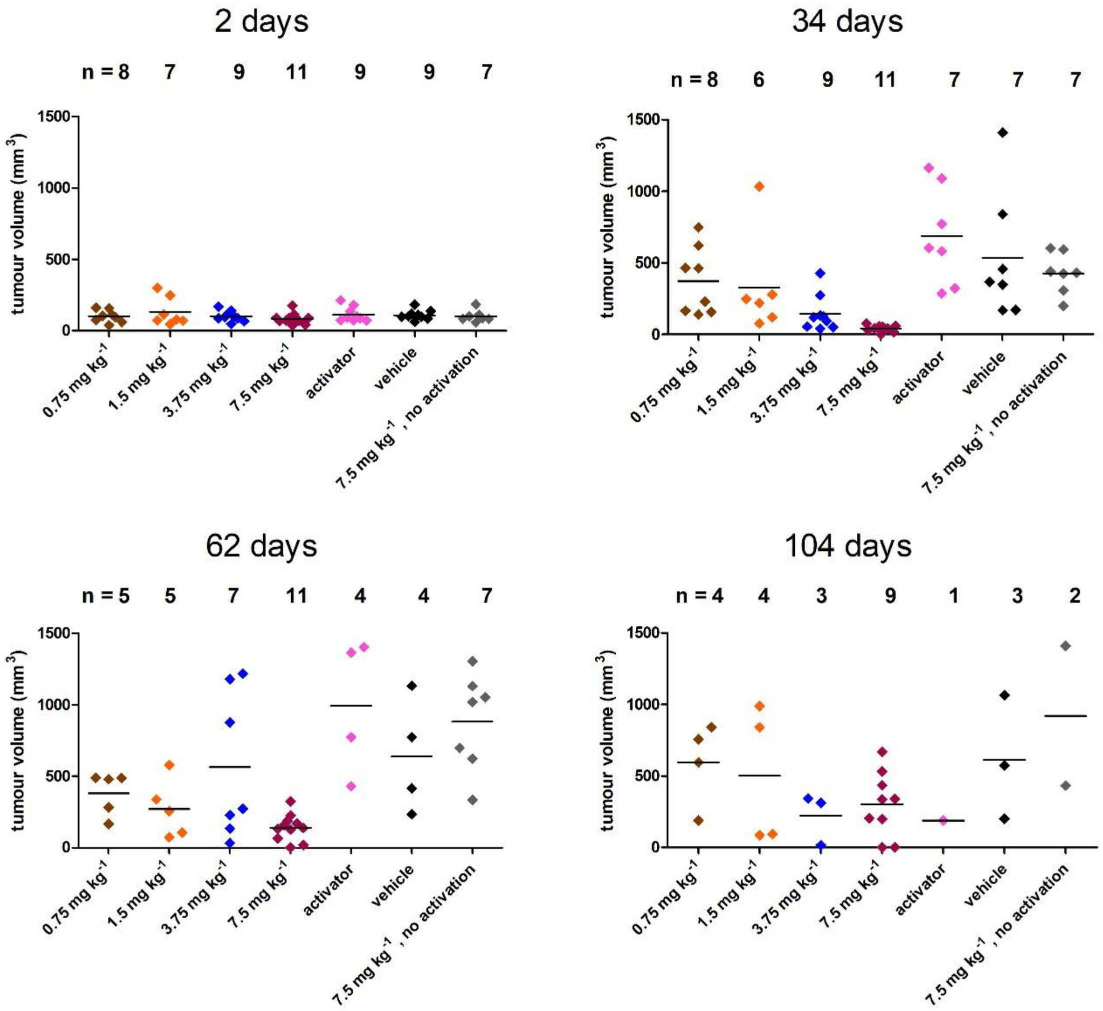

Supplementary Figure 19. Pilot therapy study. Single-dose therapy study in mice bearing OVCAR-3 xenografts (n=7-11) administered with increasing doses of **tc-ADC** (0.75,1.5, 3.75, or 7.5 mg kg^{-1} in 100 µL PBS containing 5% DMSO) followed by activator **3** (0.335 mmol kg^{-1} in 130 µL PBS containing 5% DMSO) 48 h later, and controls: **tc-ADC** (7.5 mg kg^{-1}) followed by vehicle (n=7), vehicle followed by activator (n=9), or two vehicle injections (n=9); tumour volumes and remaining group sizes at selected times post treatment. Despite heterogeneous tumour growth, a dose-dependent therapeutic effect was observed in the 4 groups of mice that received **tc-ADC** followed by activator**.**

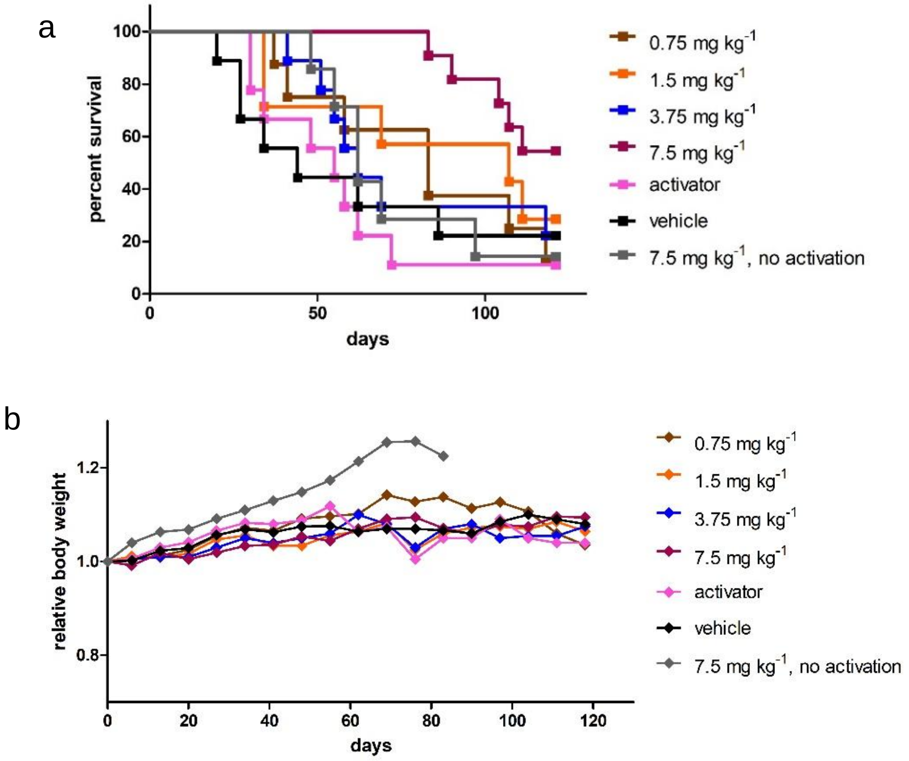

Supplementary Figure 20. Pilot therapy study. (a) Survival plots for the single-dose therapy study in OVCAR-3 bearing mice. The median survival increased from 44 days (vehicle group) to 83 and 107 days for the groups that received, respectively, 0.75 and 1.5 mg kg^{-1} **tc-ADC** followed by activator **3** (Supplementary Table 7). A shorter median survival (62 days) was found at the next dose level (3.75 mg kg^{-1} **tc-ADC** + **3**), possibly due to complications arising from the tumour burden rather than the treatment. (b) Body weight changes (with respect to day 0) of OVCAR-3 bearing mice treated with one dose of **tc-ADC** and activator **3** or controls. Relative body weight data represent the mean; error bars were omitted for clarity. No weight losses were observed in the first weeks after treatment in the mice that received 0.75-3.75 mg kg^{-1} **tc-ADC** and **3**, while some of these mice showed signs of poor health and were removed from the study 1.5-2 months after treatment (Supplementary Table 7). A similar trend was observed in the 7.5 mg kg^{-1} **tc-ADC** with activator group, where all mice tolerated the treatment well and 6 out of 11 mice survived till the end of the study (with 2 complete remissions), while 4 mice were removed from the study in the last month due to poor general health.

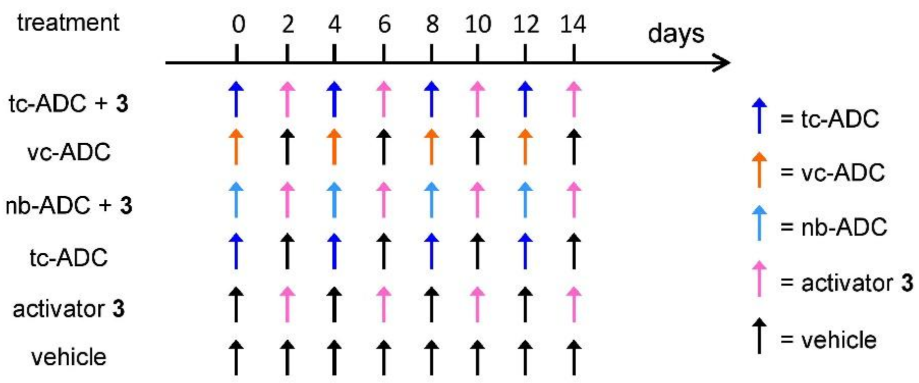

Supplementary Figure 21. Injection dose scheme. Time line of injections in the multi-dose efficacy studies in OVCAR-3 and LS174T tumour models.

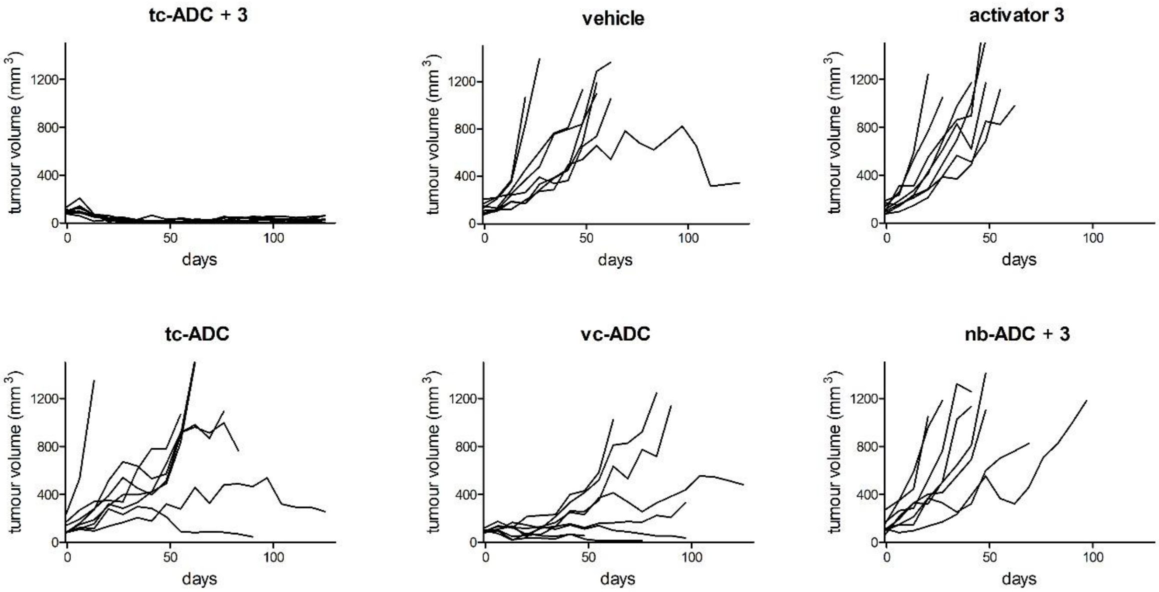

Supplementary Figure 22. Single-mouse tumour growth curves. Tumour growth in single OVCAR-3 bearing mice in the multi-dose therapeutic study (mean tumour volumes presented in Fig. 6d and 6e). The group of mice that received **tc-ADC** and activator showed significant tumour regression in the first weeks after treatment (117±46 mm^3 and 18±9 mm^3 tumour volumes at 6 and 34 days, respectively; P=0.0004, paired t-test) followed by 3 months with barely palpable residual tumour masses.

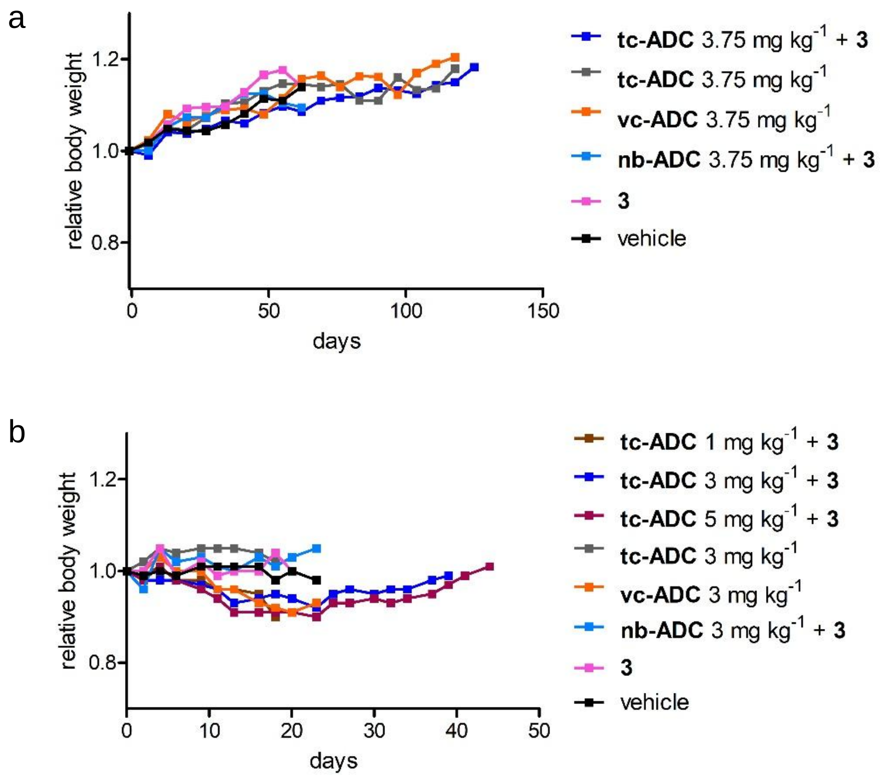

Supplementary Figure 23. Mouse body weights during the multi-dose therapeutic studies. (a) Body weight changes (with respect to day 0) of OVCAR-3 bearing mice treated with multiple doses of **tc-ADC** and activator **3** or controls. Data represent the mean; error bars were omitted for clarity. Overall, repeated doses of **tc-ADC** and **3** were well tolerated by the mice and only one mouse was removed from the study during the last month because of poor health. On the contrary, 4/8 mice treated with **vc-ADC** were euthanized in the second half of the study due to poor general health or extreme weight losses (Supplementary Table 8). (b) Body weight changes of LS174T bearing mice treated with multiple doses of **tc-ADC** and **3** or controls. Also in this study **tc-ADC** and **3** were generally well tolerated by the mice. The plot suggests that mice treated with the **vc-ADC** or the combination of **tc-ADC** with **3** lost body weight during the two weeks treatment while the other groups (vehicle, **nb-ADC** followed by **3**, or **tc-ADC** and **3** alone) maintained a stable weight during this time. However, the apparent weight stability of the control animals was due to the development of very large tumours contributing to the total weight of the mice. Similarly, the apparent weight recovery of the treated mice (**tc-ADC + 3**) was the effect of tumour growth. In general all groups bearing this very aggressive tumour line experienced an approx. 10% weight loss excluding tumour weight during the study, most likely due to the tumour burden, but showed no signs of discomfort. The exception is the group of mice treated with repeated 3 mg kg^{-1} doses of **vc-ADC**, which were euthanized in the first month due to poor physical health (Supplementary Table 9).

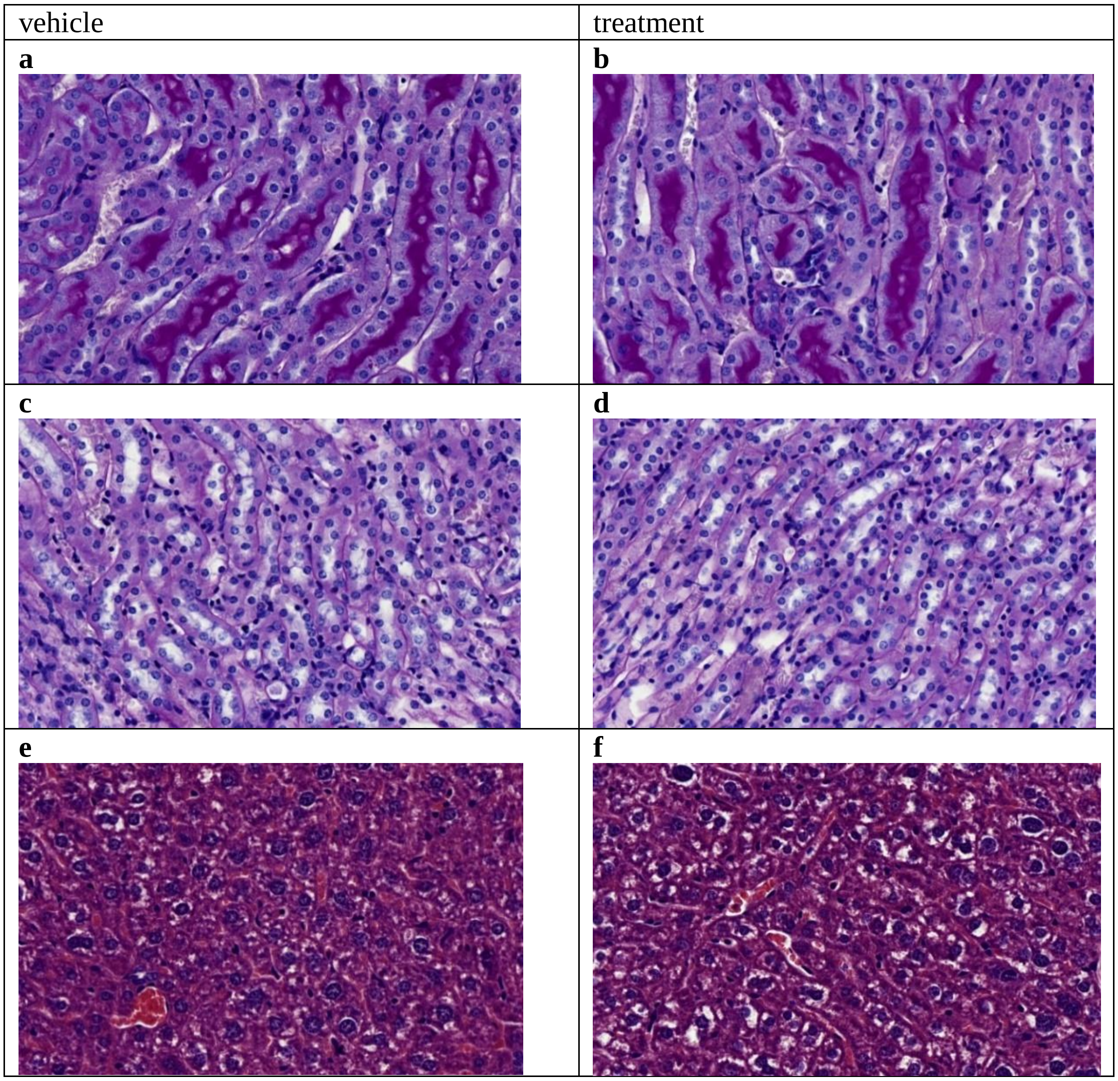

Supplementary Figure 24. Histopathology: representative slices of (a, b) renal cortex, (c, d) renal medulla, and (e, f) liver from tumour-free mice injected with four cycles of **tc-ADC** (5 mg kg^{-1}) and activator **3** (0.335 mmol kg^{-1}) or with vehicle. Kidney and liver slices stained with periodic-acid Schiff and hematoxylin&eosin, respectively and examined by a pathologist. No signs of renal damage (i.e. tubules dilation, basal membrane thickening or mitotic activity)[7] and no signs of liver damage were observed, as illustrated by the high degree of similarity between vehicle and treatment group.

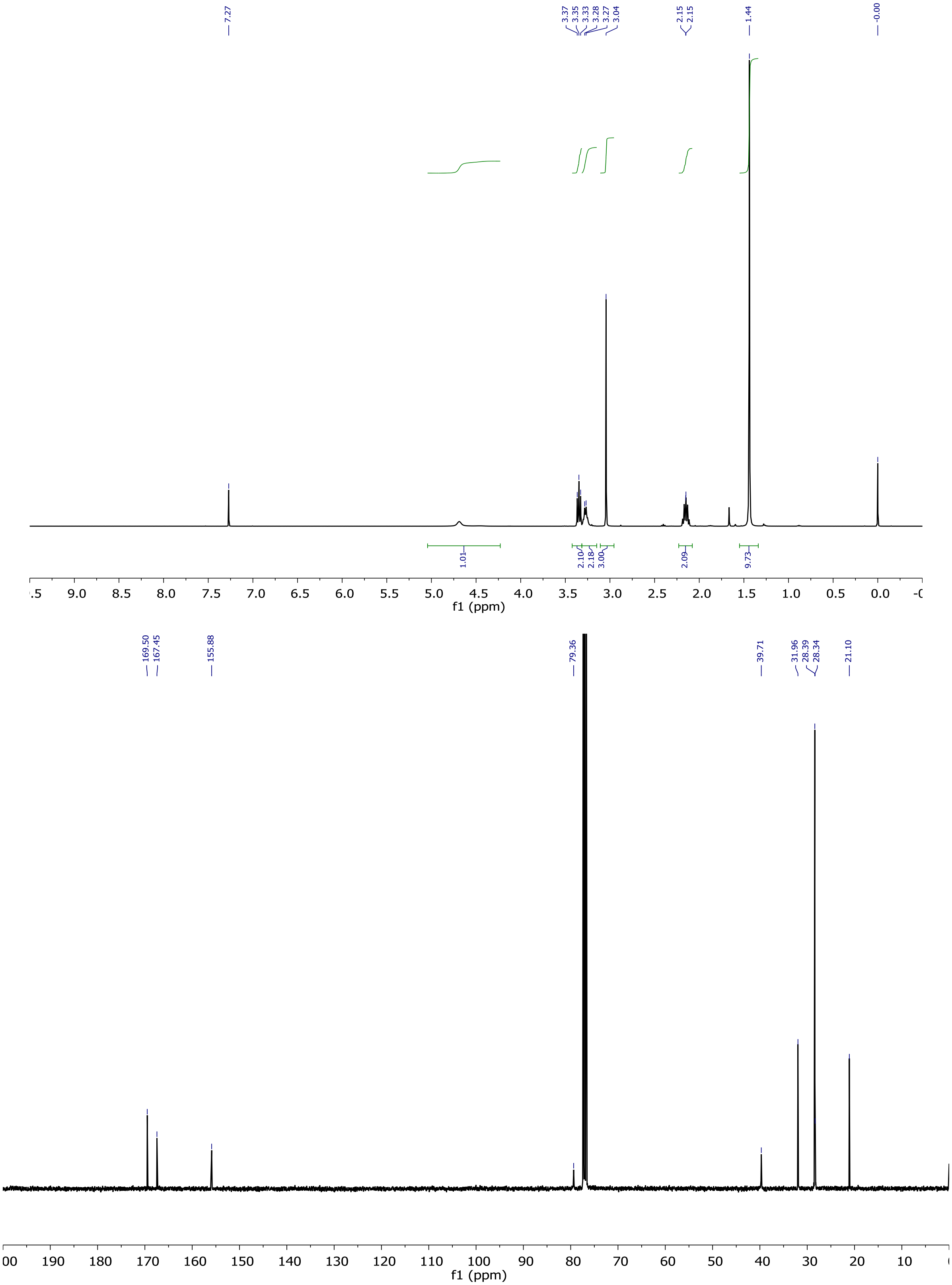

Supplementary Figure 25. (Top) ^{1}H and (bottom) ^{13}C spectra ($CDCl_3$) of compound S1.

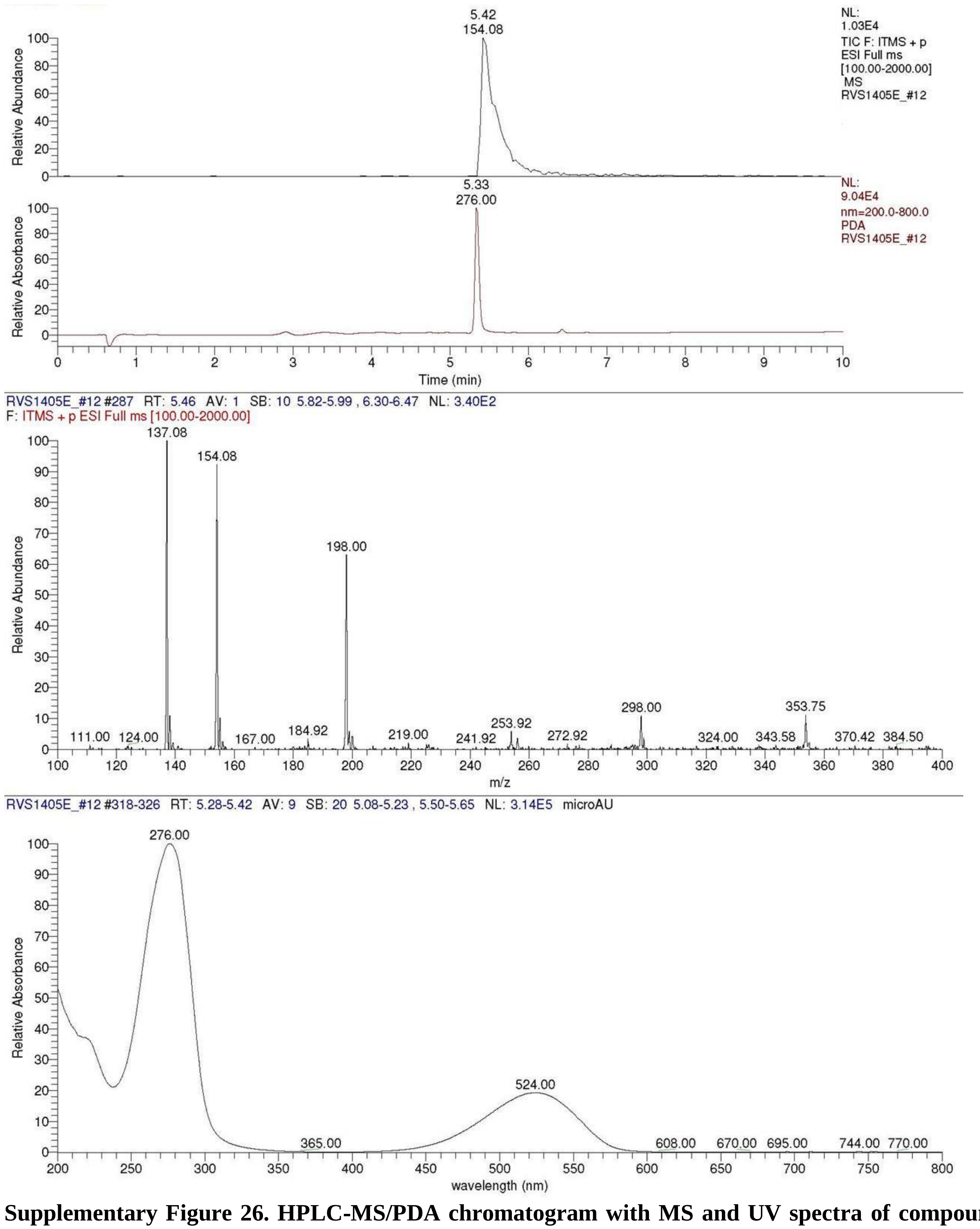

Supplementary Figure 26. HPLC-MS/PDA chromatogram with MS and UV spectra of compound S1.

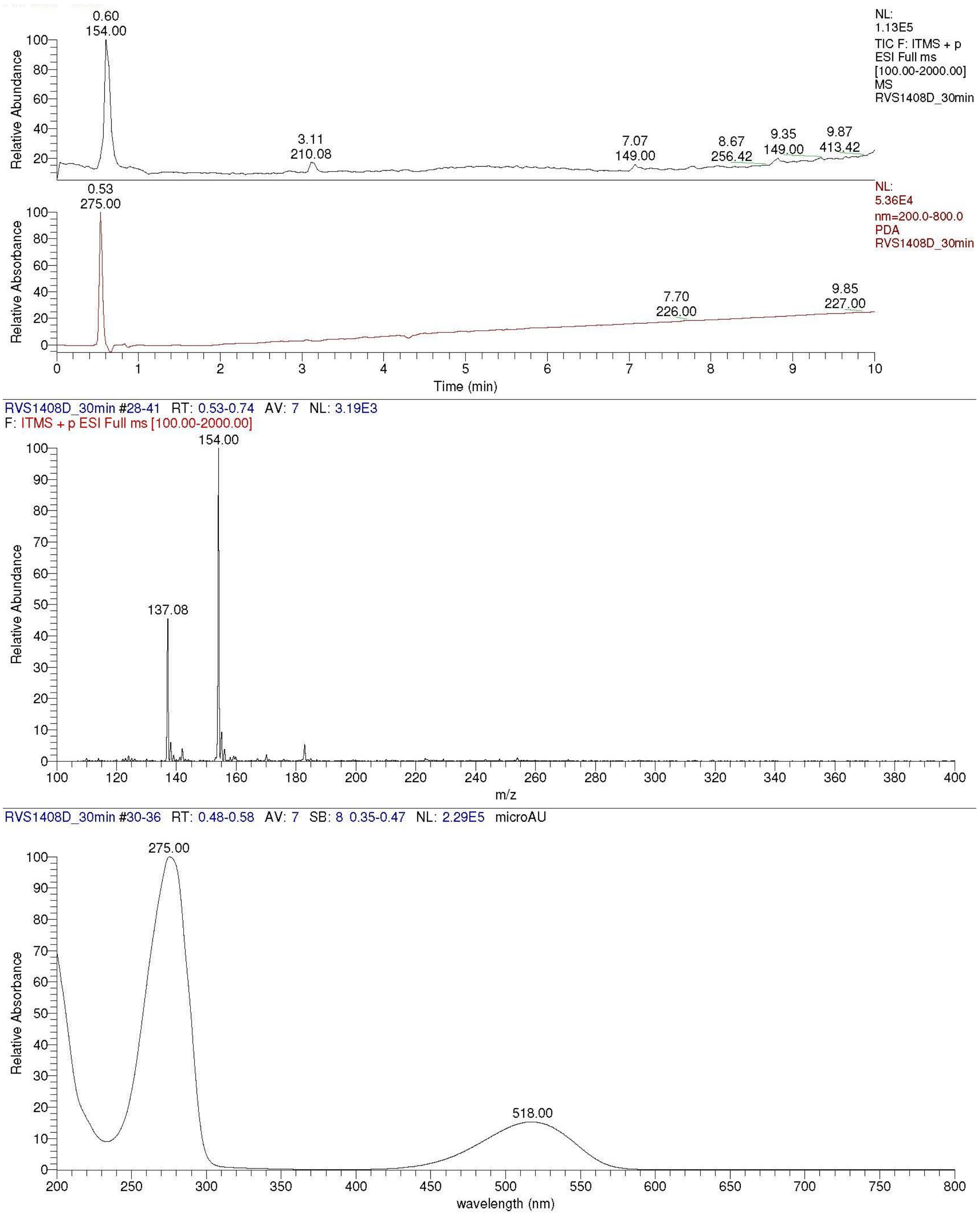

Supplementary Figure 27. HPLC-MS/PDA chromatogram with MS and UV spectra of deprotected compound S1.

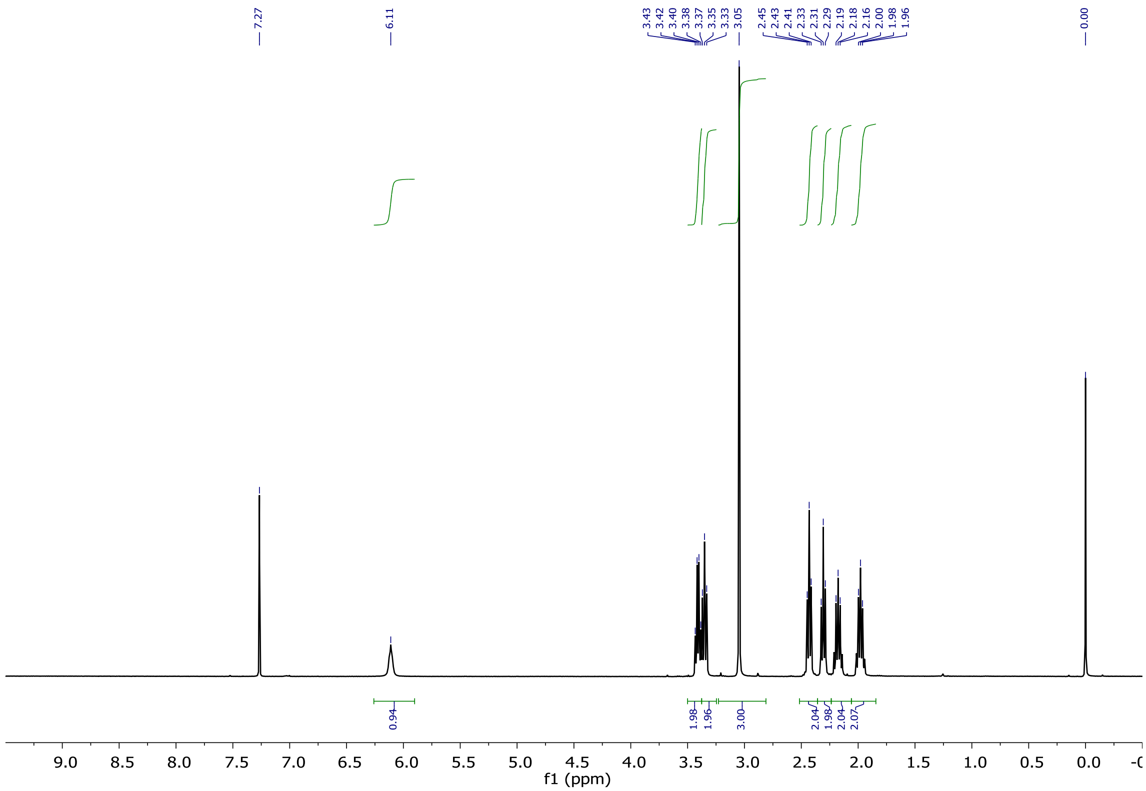

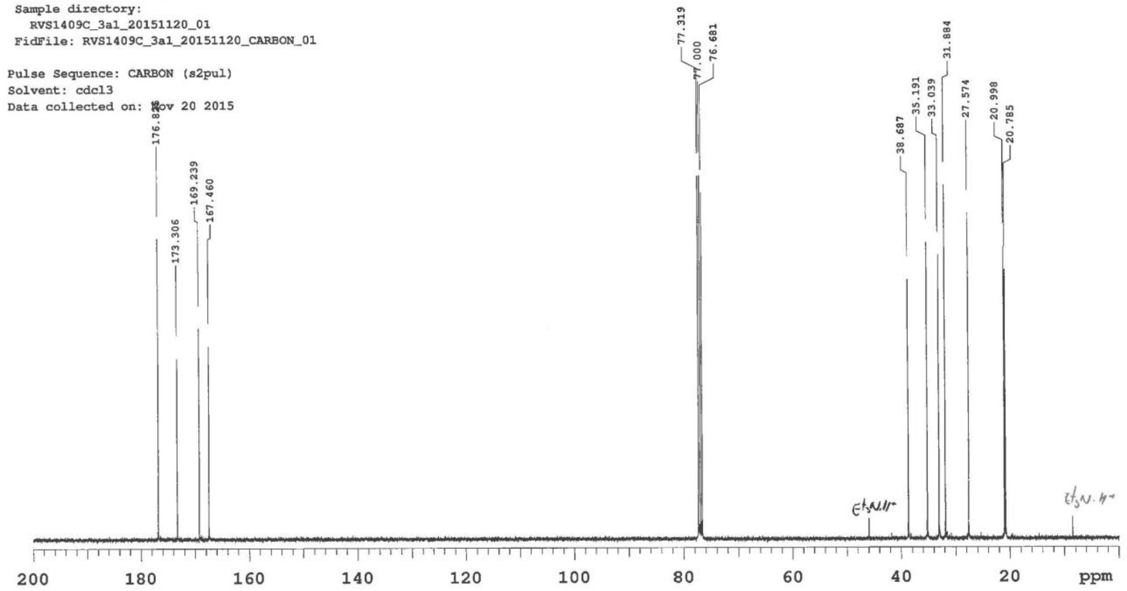

Supplementary Figure 28. (Top) ^{1}H and (bottom) ^{13}C NMR spectra ($CDCl_3$) of compound S2.

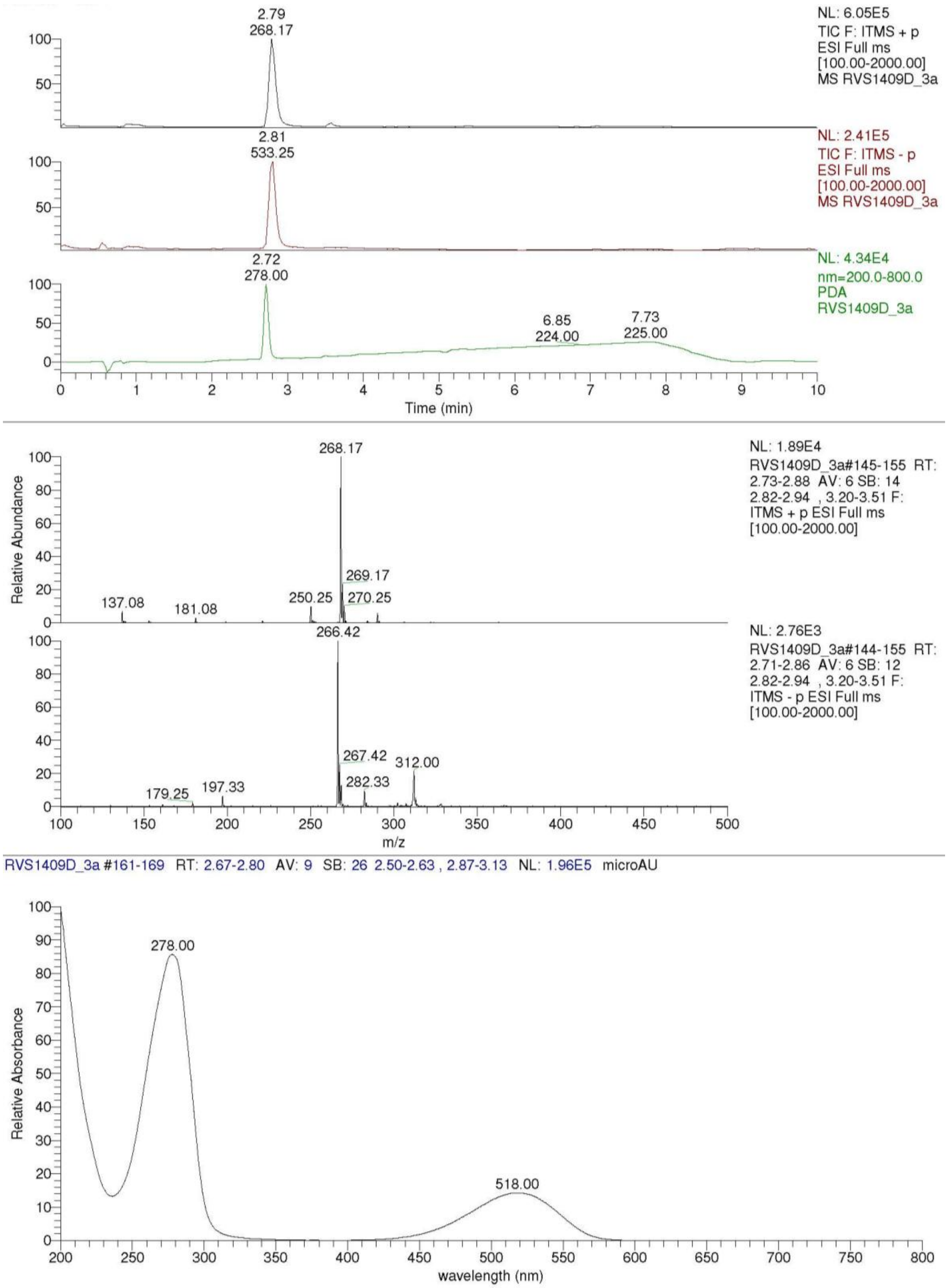

Supplementary Figure 29. HPLC-MS/PDA chromatogram with MS and UV spectra of compound S2.

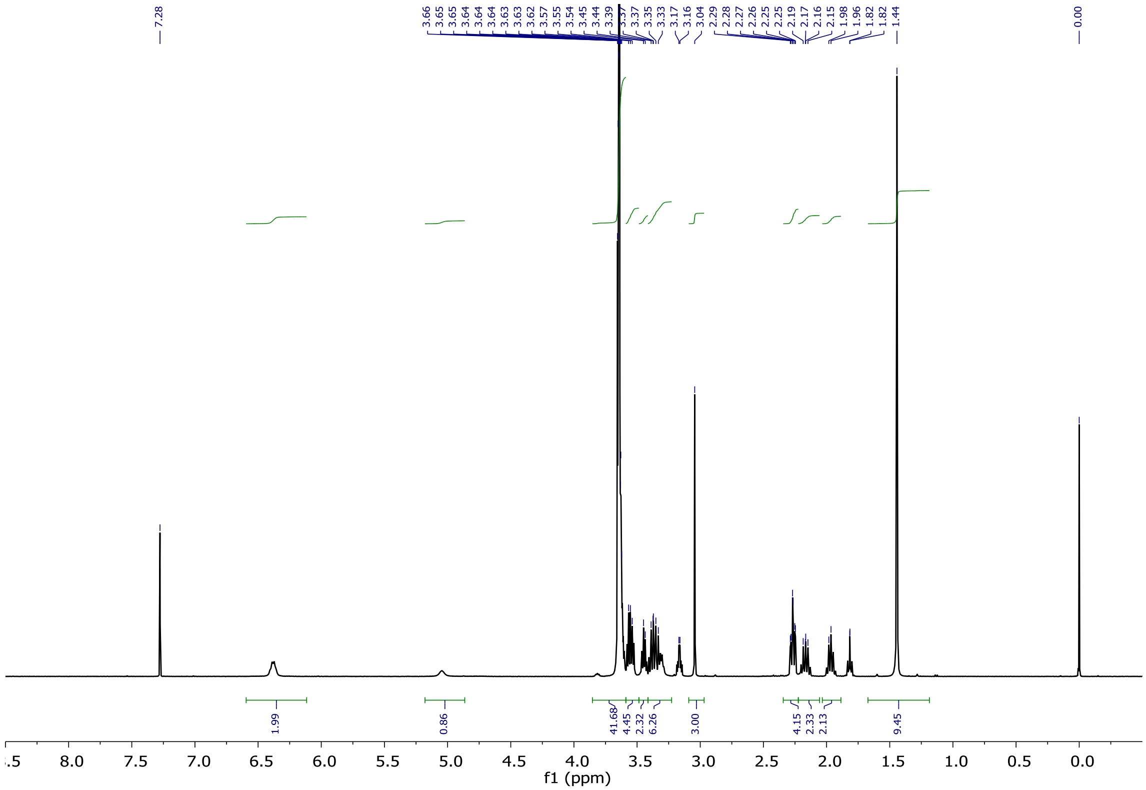

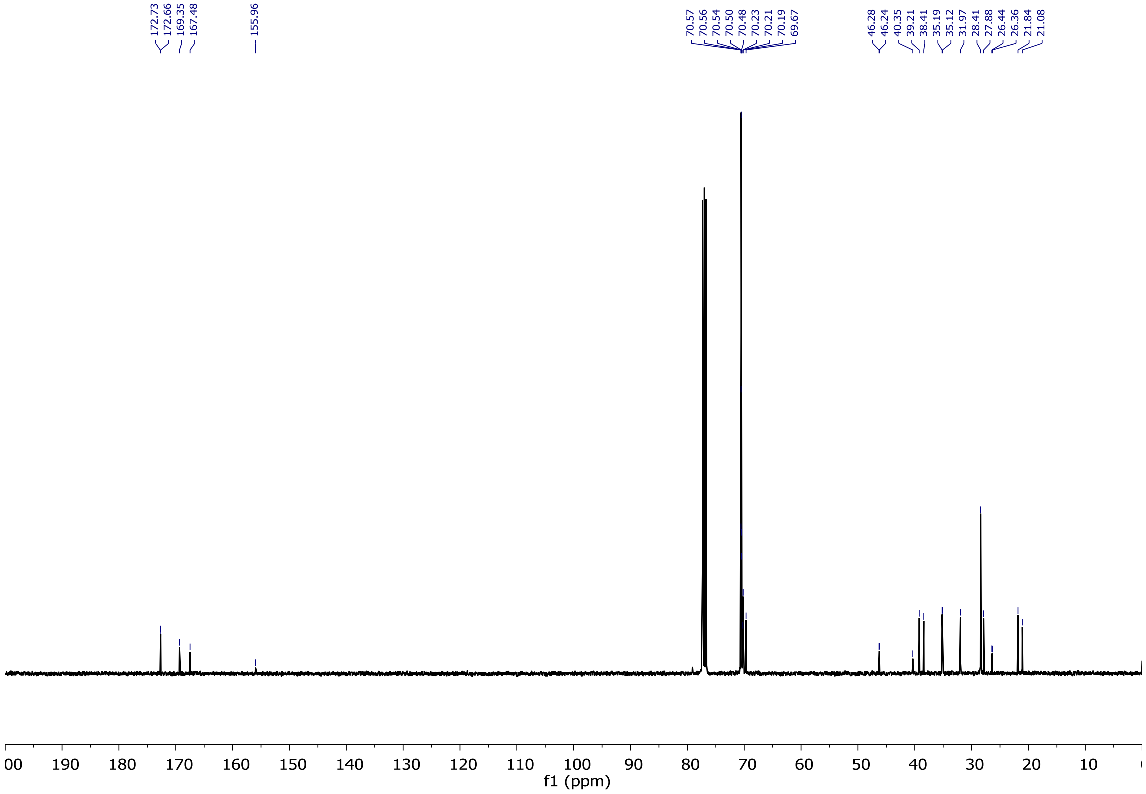

Supplementary Figure 30. (Top) ^{1}H and (bottom) ^{13}C NMR spectra ($CDCl_3$) of compound S3.

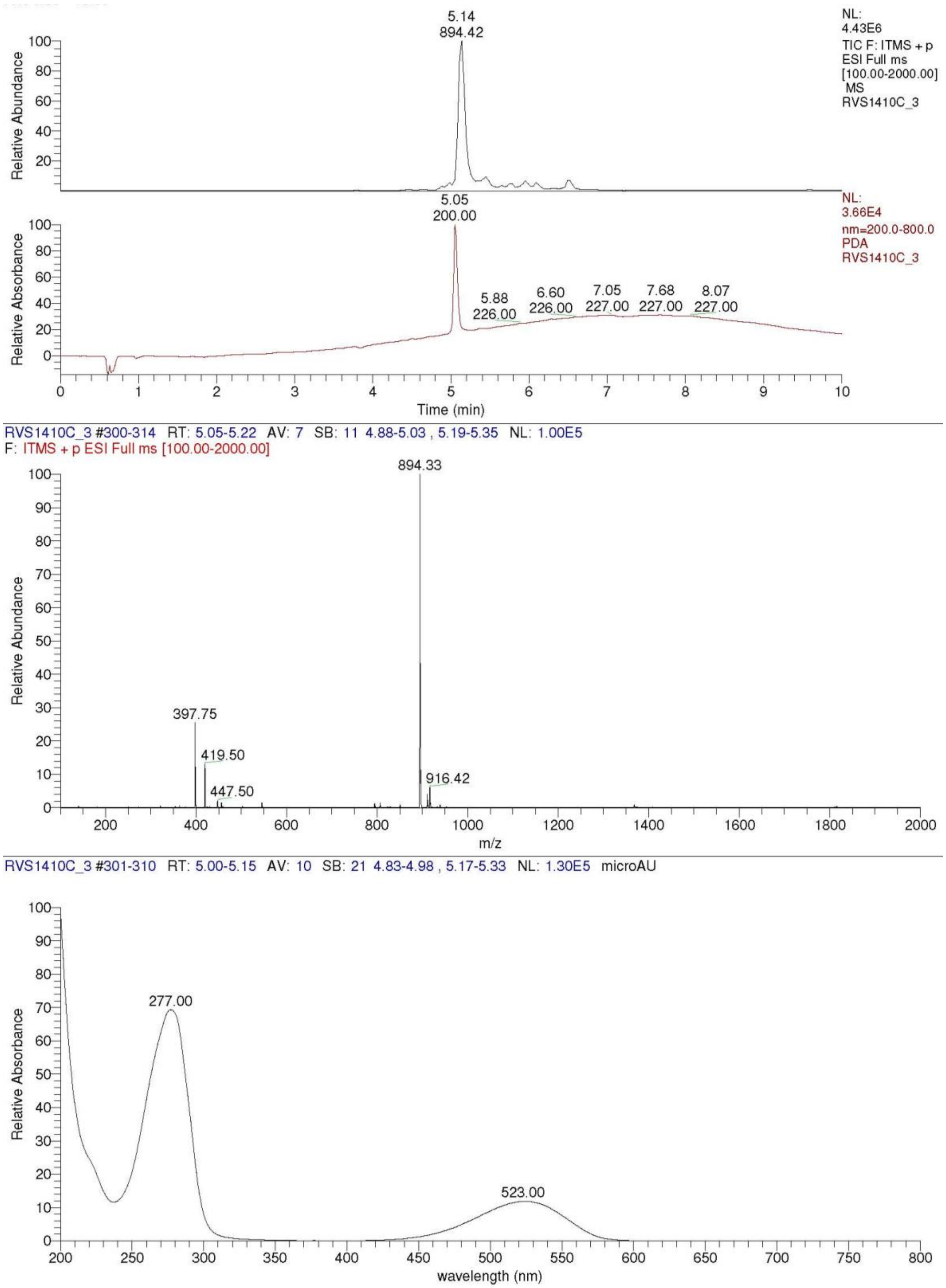

Supplementary Figure 31. HPLC-MS/PDA chromatogram with MS and UV spectra of compound S3.

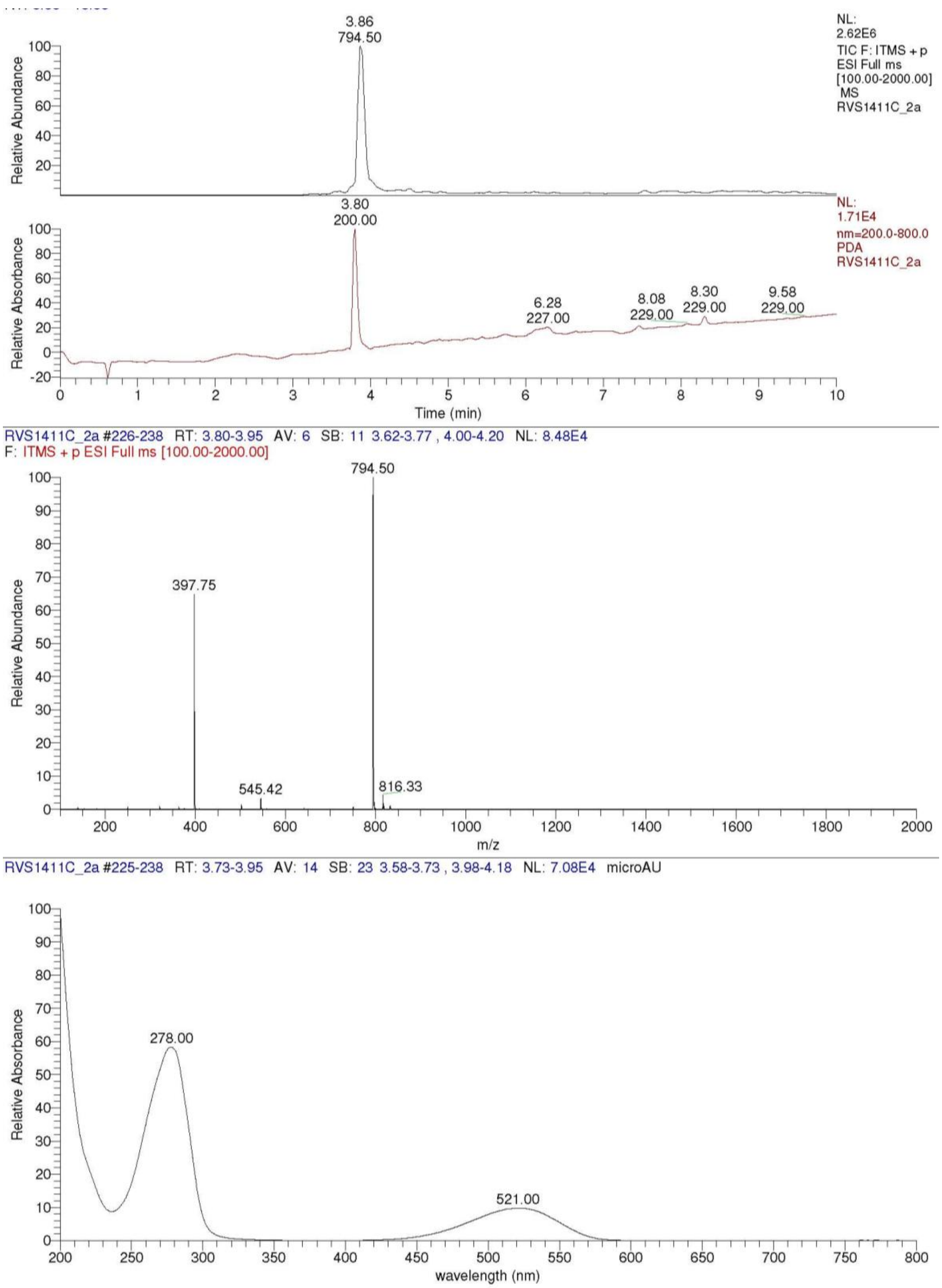

Supplementary Figure 32. HPLC-MS/PDA chromatogram with MS and UV spectra of deprotected compound S3.

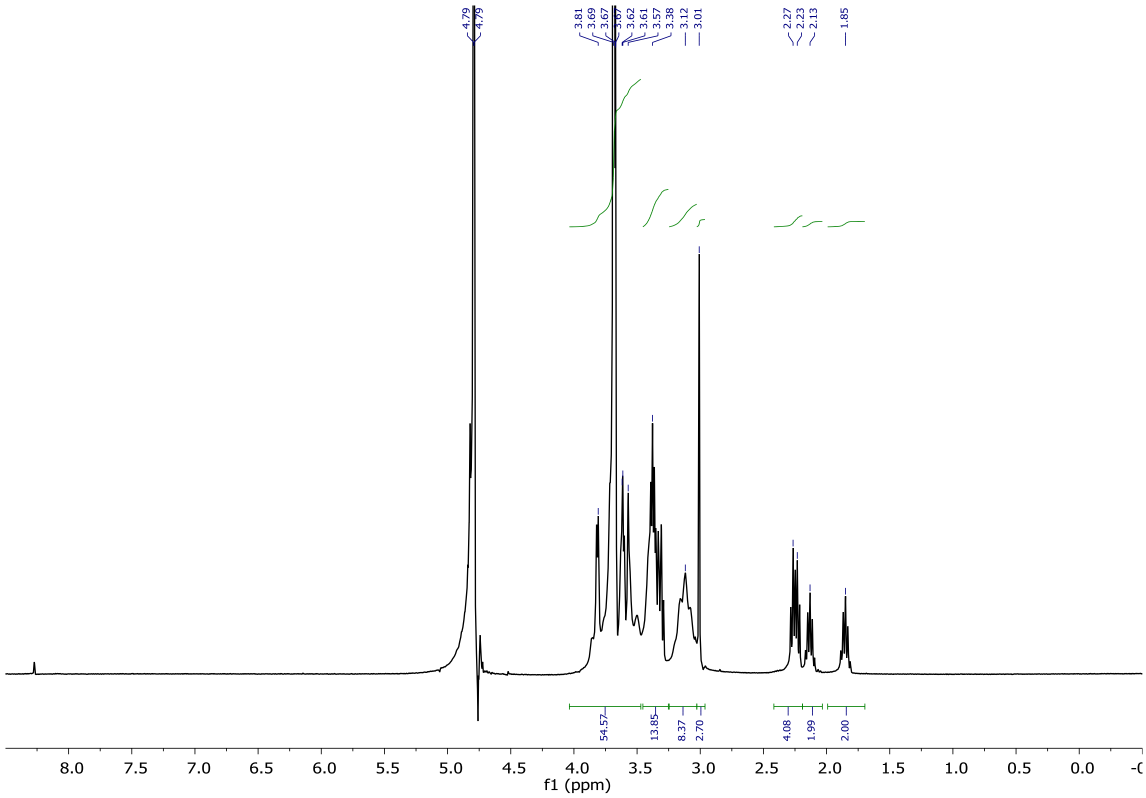

Supplementary Figure 33. ^{1}H NMR spectrum (D_2O) of compound S4.

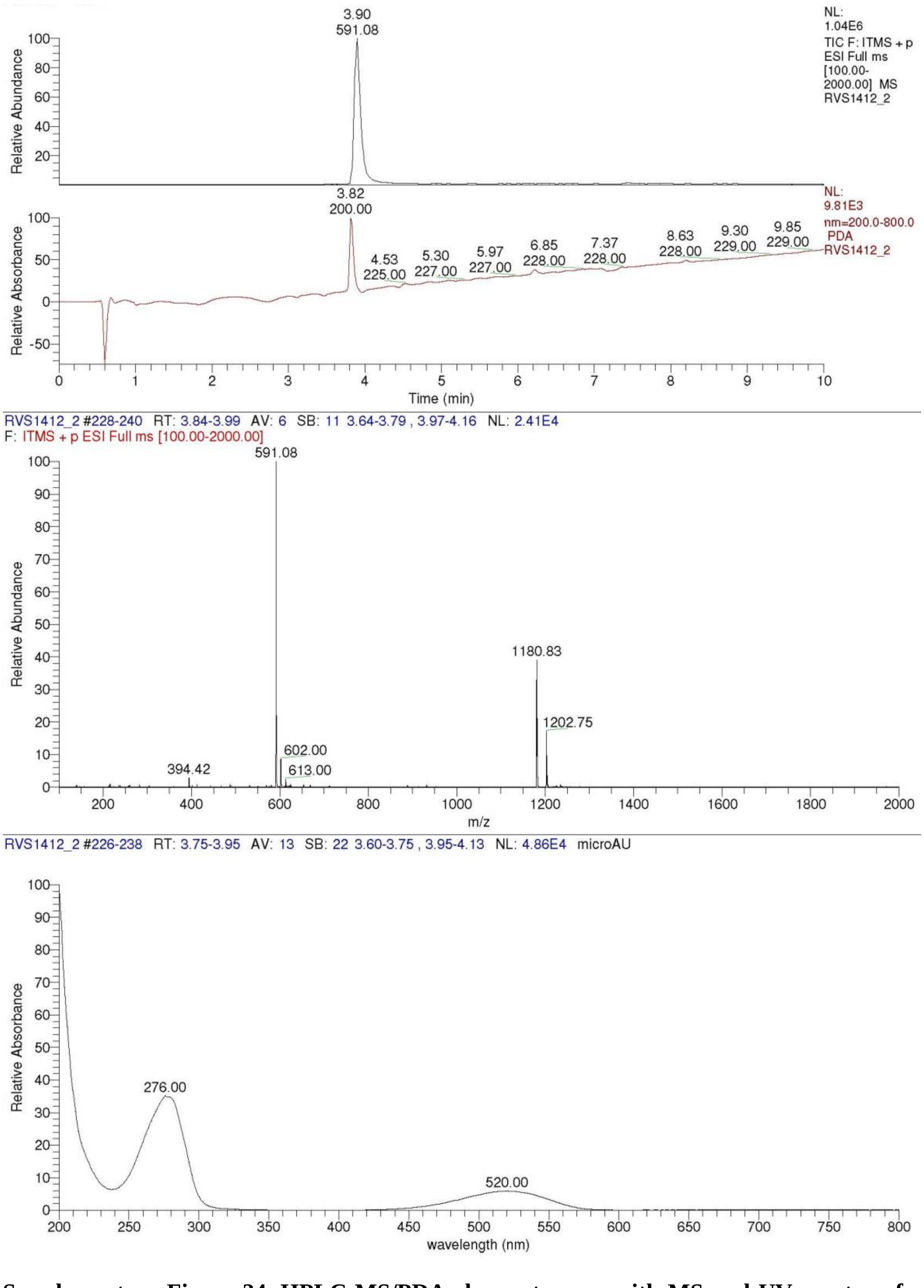

Supplementary Figure 34. HPLC-MS/PDA chromatogram with MS and UV spectra of compound S4.

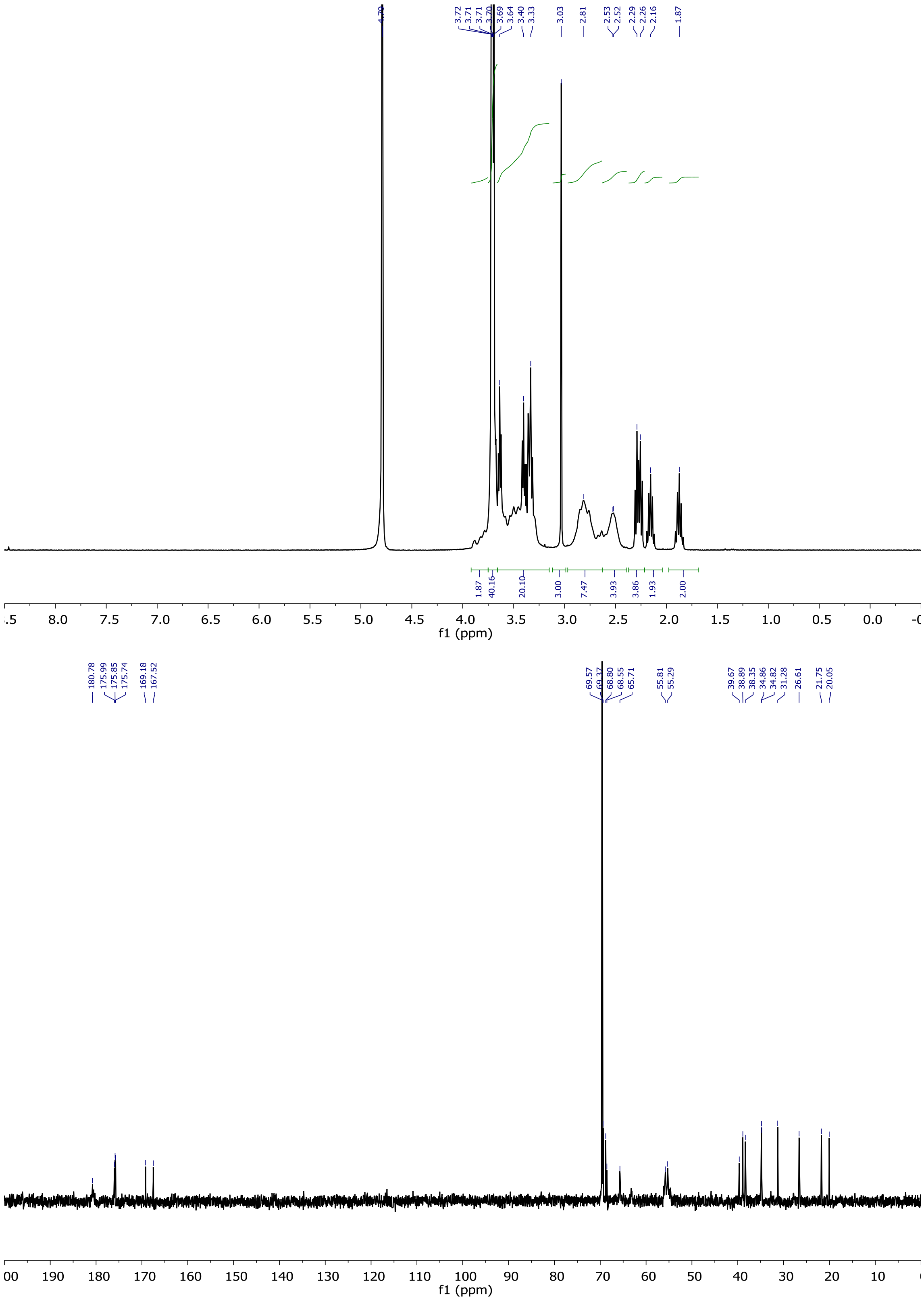

Supplementary Figure 35. (Top) ^{1}H and (bottom) ^{13}C NMR spectra (D_2O) of compound 3.

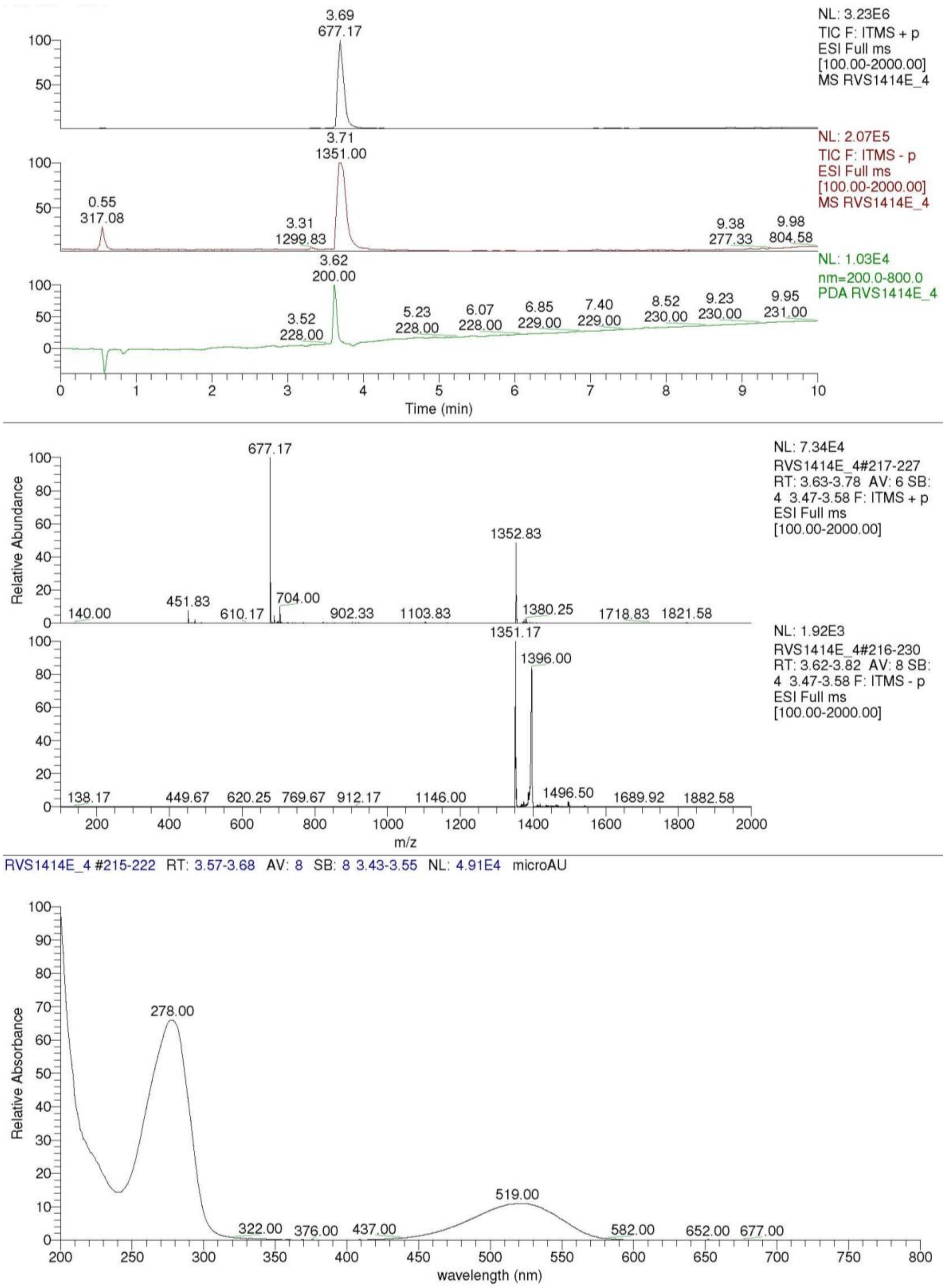

Supplementary Figure 36. HPLC-MS/PDA chromatogram with MS and UV spectra of compound 3.

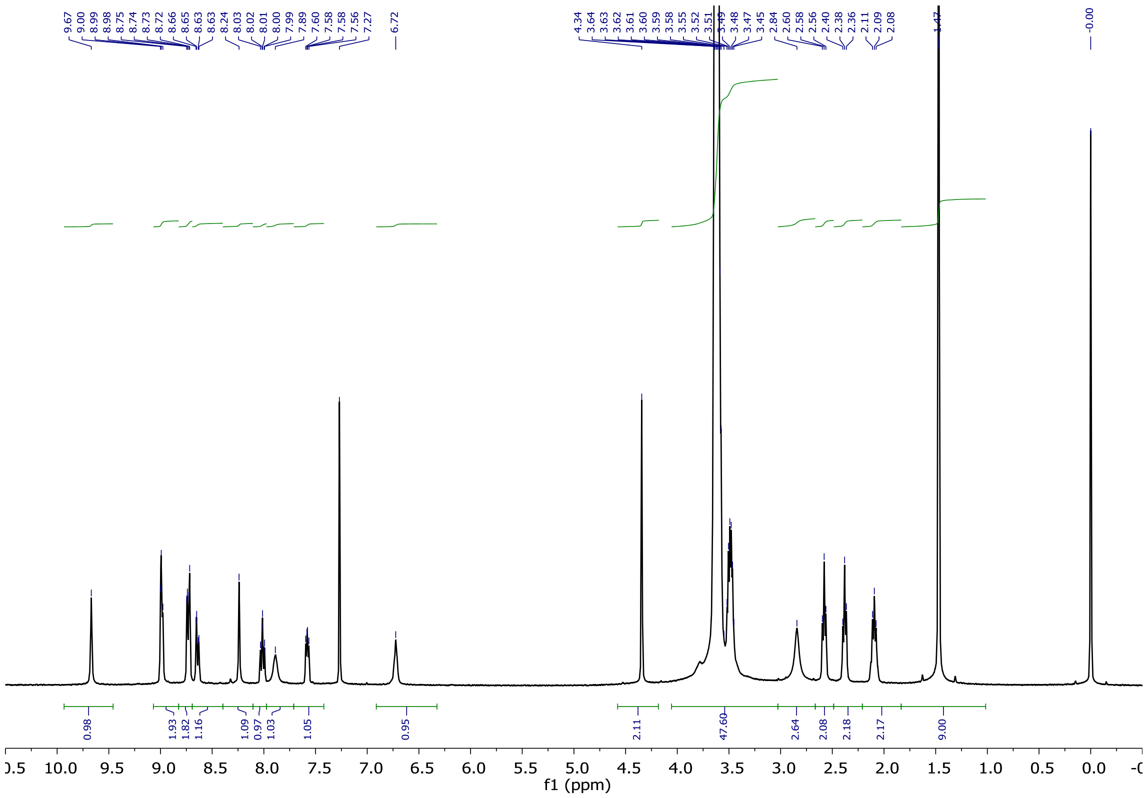

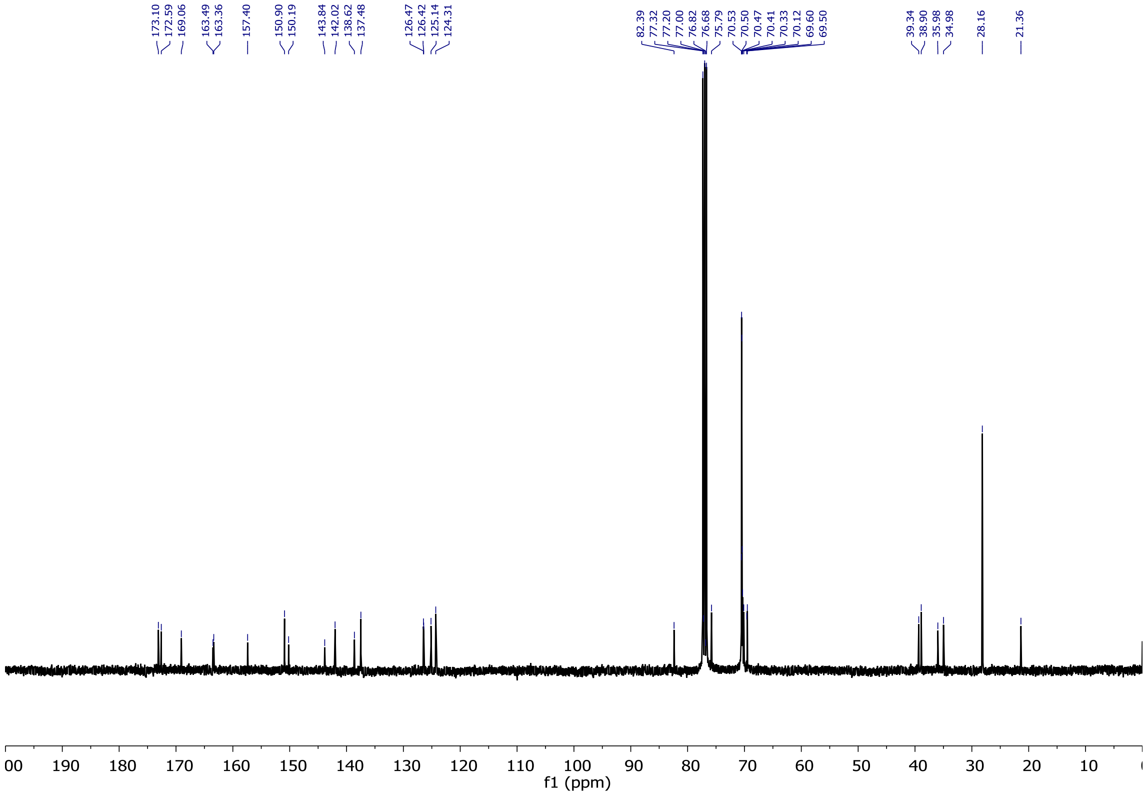

Supplementary Figure 37. (Top) ^{1}H and (bottom) ^{13}C NMR spectra ($CDCl_3$) of compound S6.

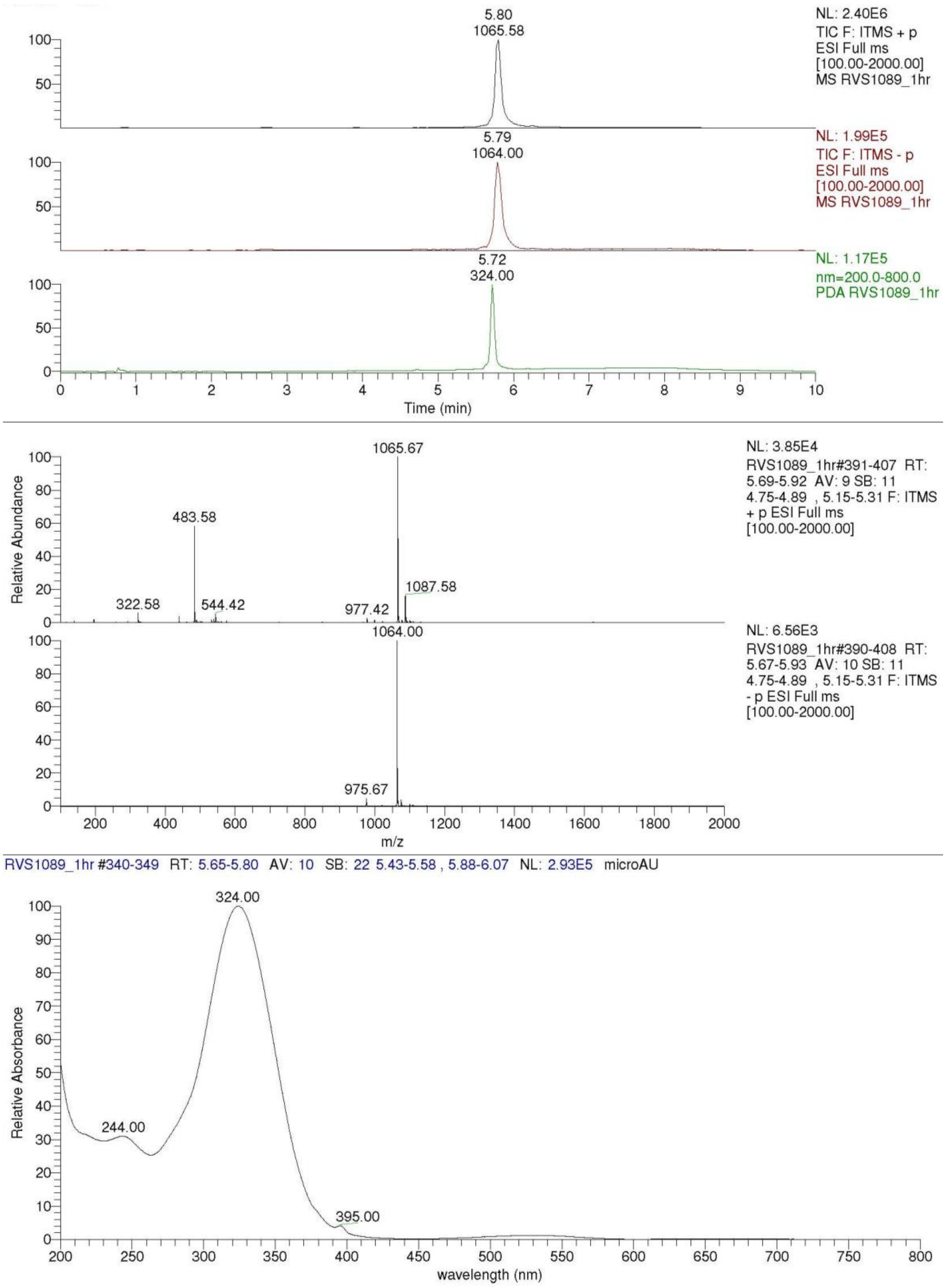

Supplementary Figure 38. HPLC-MS/PDA chromatogram with MS and UV spectra of compound S6.

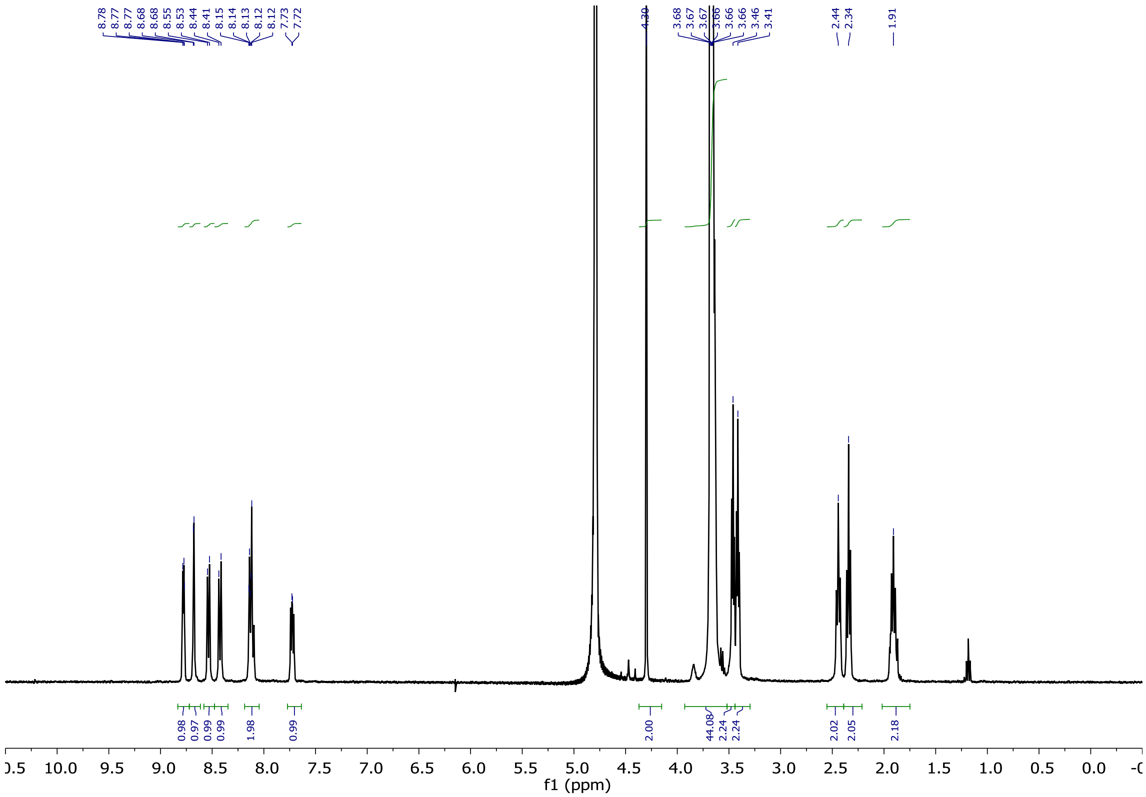

Supplementary Figure 39. ^{1}H NMR spectrum (D_2O) of compound S7.

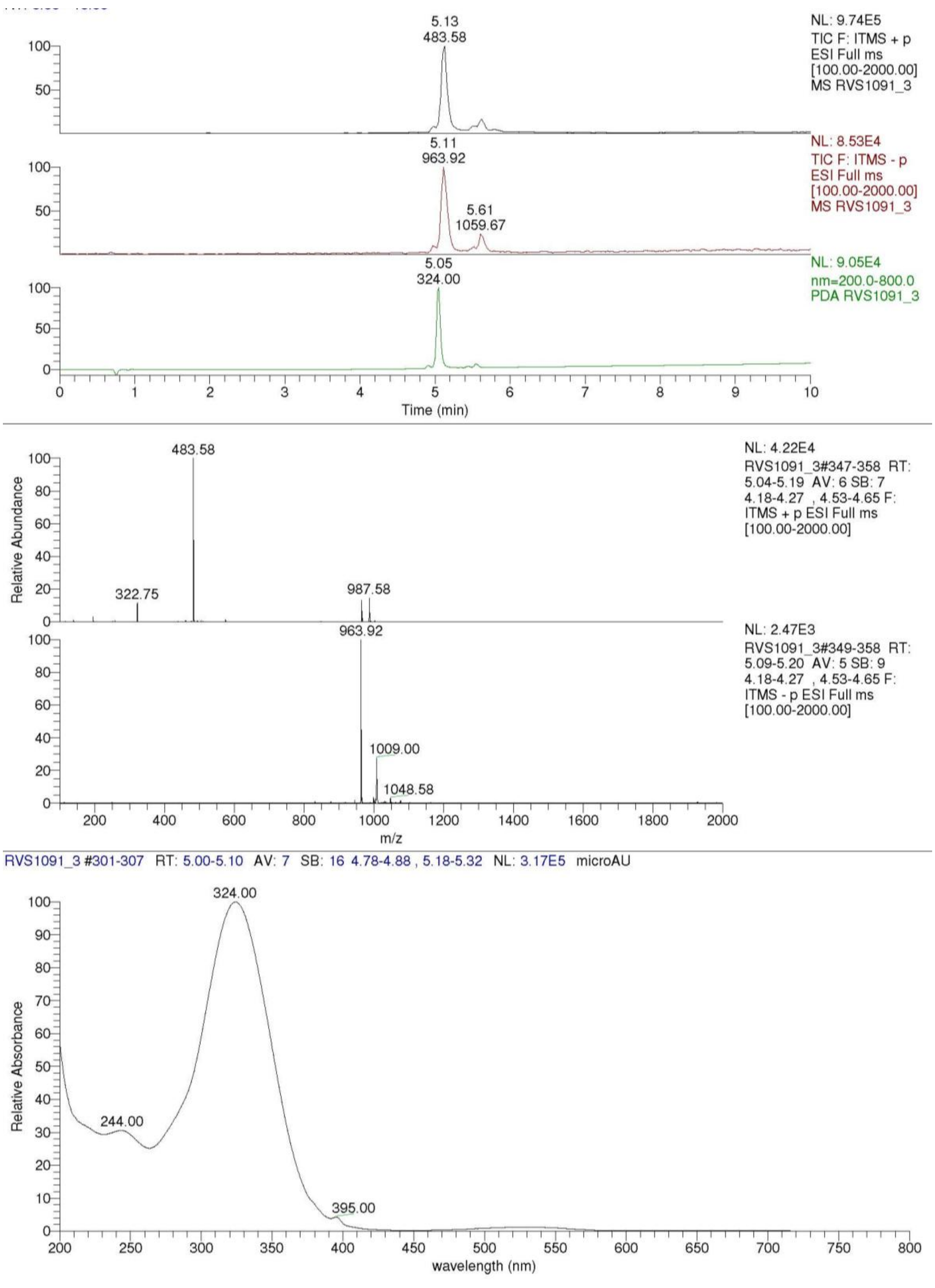

Supplementary Figure 40. HPLC-MS/PDA chromatogram with MS and UV spectra of compound S7.

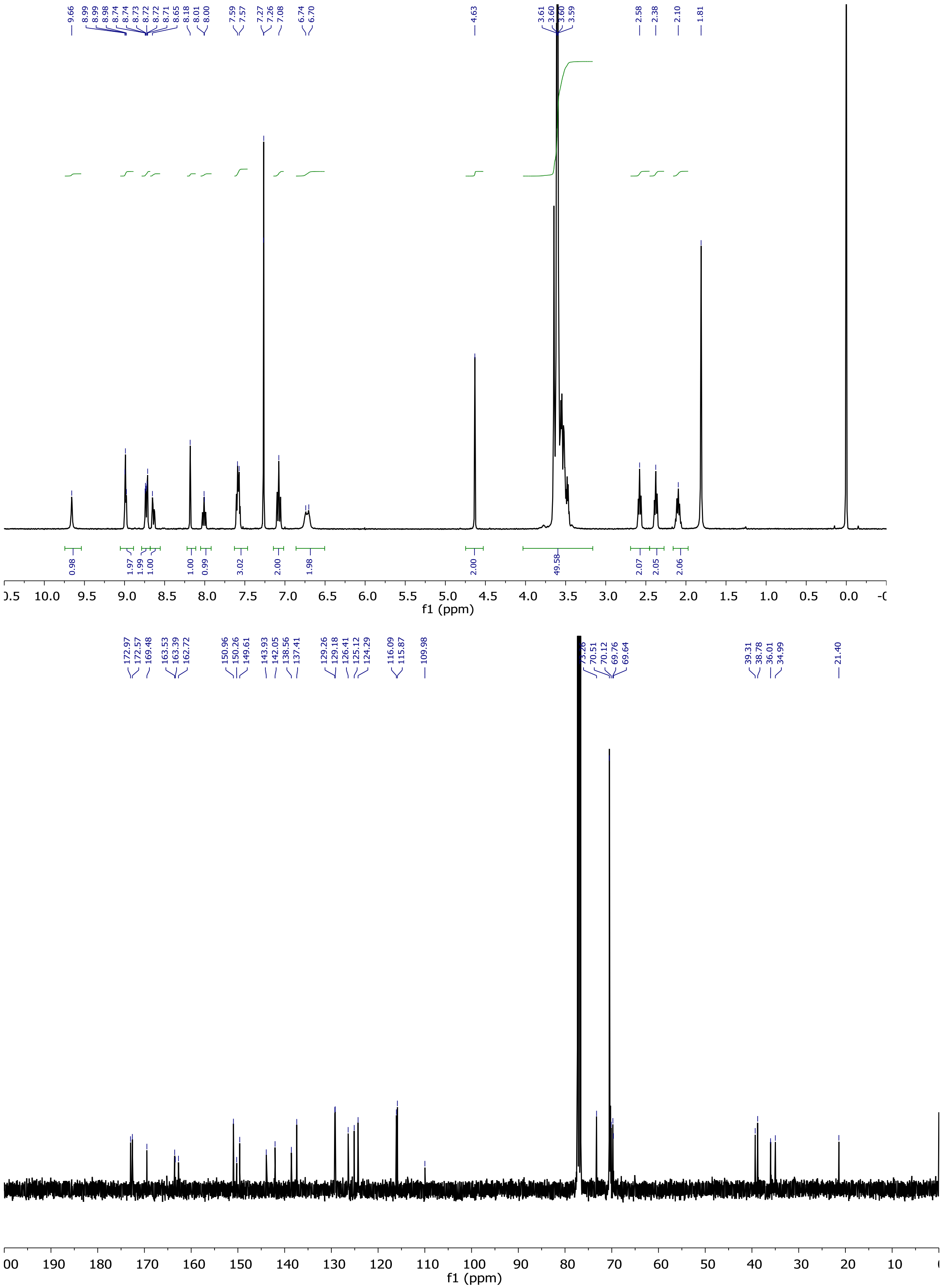

Supplementary Figure 41. (Top) ^{1}H and (bottom) ^{13}C NMR spectra ($CDCl_3$) of compound S8.

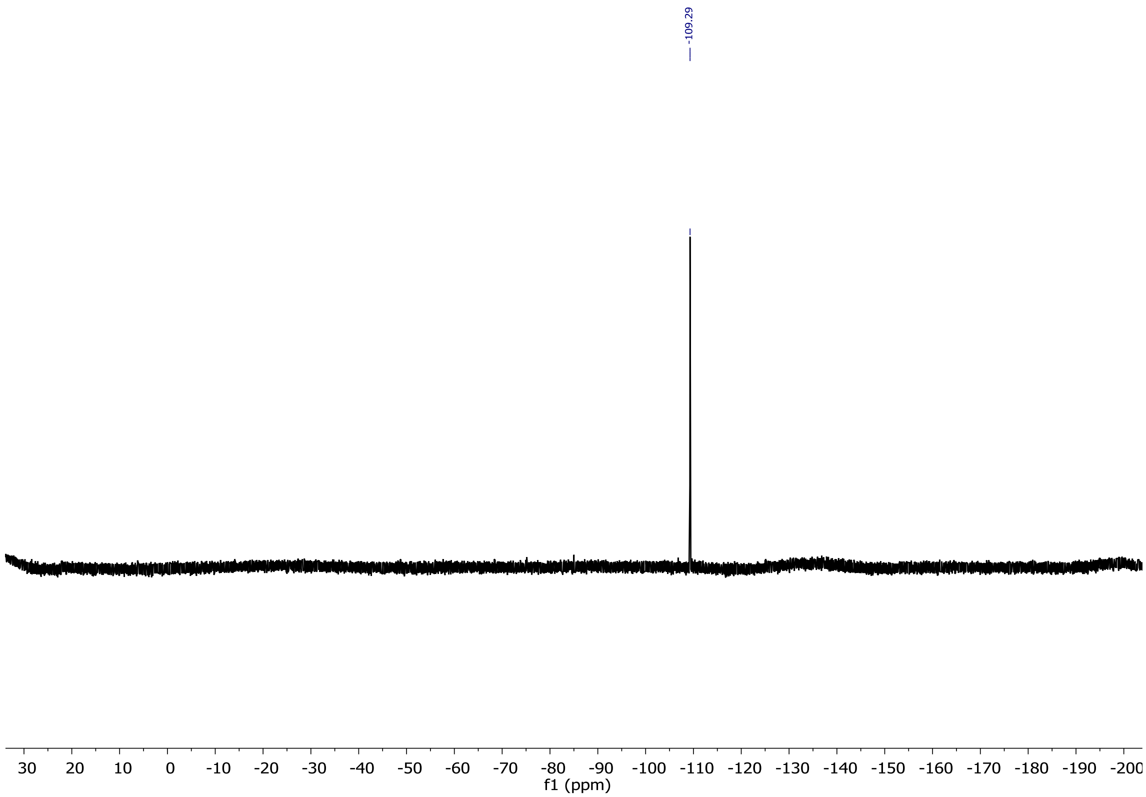

Supplementary Figure 42. ^{19}F NMR spectrum ($CDCl_3$) of compound S8.

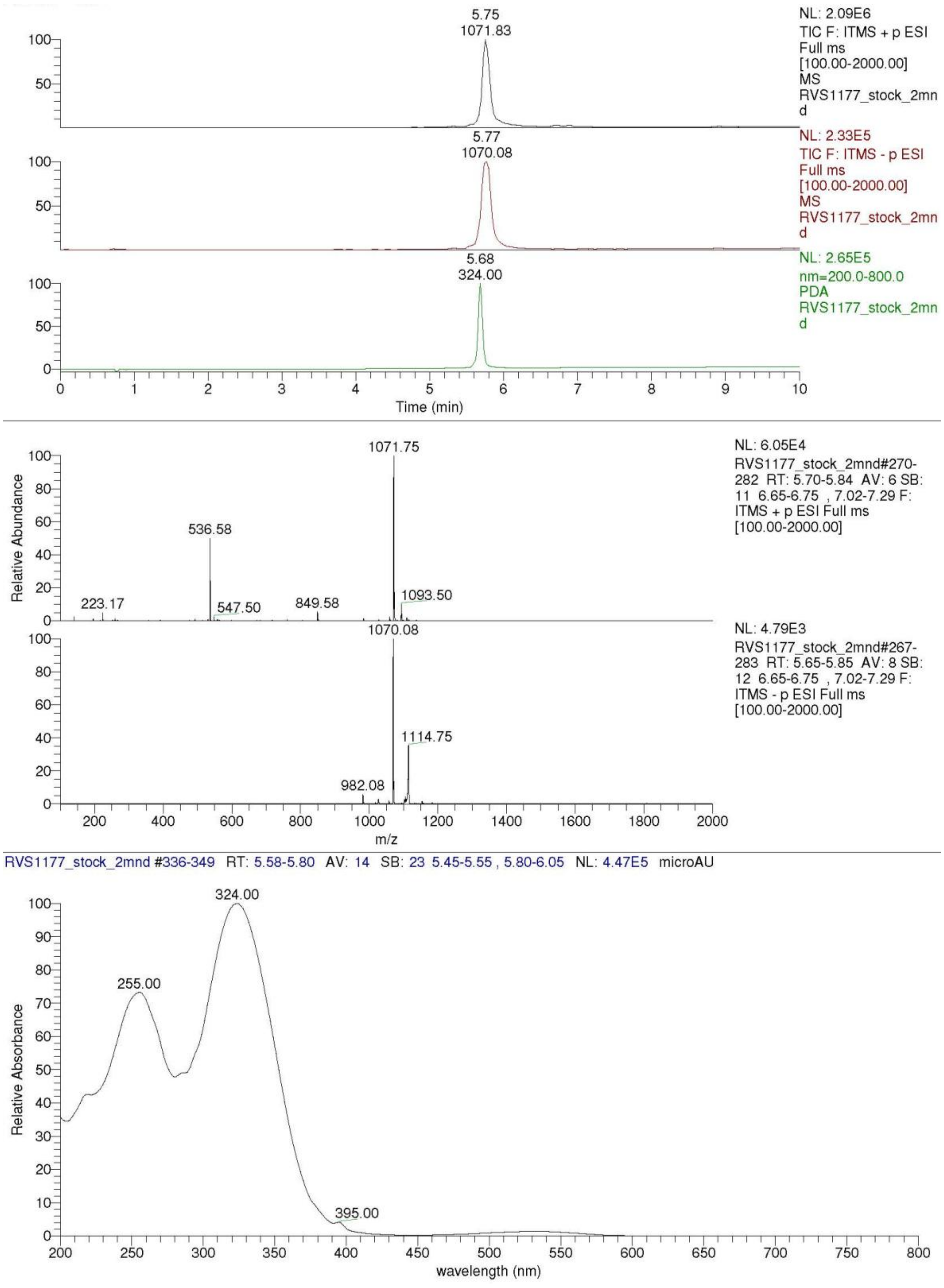

Supplementary Figure 43. HPLC-MS/PDA chromatogram with MS and UV spectra of compound S8.

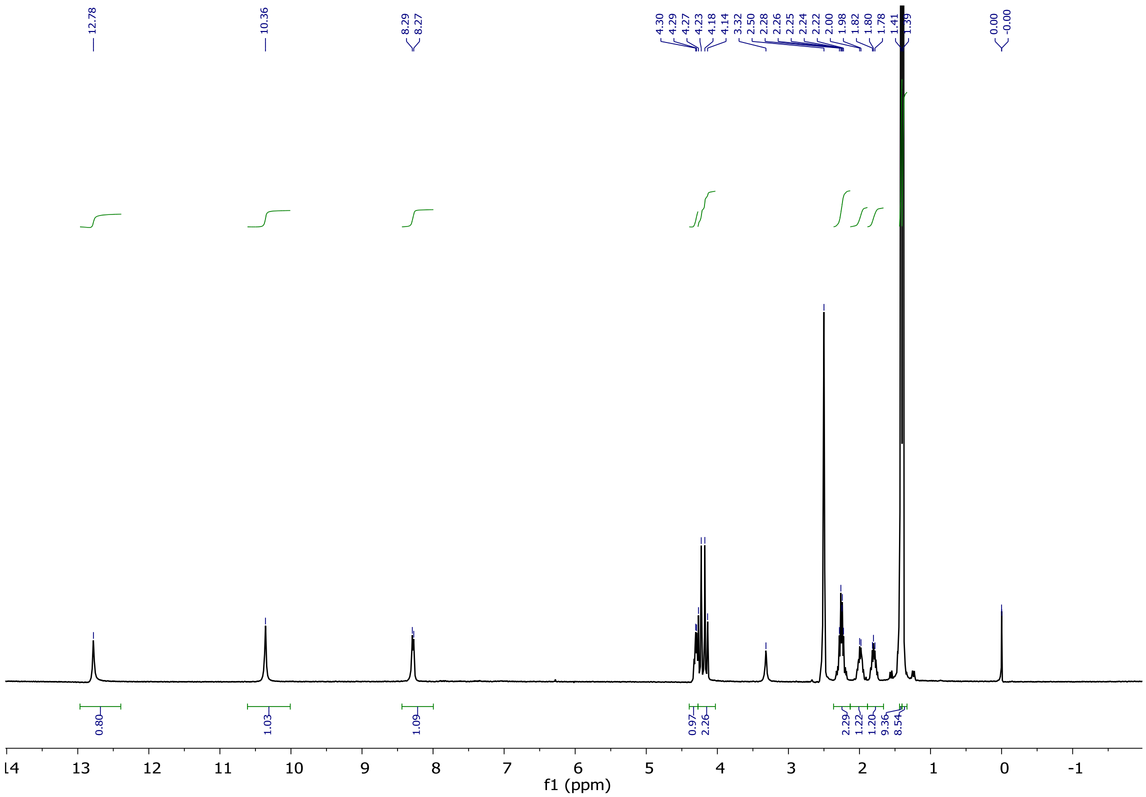

Supplementary Figure 44. ^{1}H NMR spectrum (DMSO-d6) of compound S9.

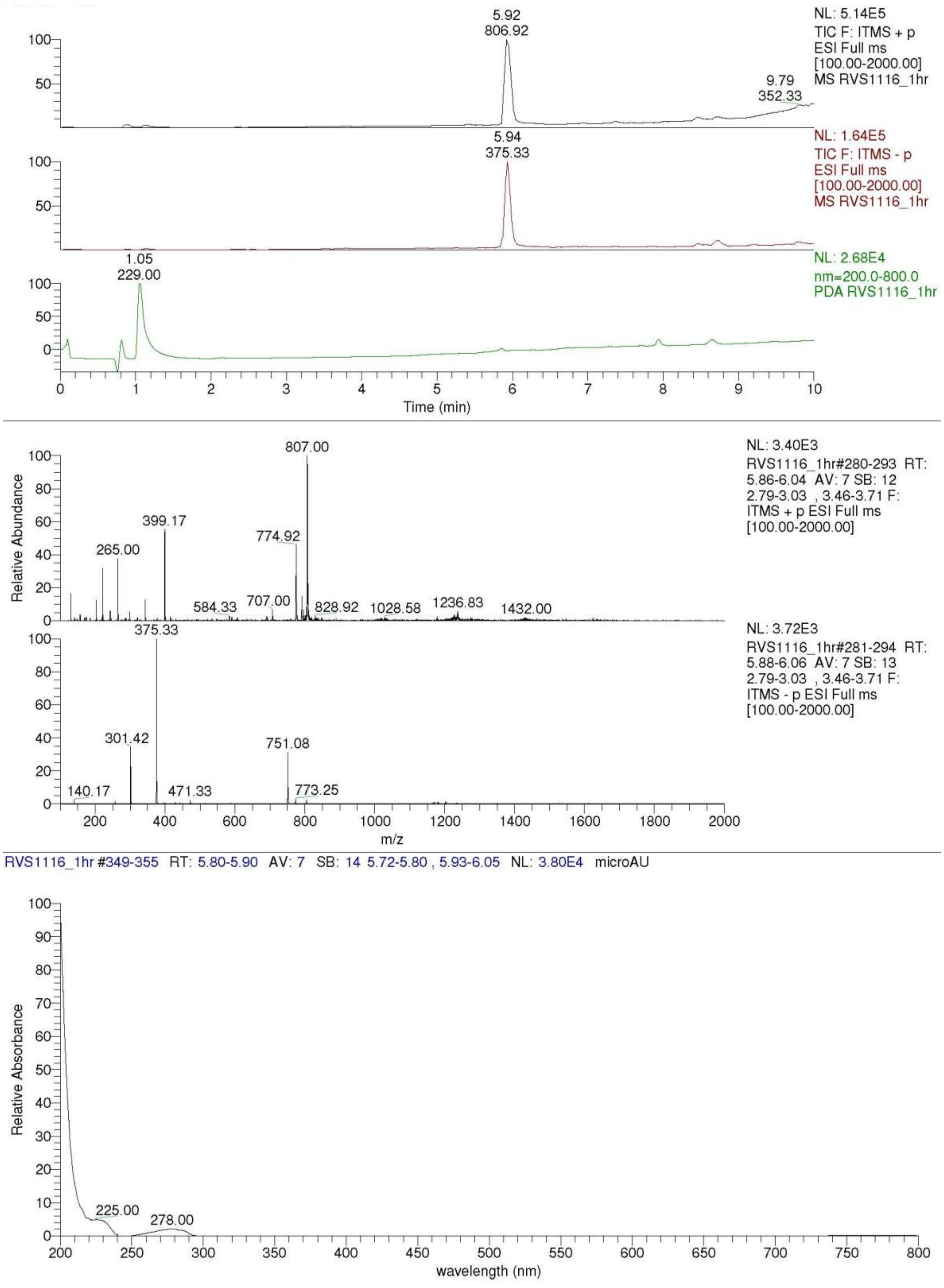

Supplementary Figure 45. HPLC-MS/PDA chromatogram with MS and UV spectra of compound S9.

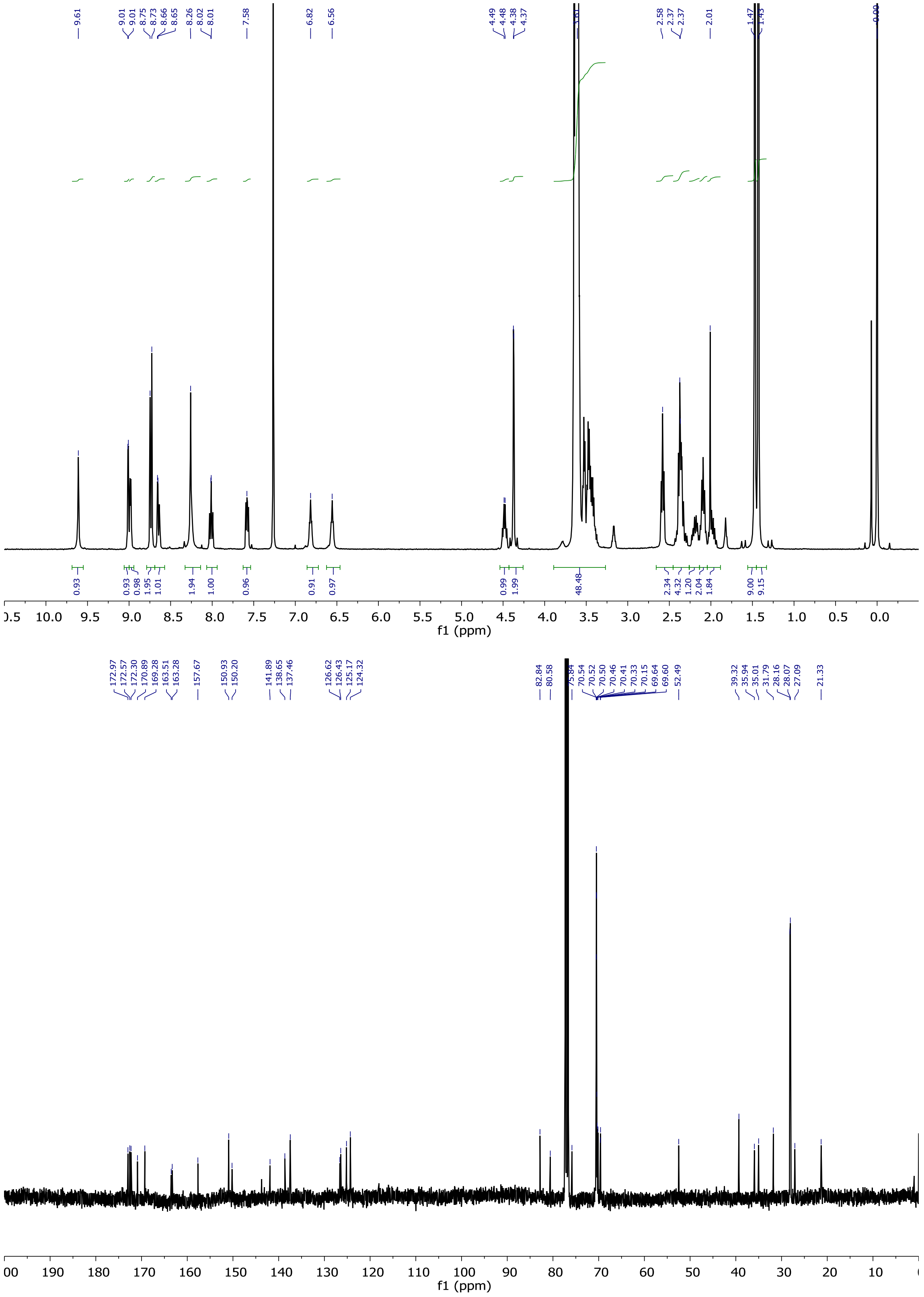

Supplementary Figure 46. (Top) ^{1}H and (bottom) ^{13}C NMR spectra ($CDCl_3$) of compound S10.

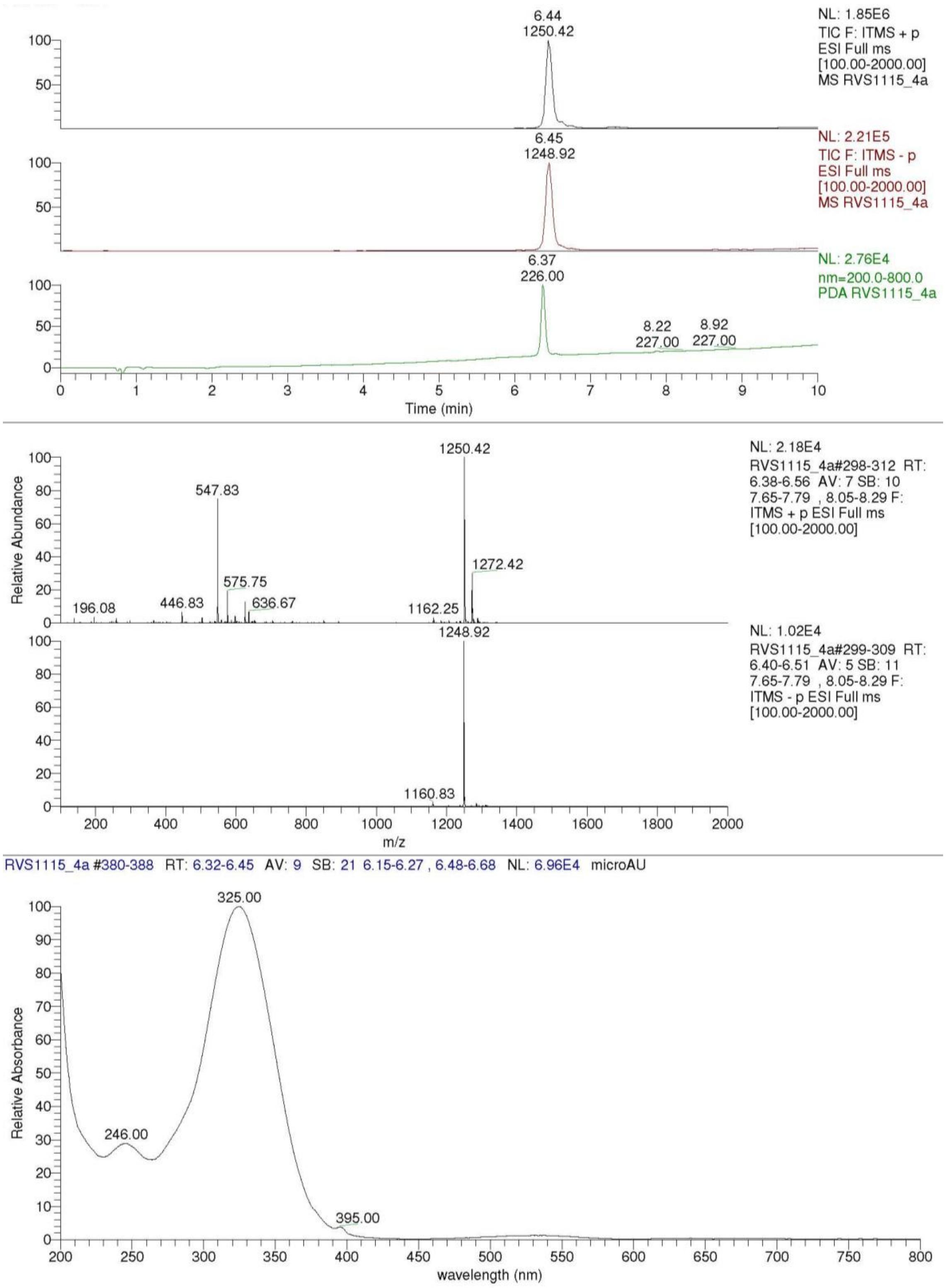

Supplementary Figure 47. HPLC-MS/PDA chromatogram with MS and UV spectra of compound S10.

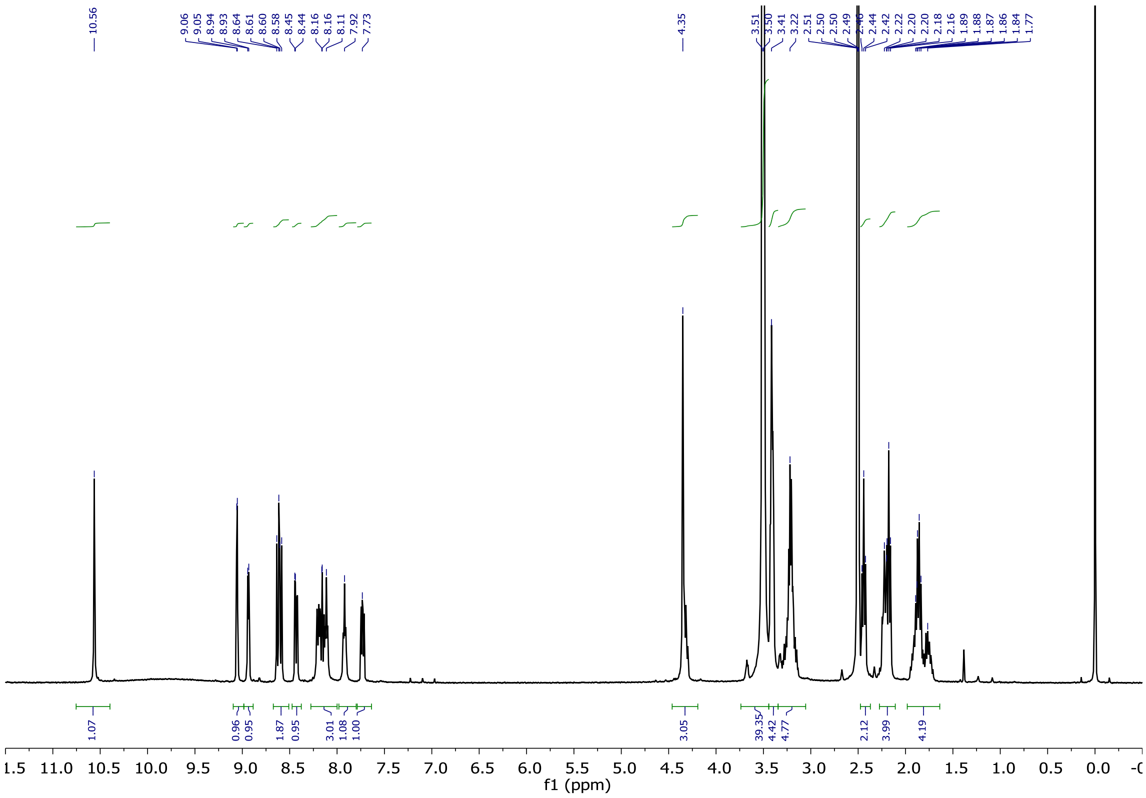

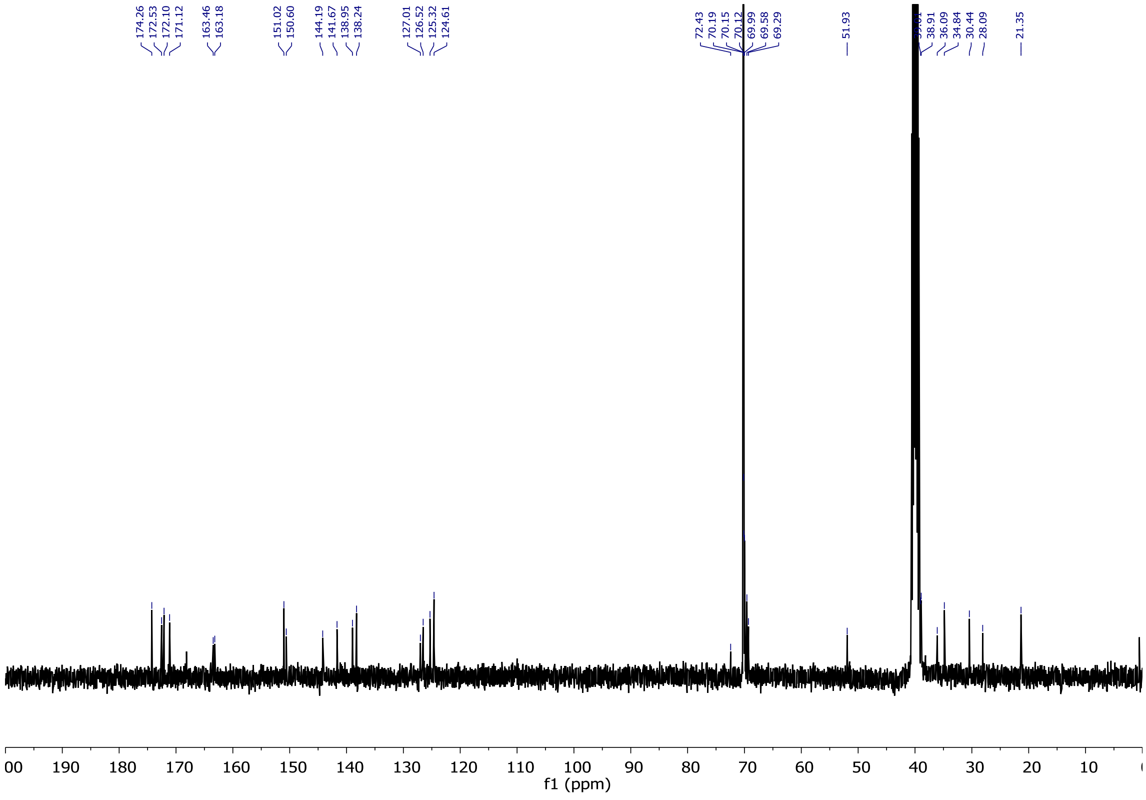

Supplementary Figure 48. (Top) ^{1}H and (bottom) ^{13}C NMR spectra (DMSO-d6) of compound S11.

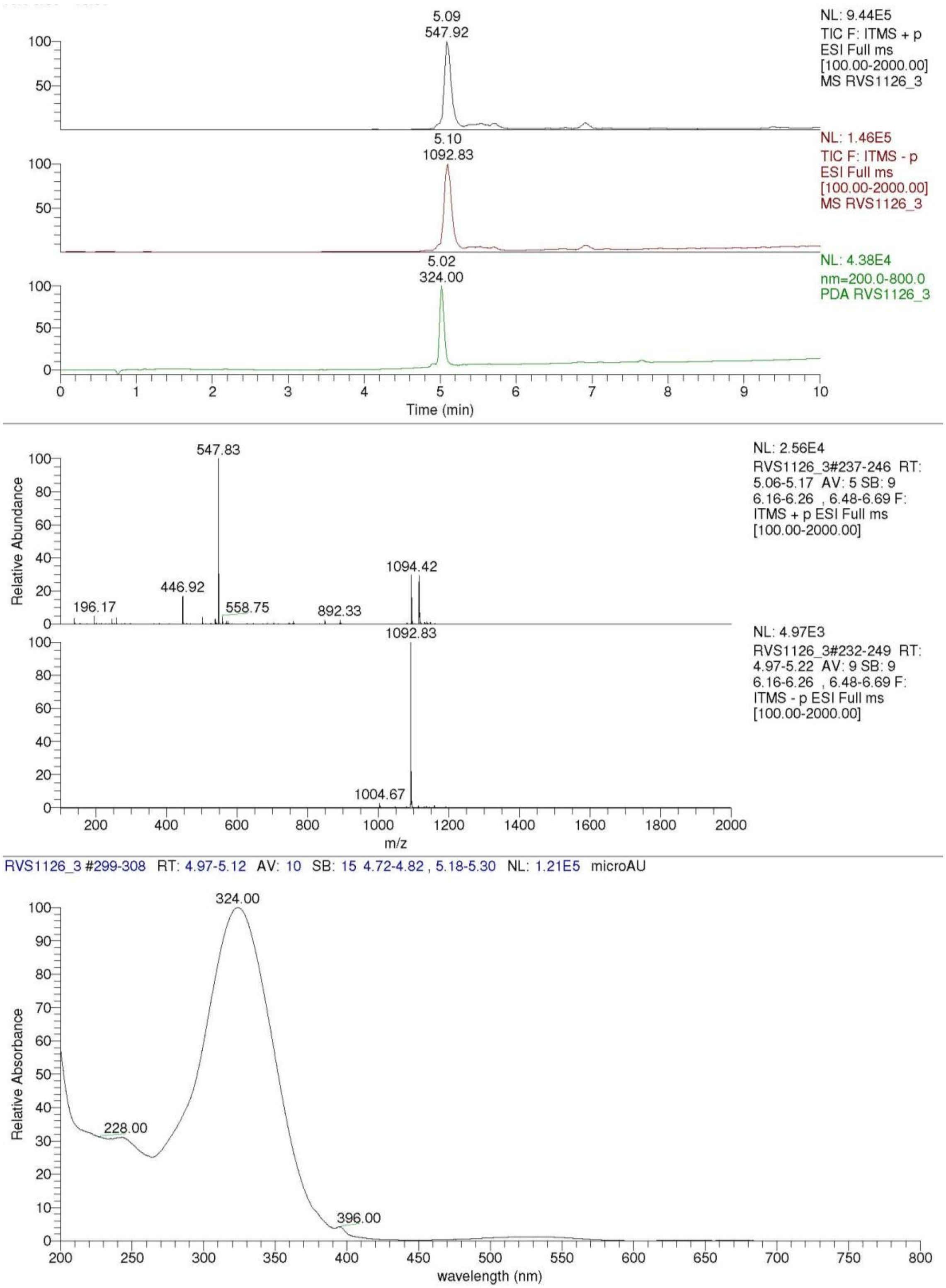

Supplementary Figure 49. HPLC-MS/PDA chromatogram with MS and UV spectra of compound S11.

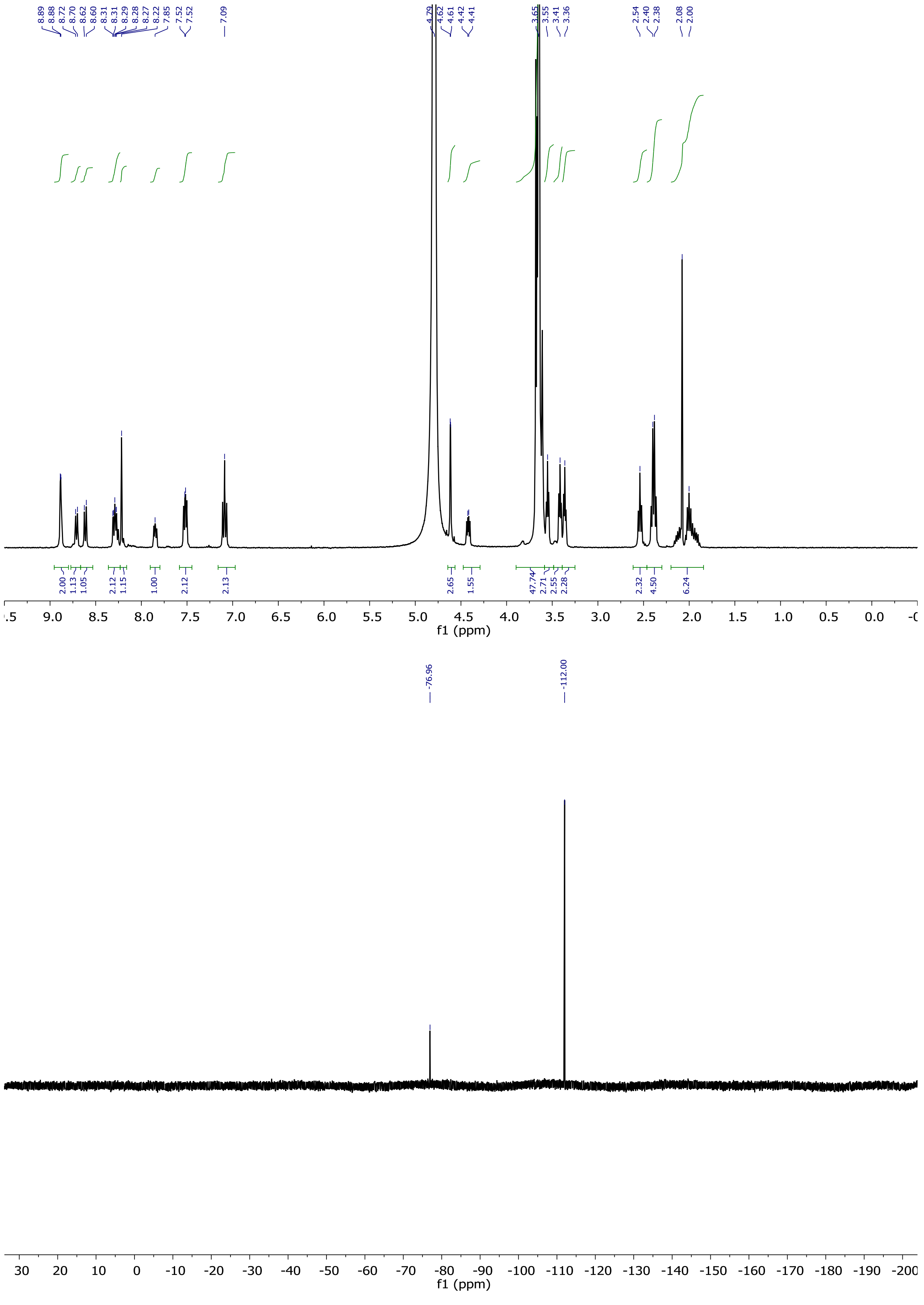

Supplementary Figure 50. (Top) ^{1}H and (bottom) ^{19}F NMR spectra (D_2O) of compound S12.

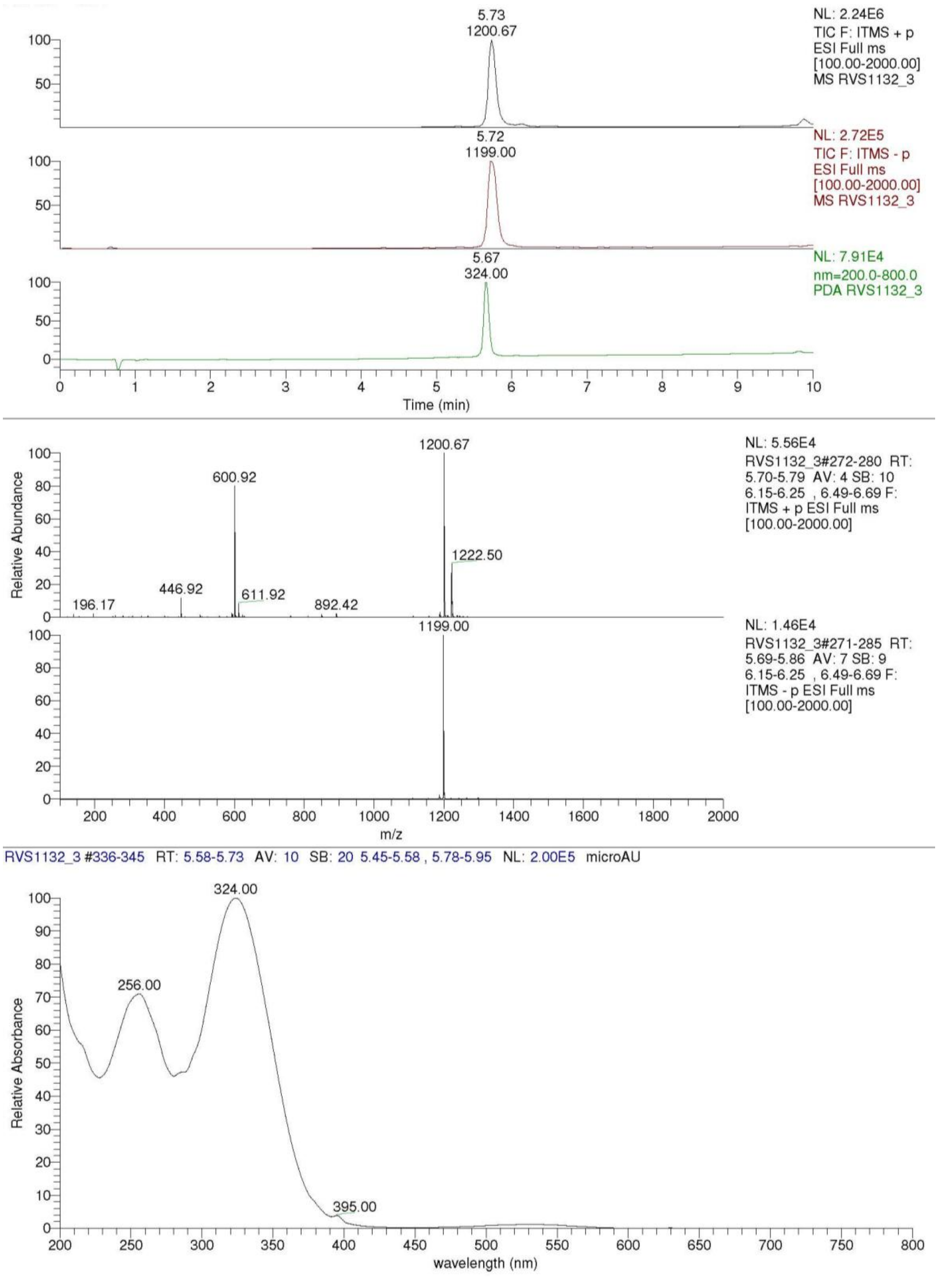

Supplementary Figure 51. HPLC-MS/PDA chromatogram with MS and UV spectra of compound S12.

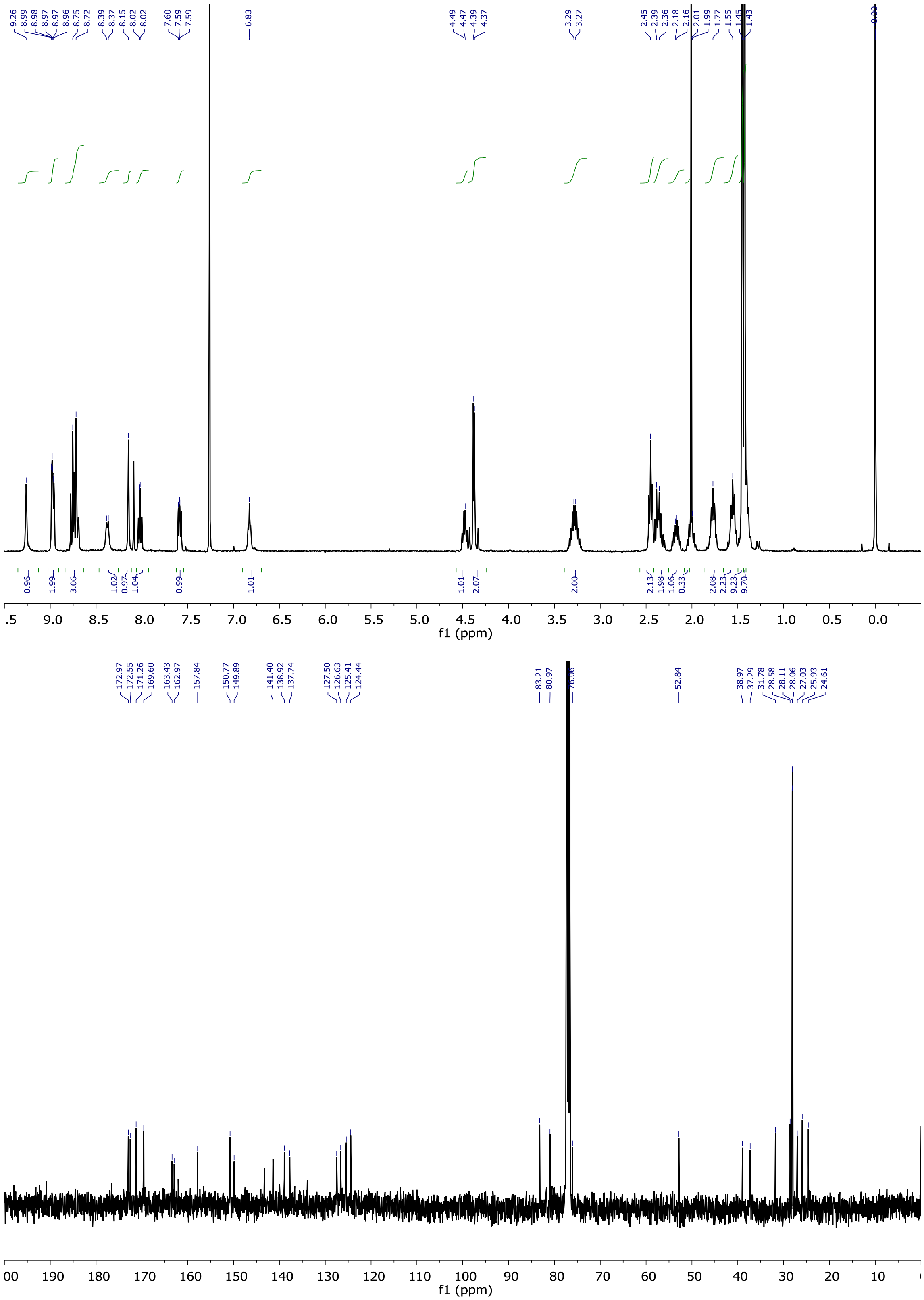

Supplementary Figure 52. (Top) ^{1}H and (bottom) ^{13}C NMR spectra ($CDCl_3$) of compound S17.

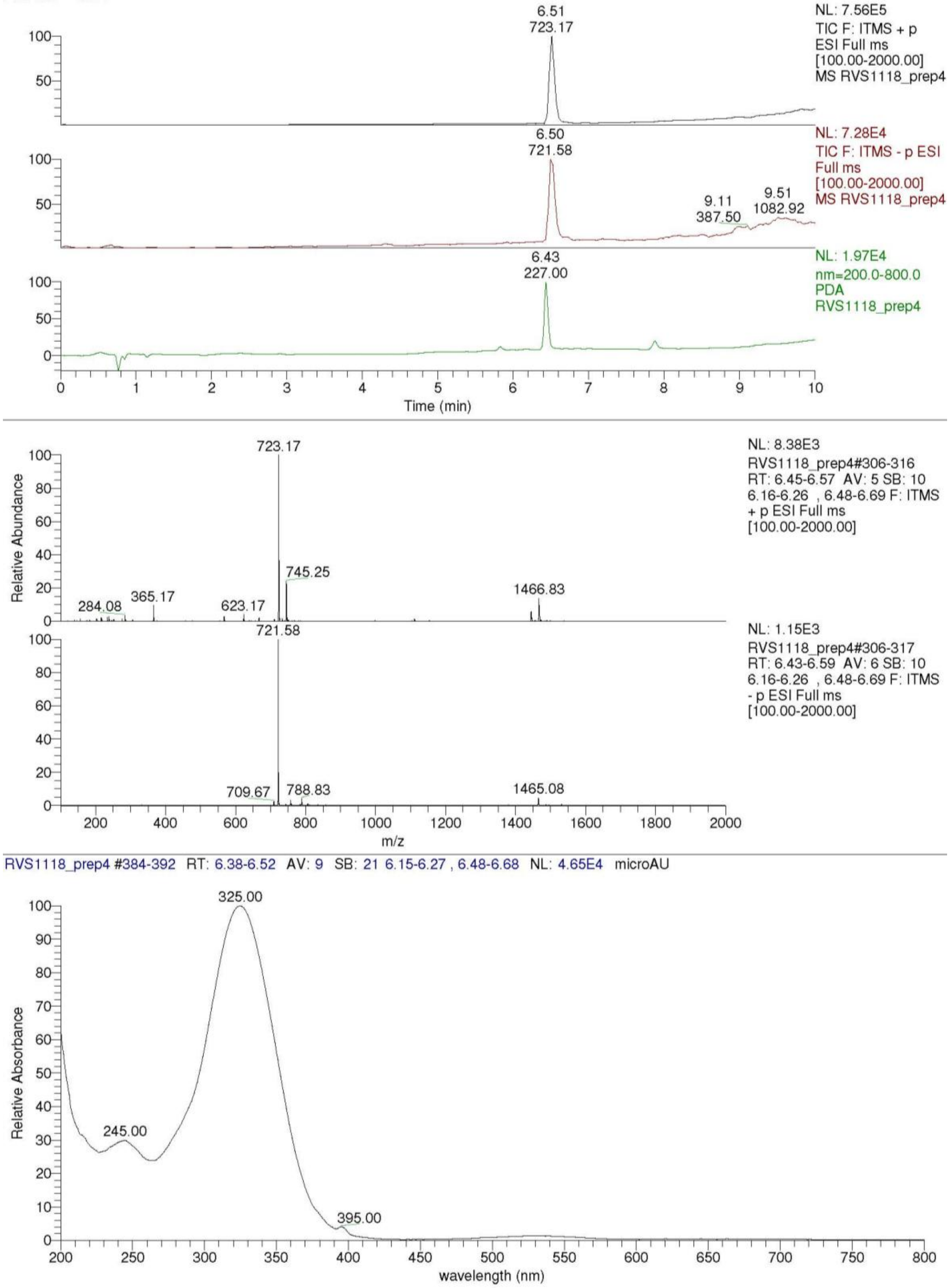

Supplementary Figure 53. HPLC-MS/PDA chromatogram with MS and UV spectra of compound S17.

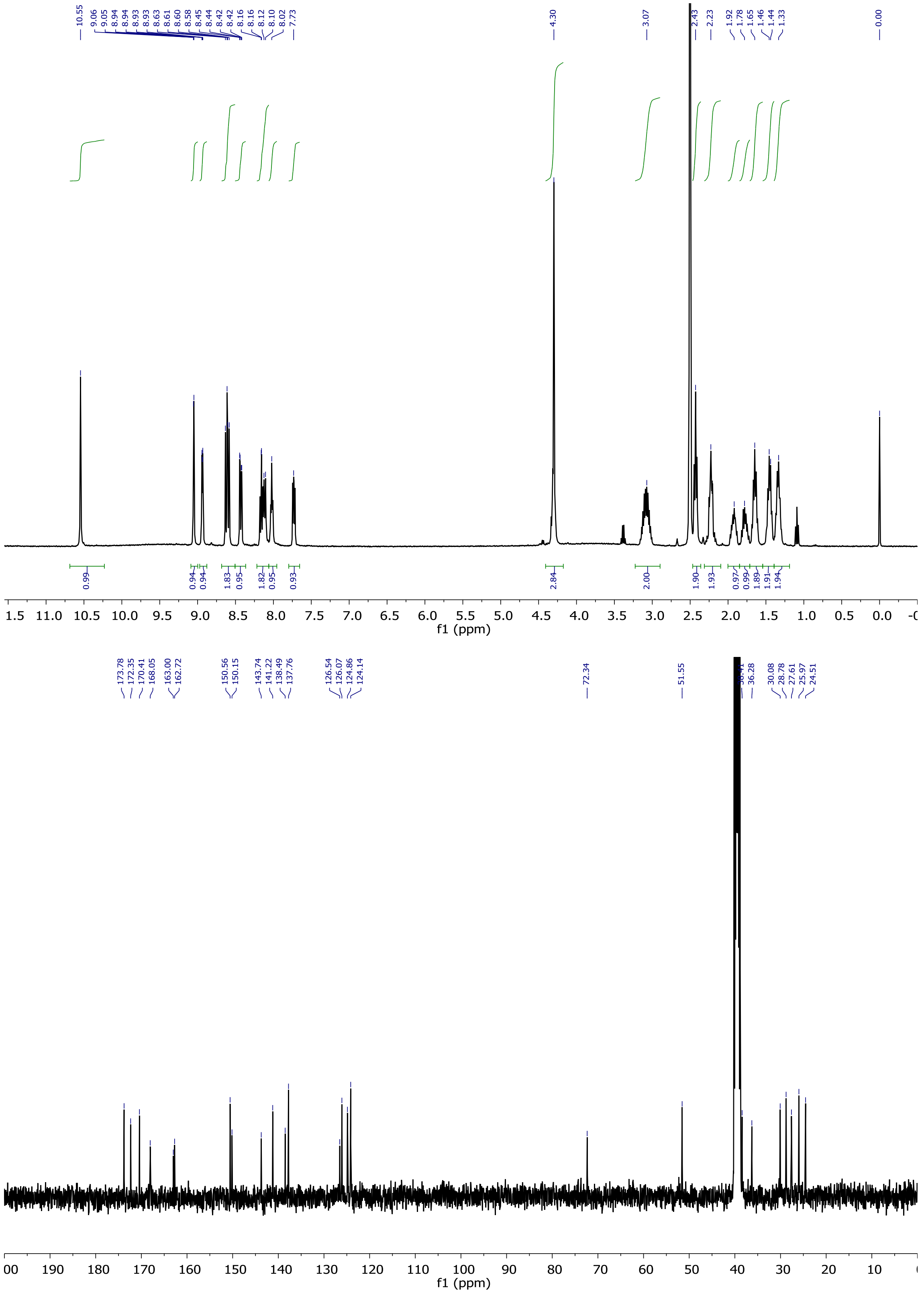

Supplementary Figure 54. (Top) ^{1}H and (bottom) ^{13}C NMR spectra (DMSO-d6) of compound S18.

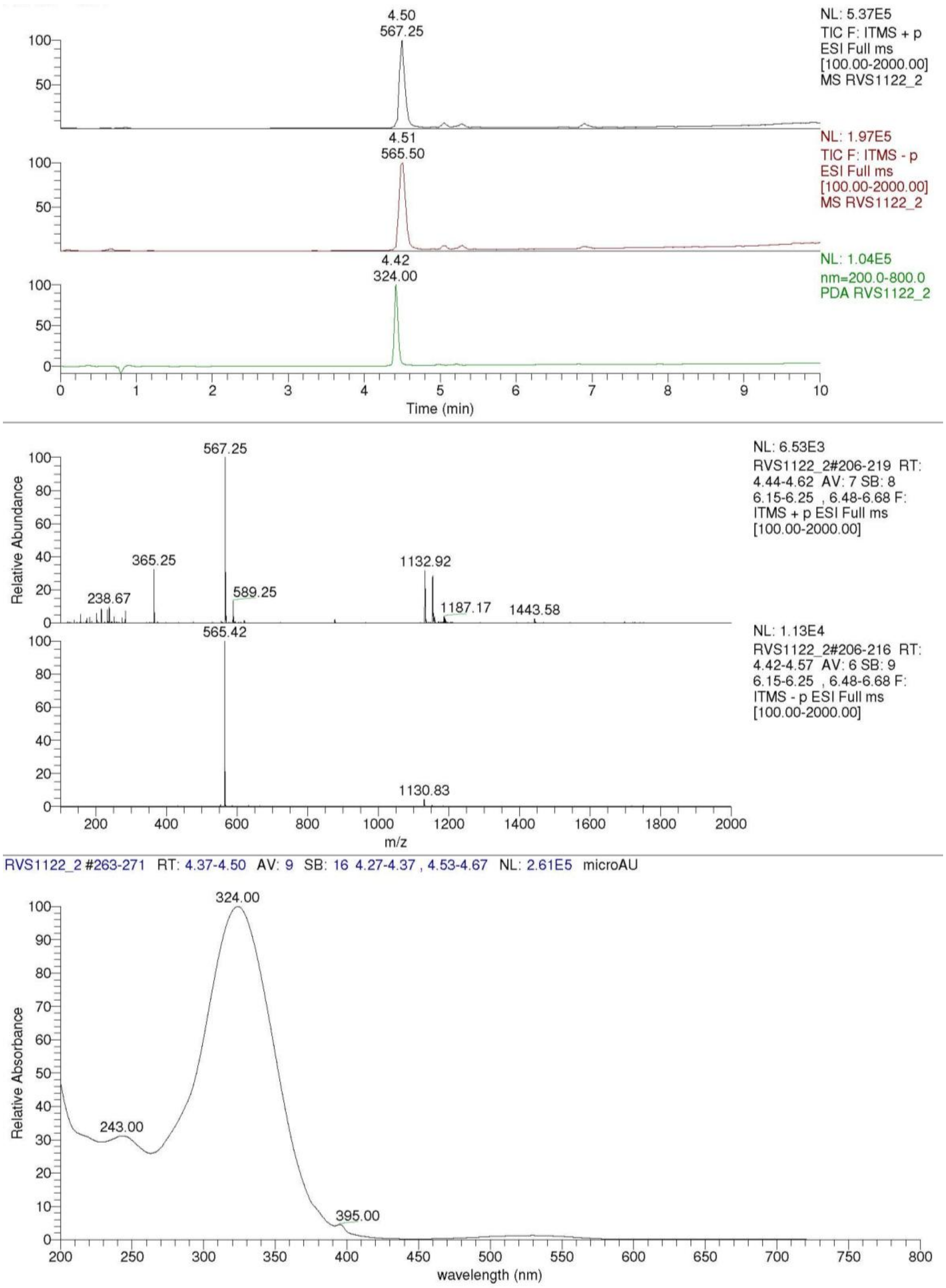

Supplementary Figure 55. HPLC-MS/PDA chromatogram with MS and UV spectra of compound S18.

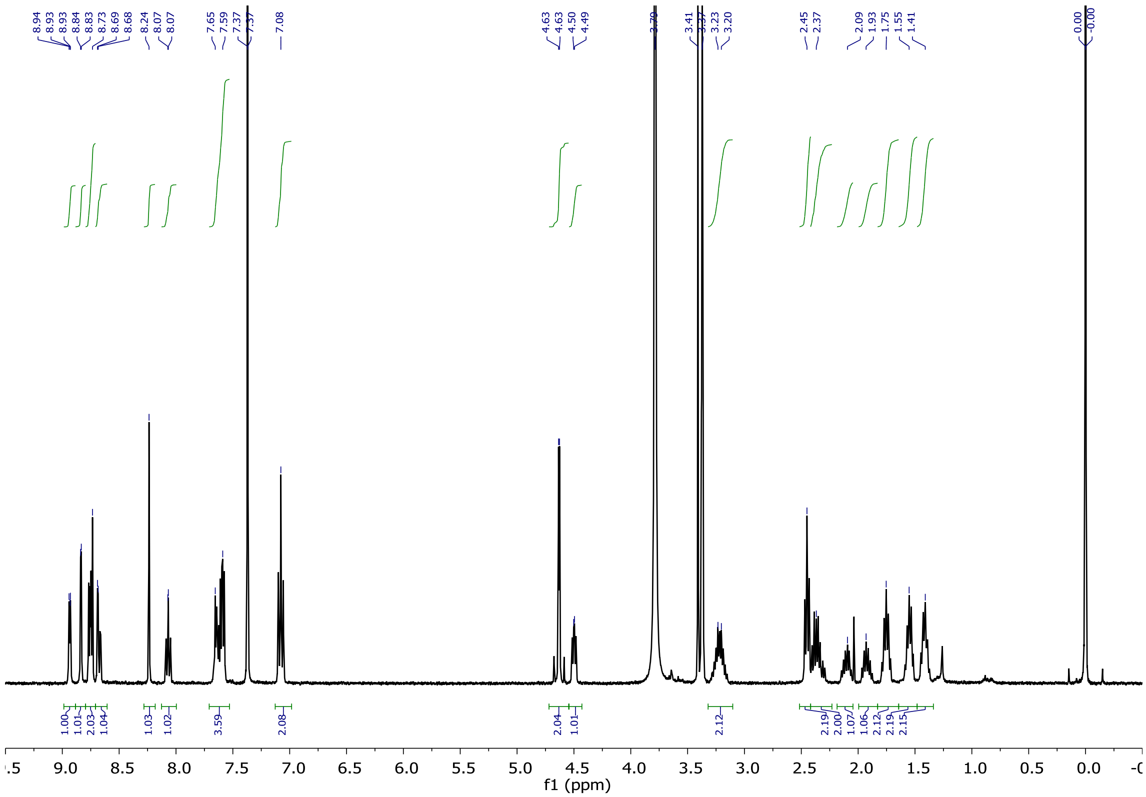

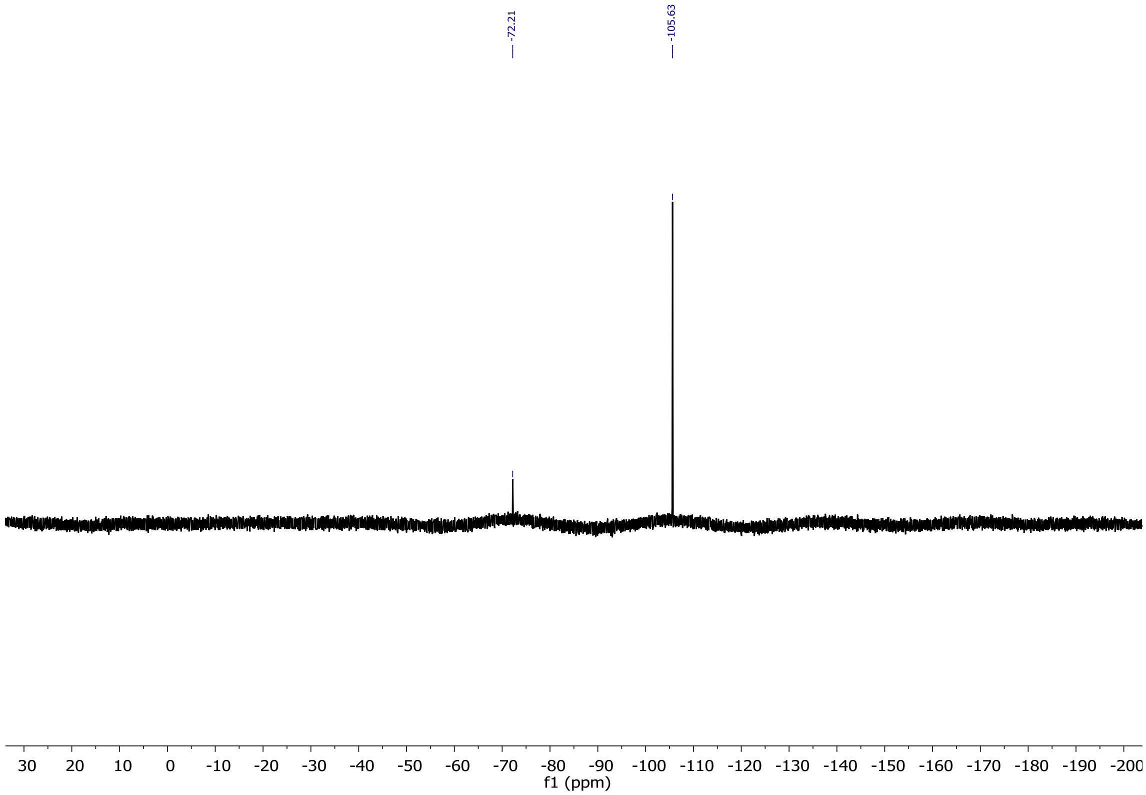

Supplementary Figure 56. (Top) ^{1}H and (bottom) ^{19}F NMR spectra ($CDCl_3$/MeOD-d4=5:1) of compound S19.

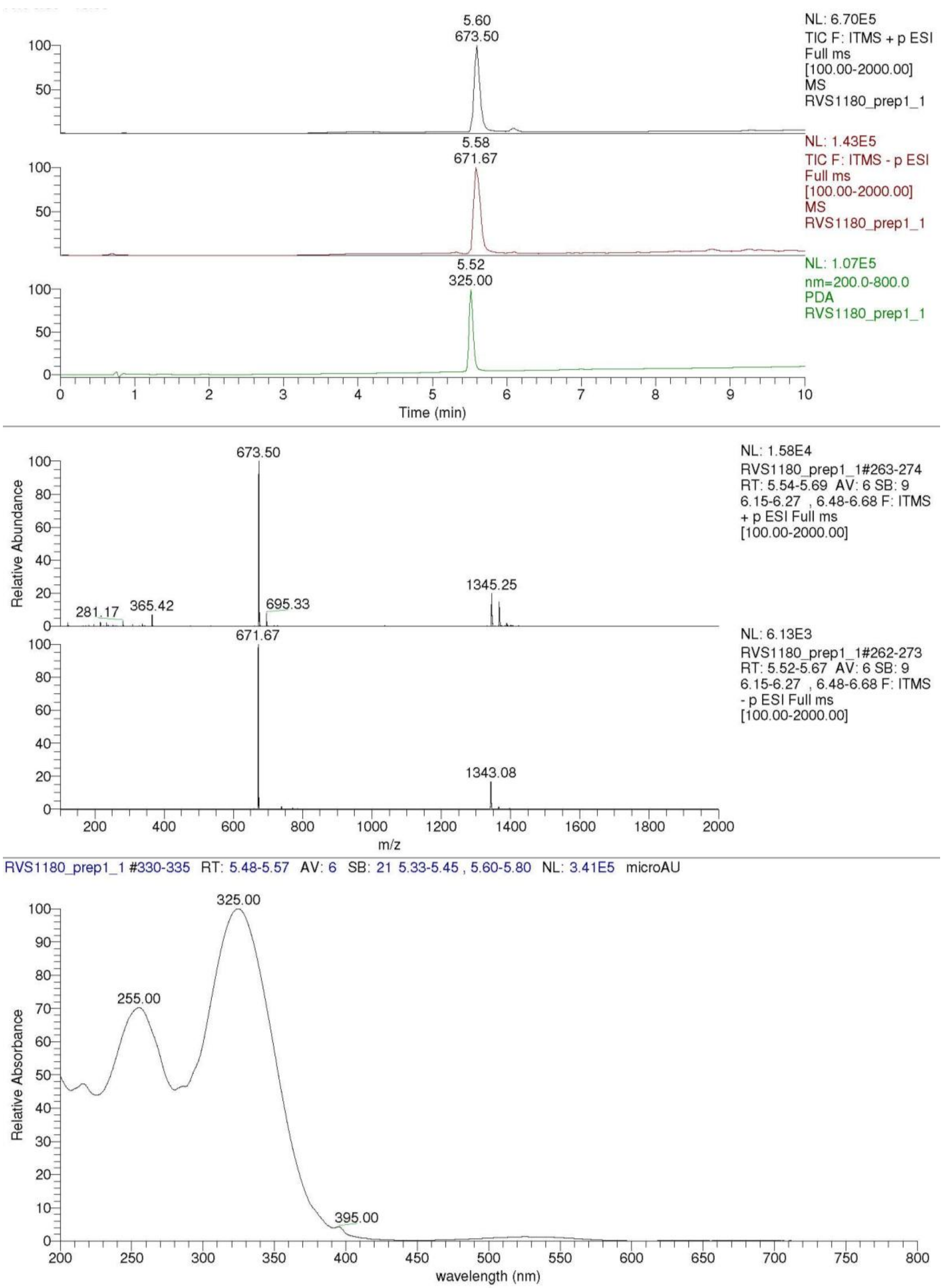

Supplementary Figure 57. HPLC-MS/PDA chromatogram with MS and UV spectra of compound S19.

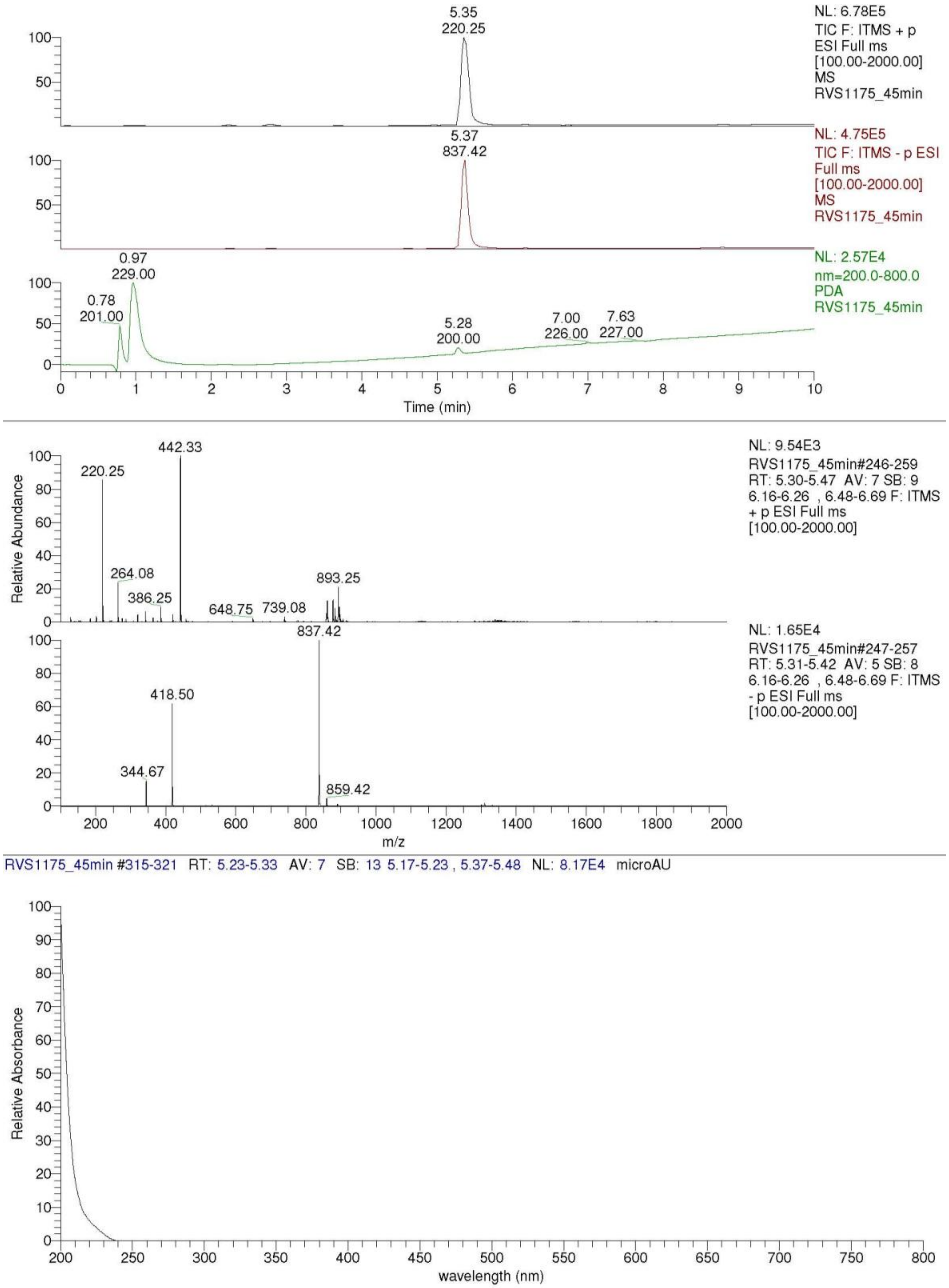

Supplementary Figure 58. HPLC-MS/PDA chromatogram with MS and UV spectra of compound S20.

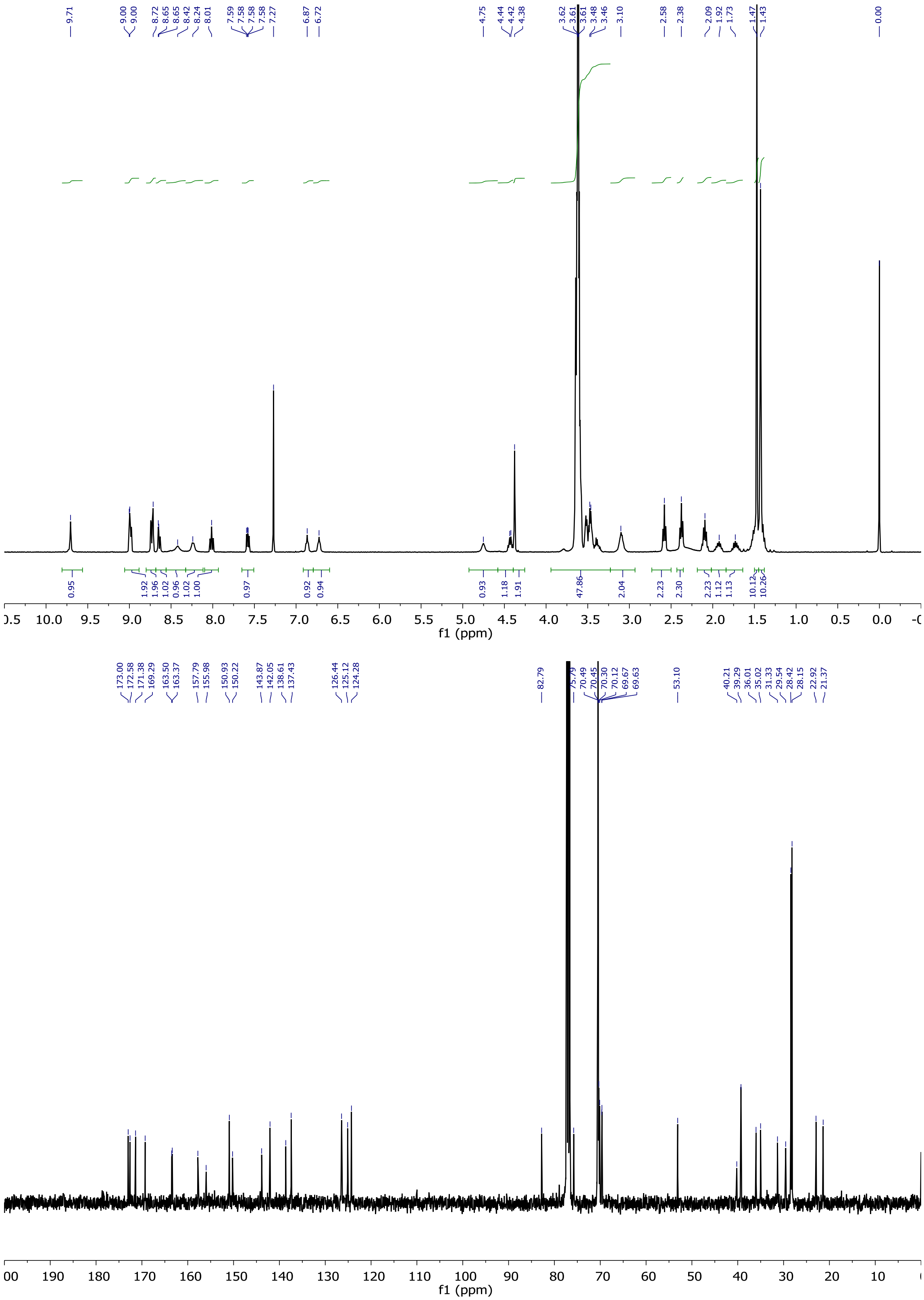

Supplementary Figure 59. (Top) ^{1}H and (bottom) ^{13}C NMR spectra ($CDCl_3$) of compound S21.

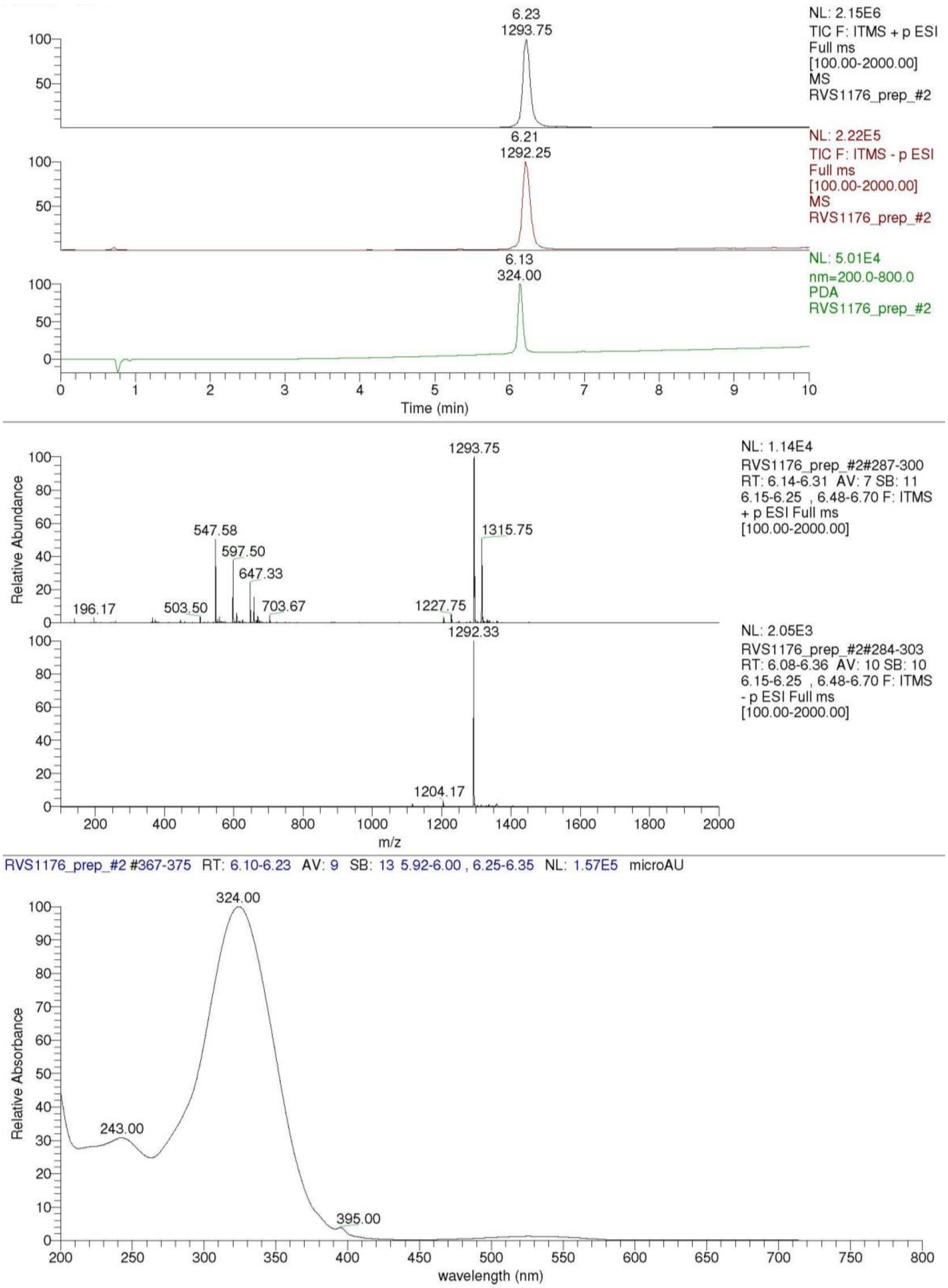

Supplementary Figure 60. HPLC-MS/PDA chromatogram with MS and UV spectra of compound S21.

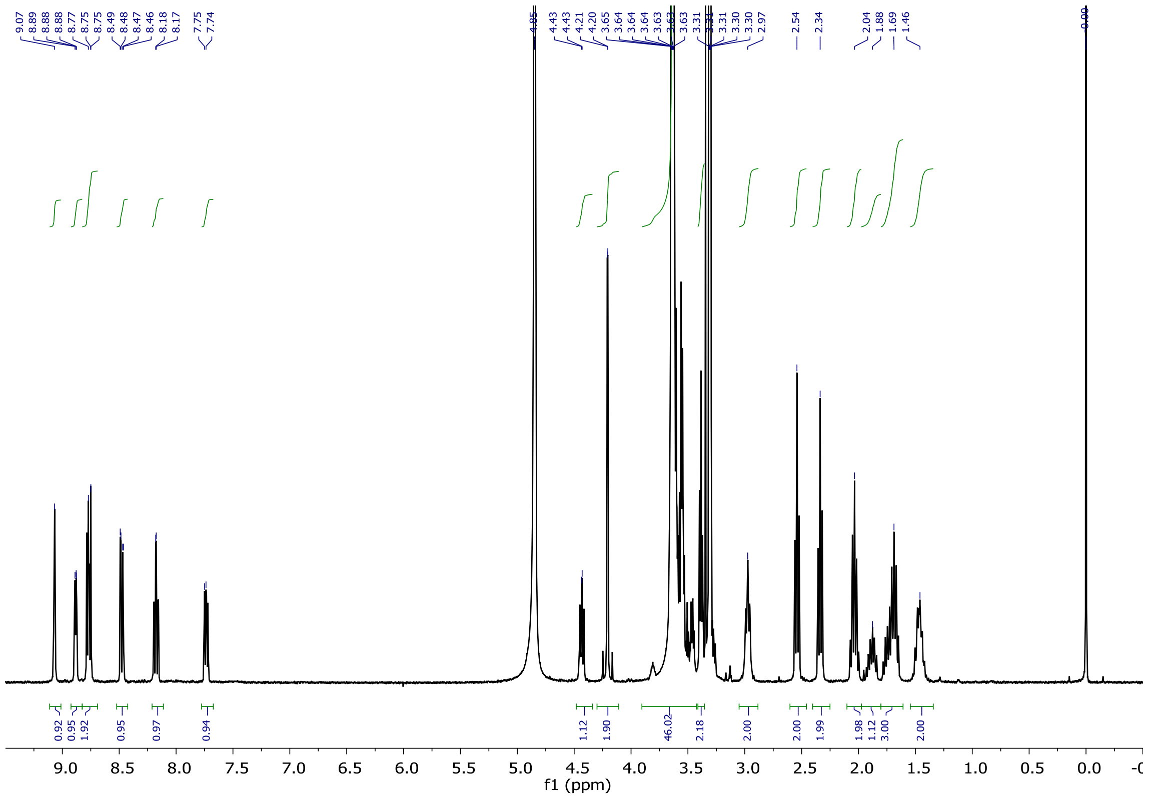

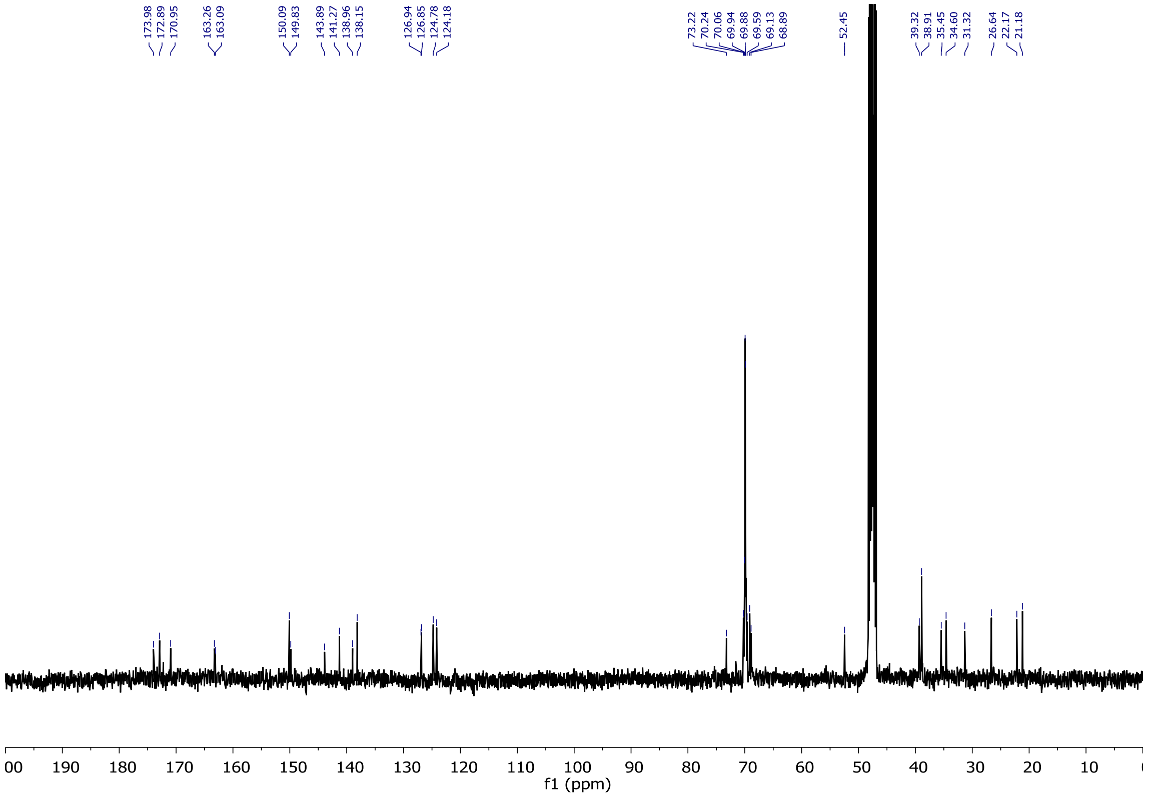

Supplementary Figure 61. (Top) ^{1}H and (bottom) ^{13}C NMR spectra (MeOD-d4) of compound S22.

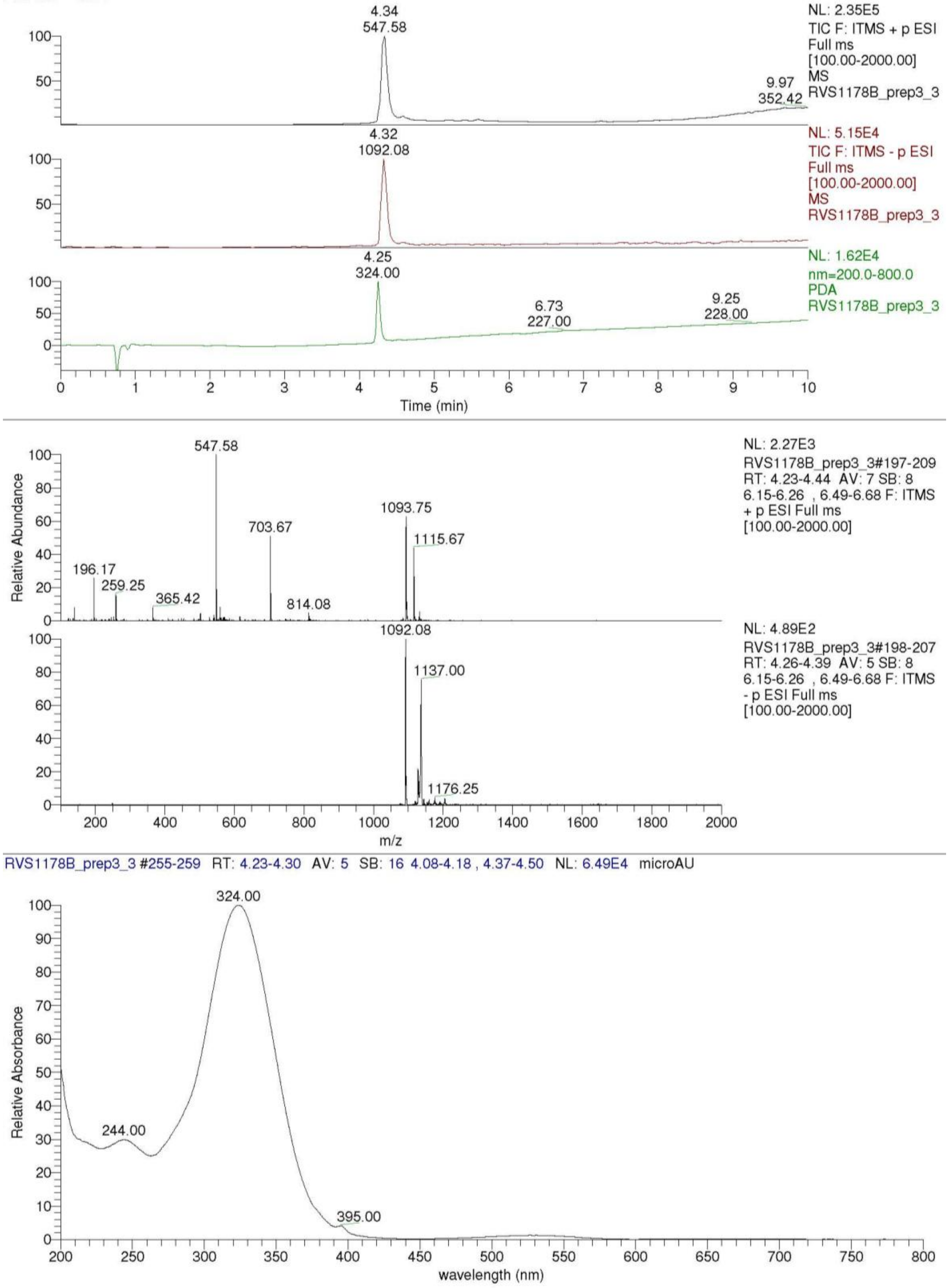

Supplementary Figure 62. HPLC-MS/PDA chromatogram with MS and UV spectra of compound S22.

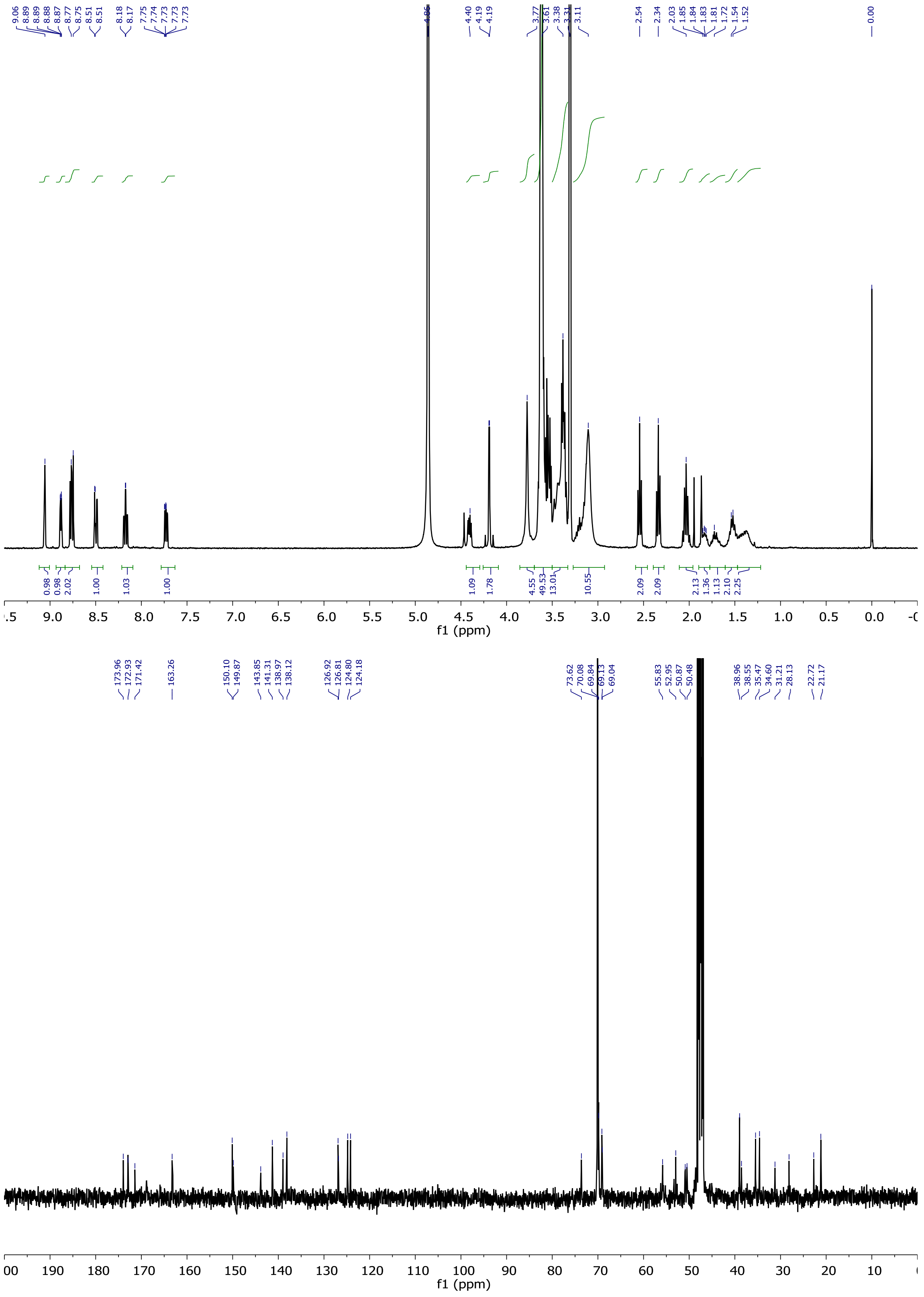

Supplementary Figure 63. (Top) ^{1}H and (bottom) ^{13}C NMR spectra (MeOD-d4) of compound S23.

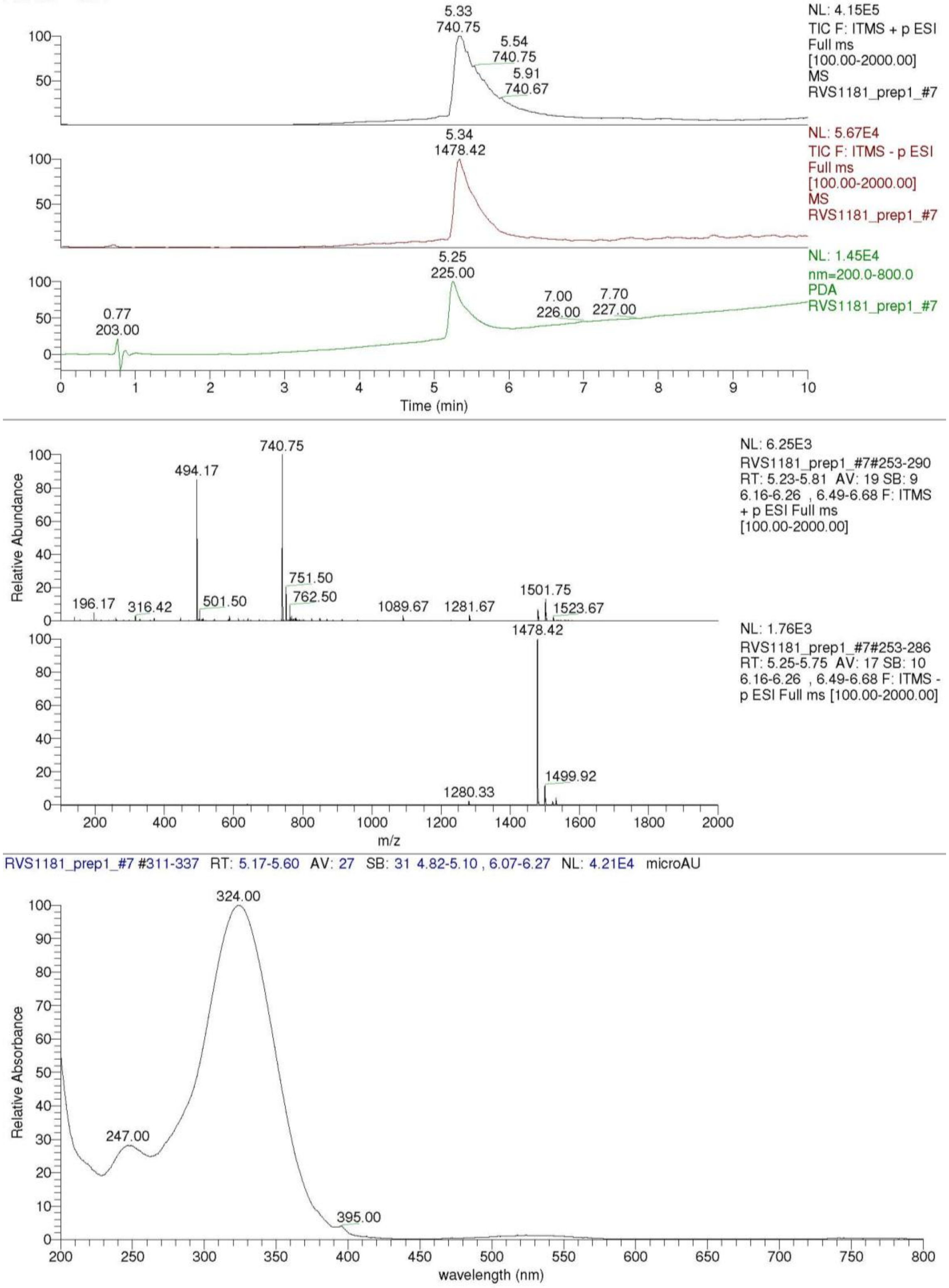

Supplementary Figure 64. HPLC-MS/PDA chromatogram with MS and UV spectra of compound S23.

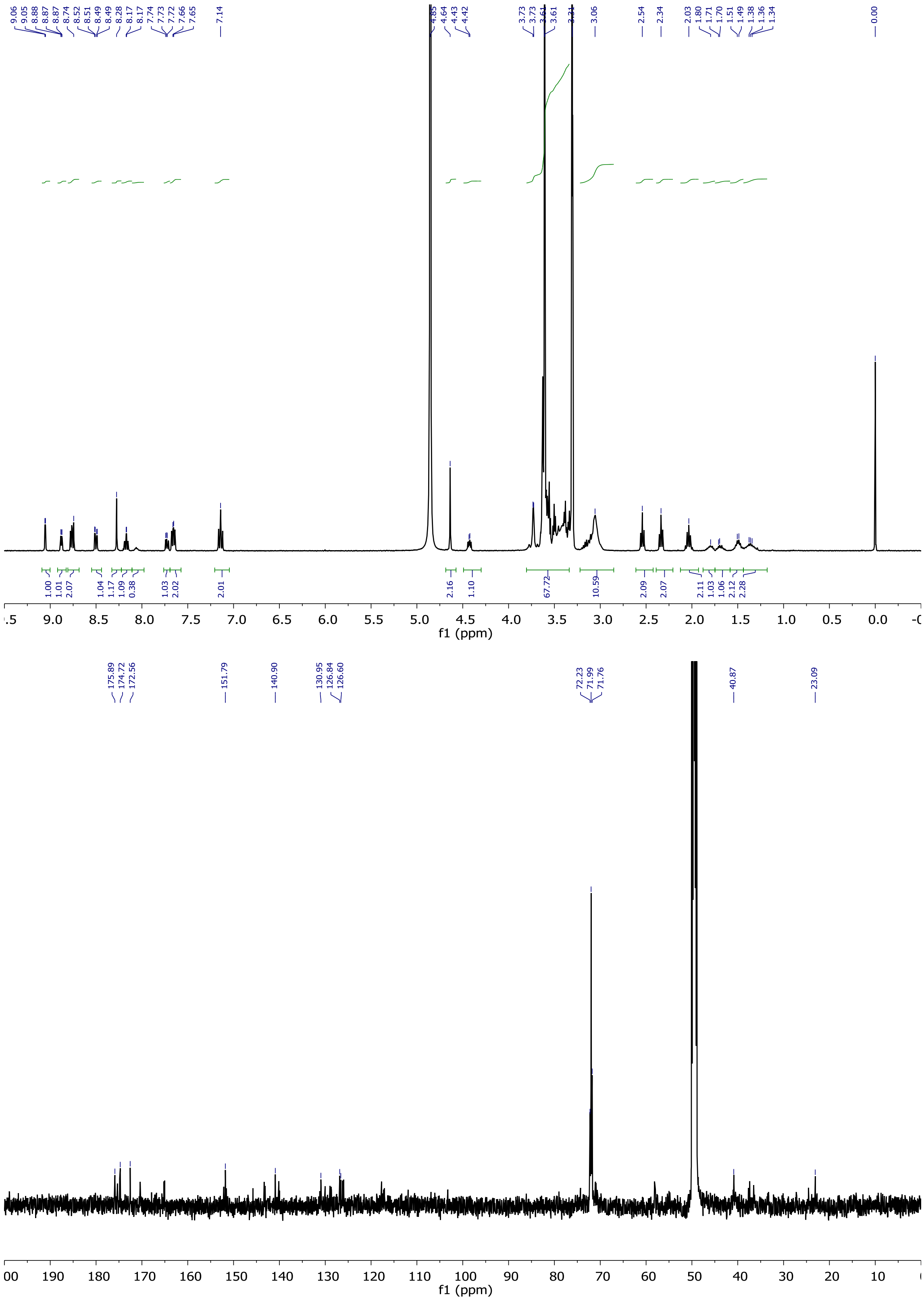

Supplementary Figure 65. (Top) ^{1}H and (bottom) ^{13}C NMR spectra (MeOD-d4) of compound S24.

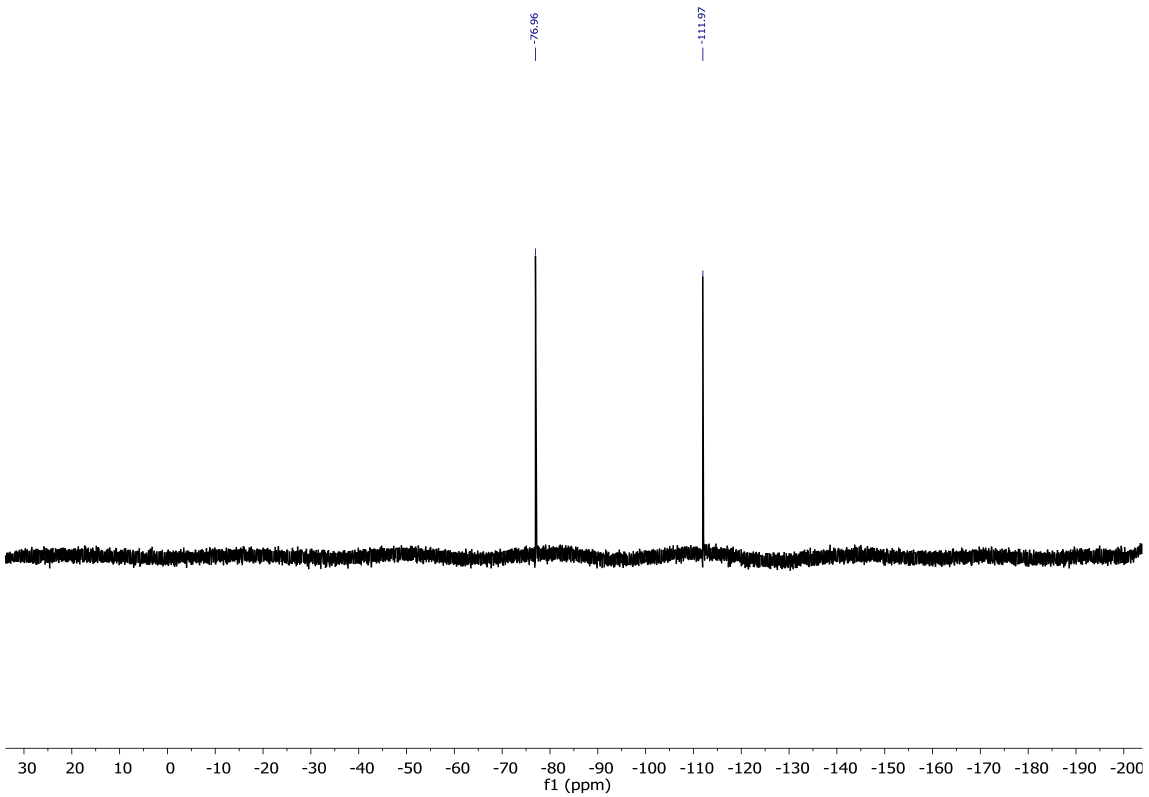

Supplementary Figure 66. ^{19}F NMR spectrum (MeOD-d4) of compound S24.

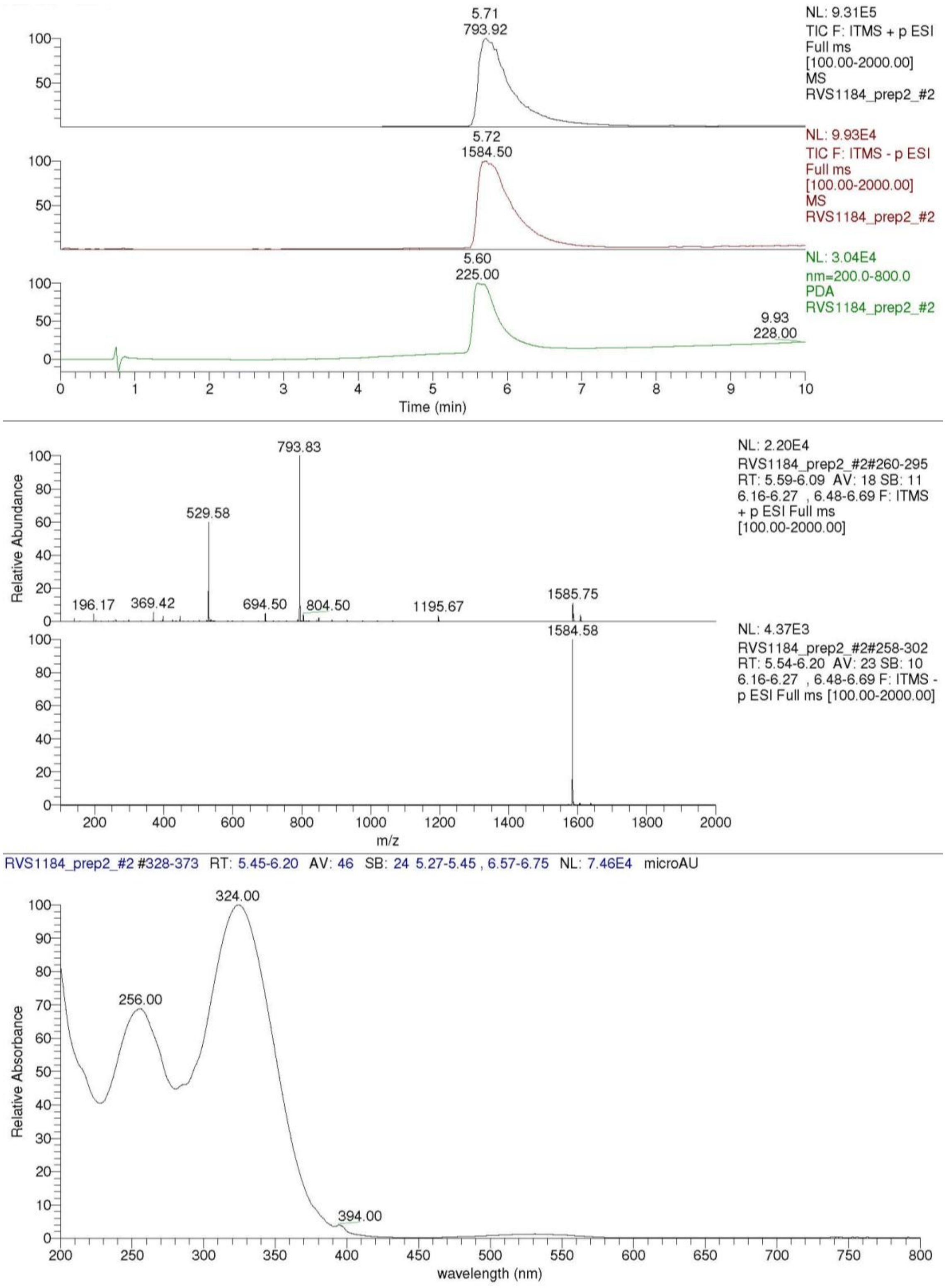

Supplementary Figure 67. HPLC-MS/PDA chromatogram with MS and UV spectra of compound S24.

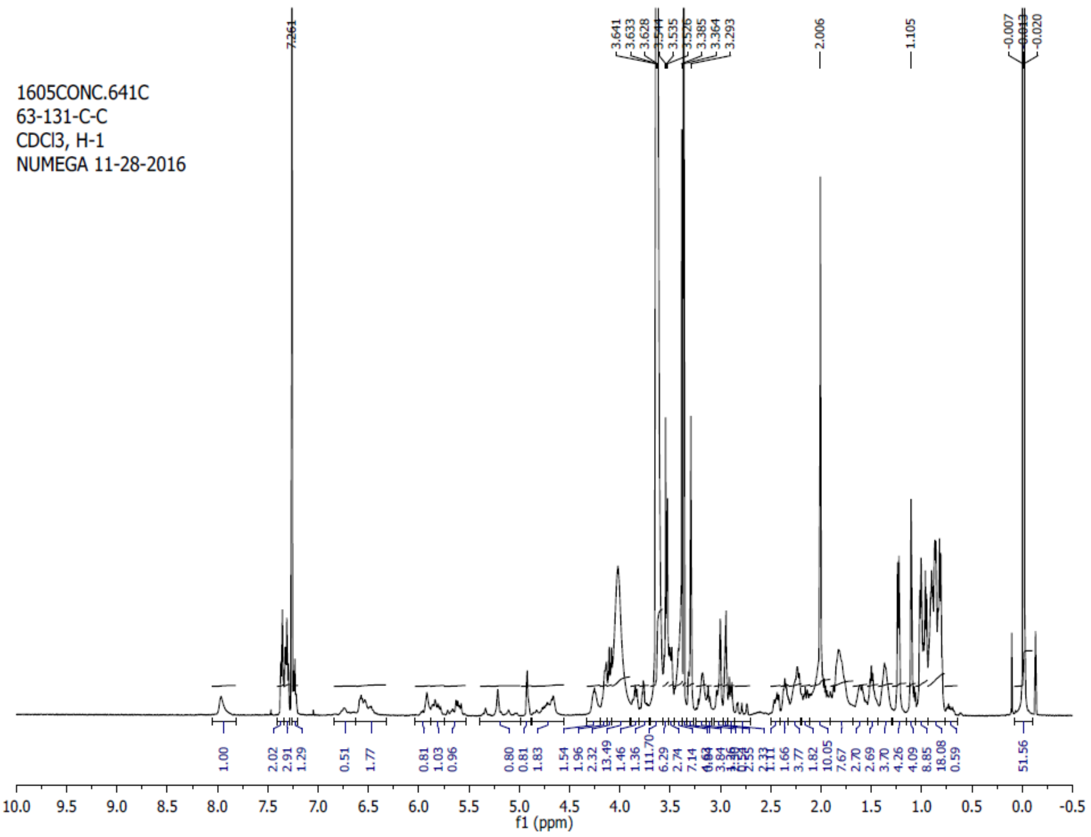

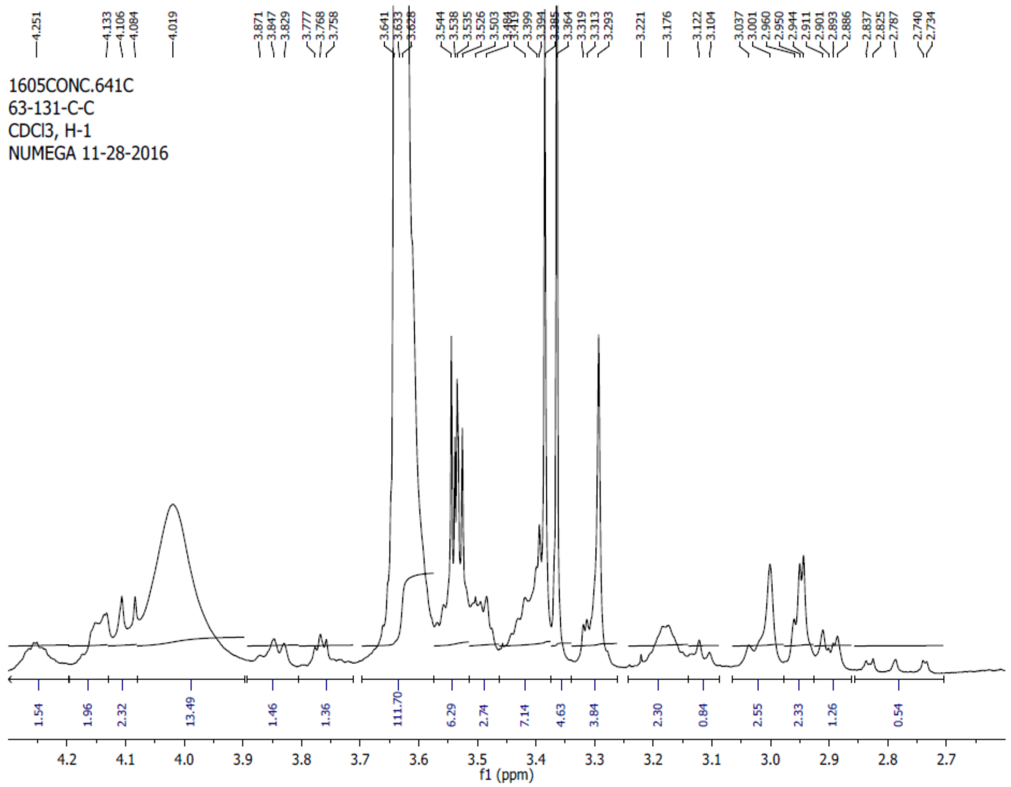

Supplementary Figure 68. ^{1}H NMR spectrum ($CDCl_3$) of compound S27.

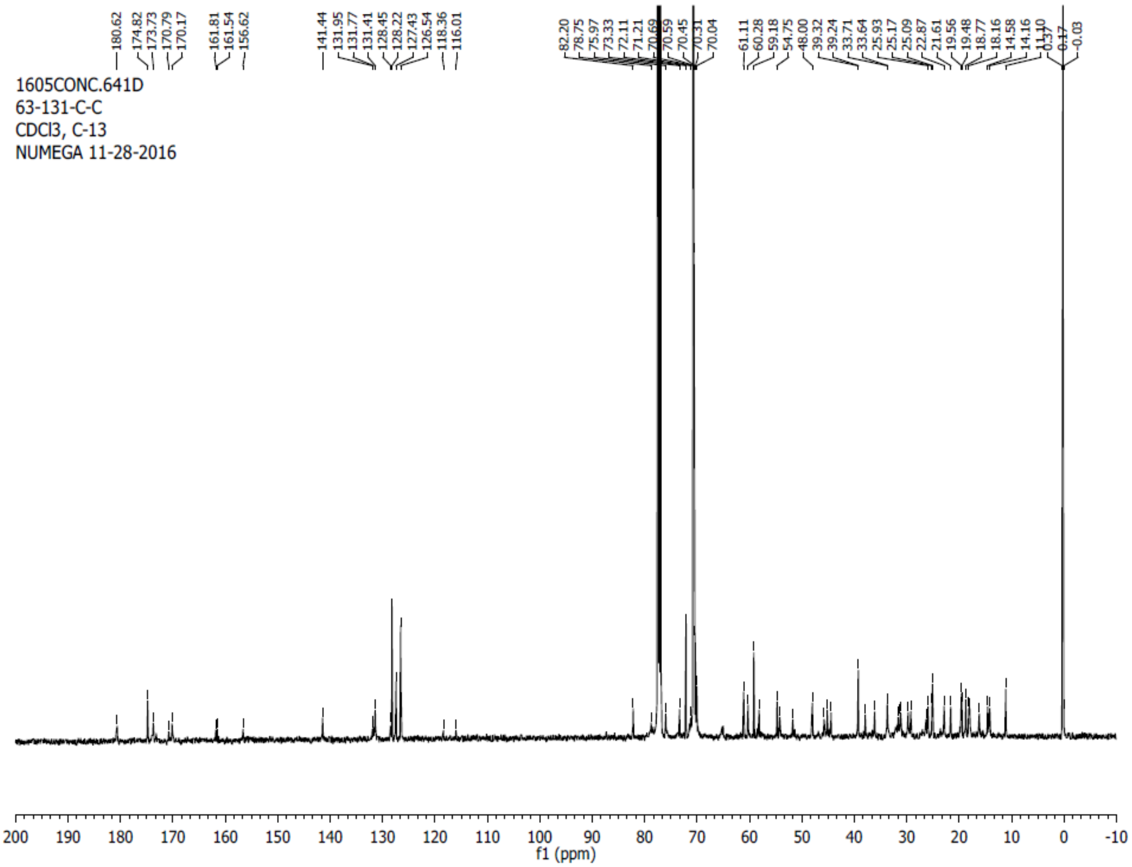

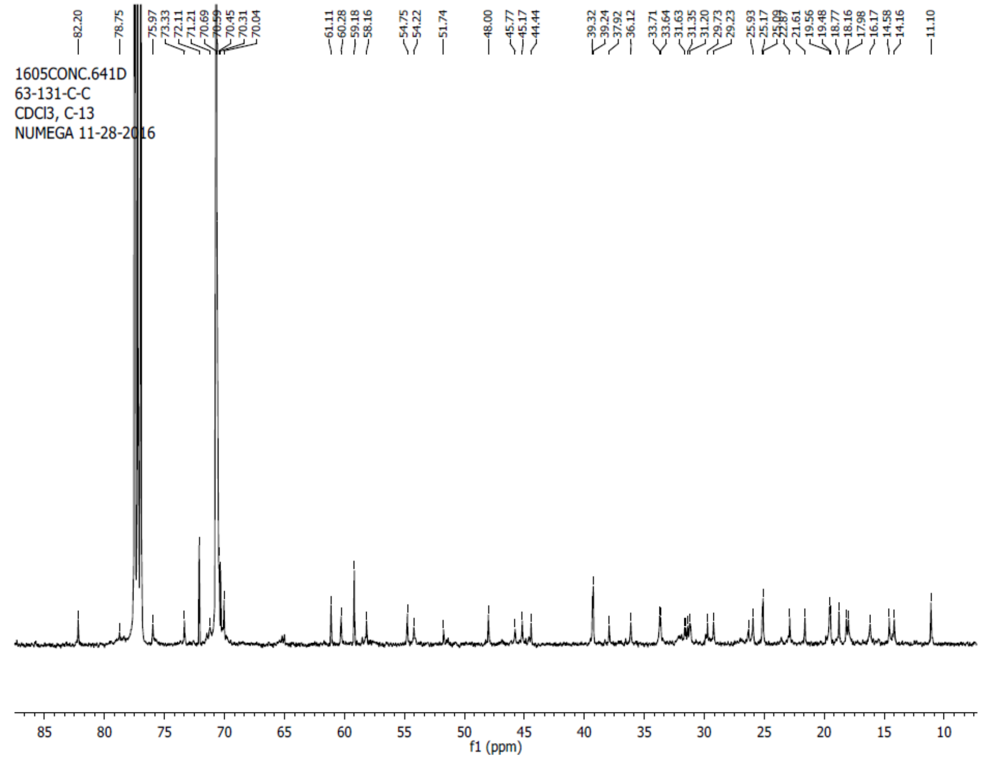

Supplementary Figure 69. ^{13}C NMR spectrum ($CDCl_3$) of compound S27.

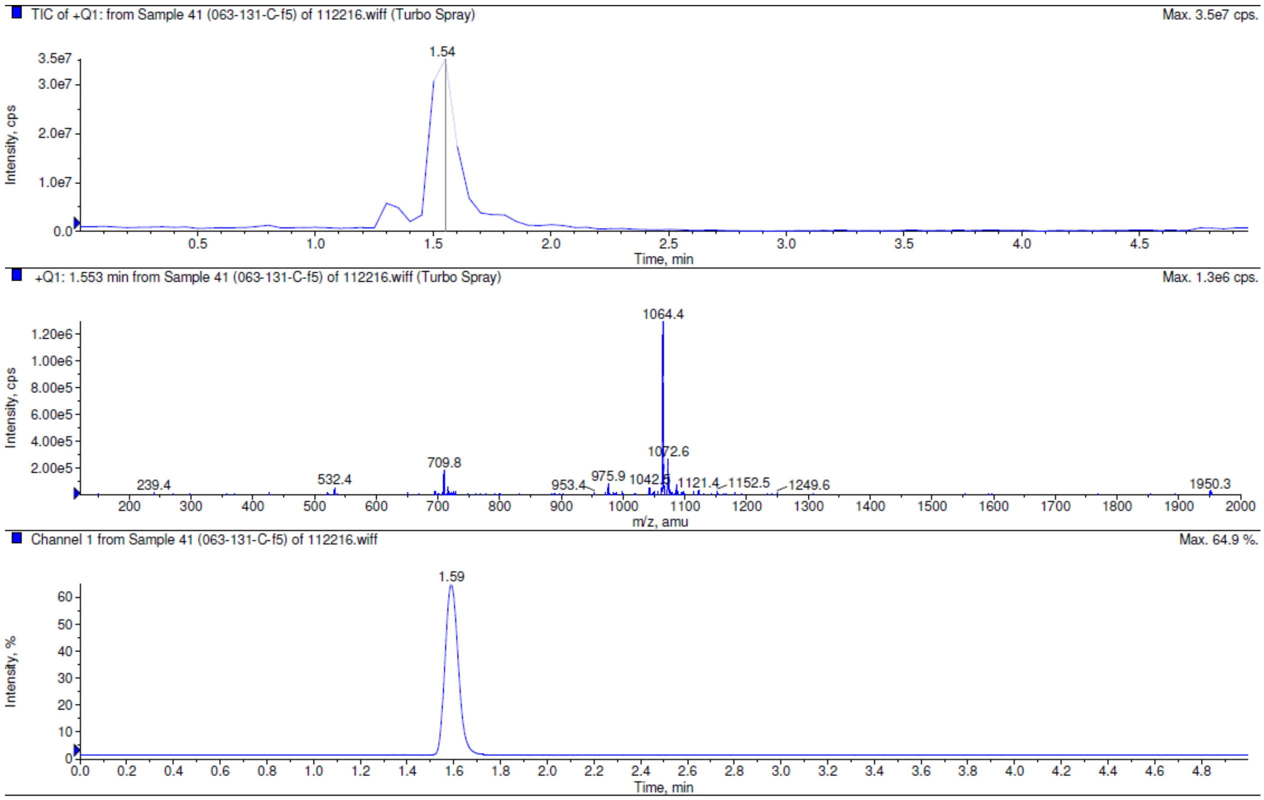

Supplementary Figure 70. LCMS chromatogram of compound S27.

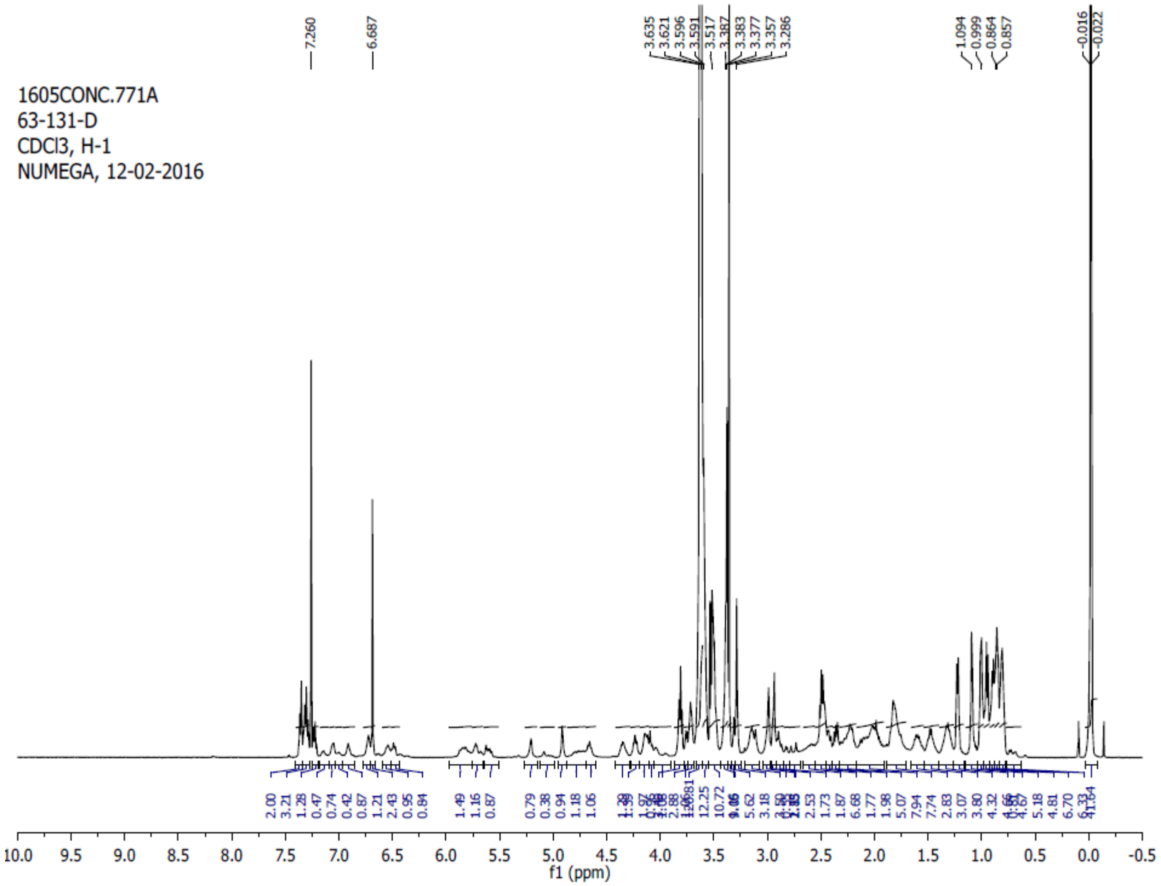

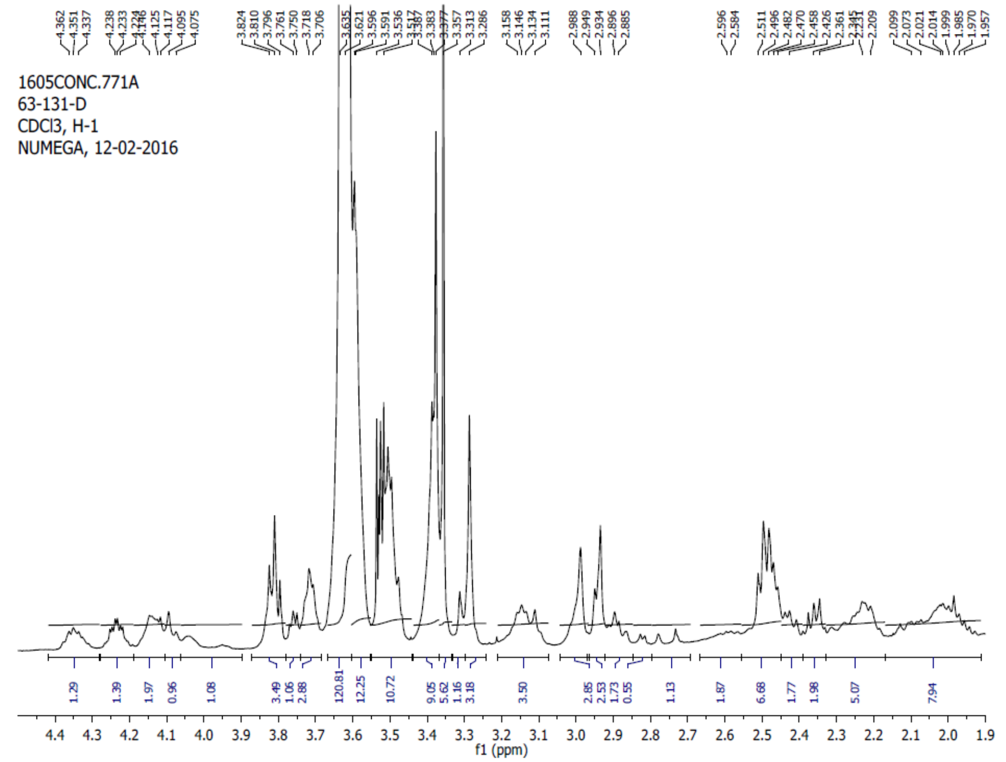

Supplementary Figure 71. ^{1}H NMR spectrum ($CDCl_3$) of compound 1.

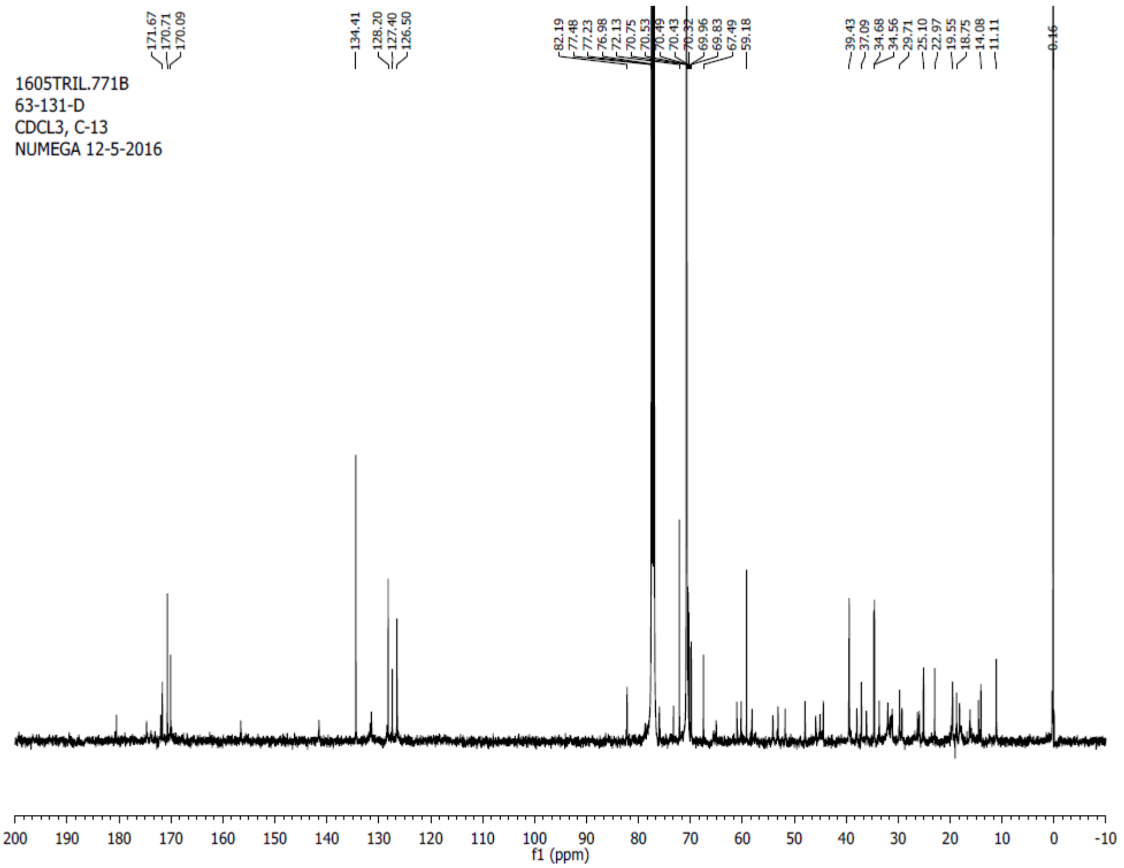

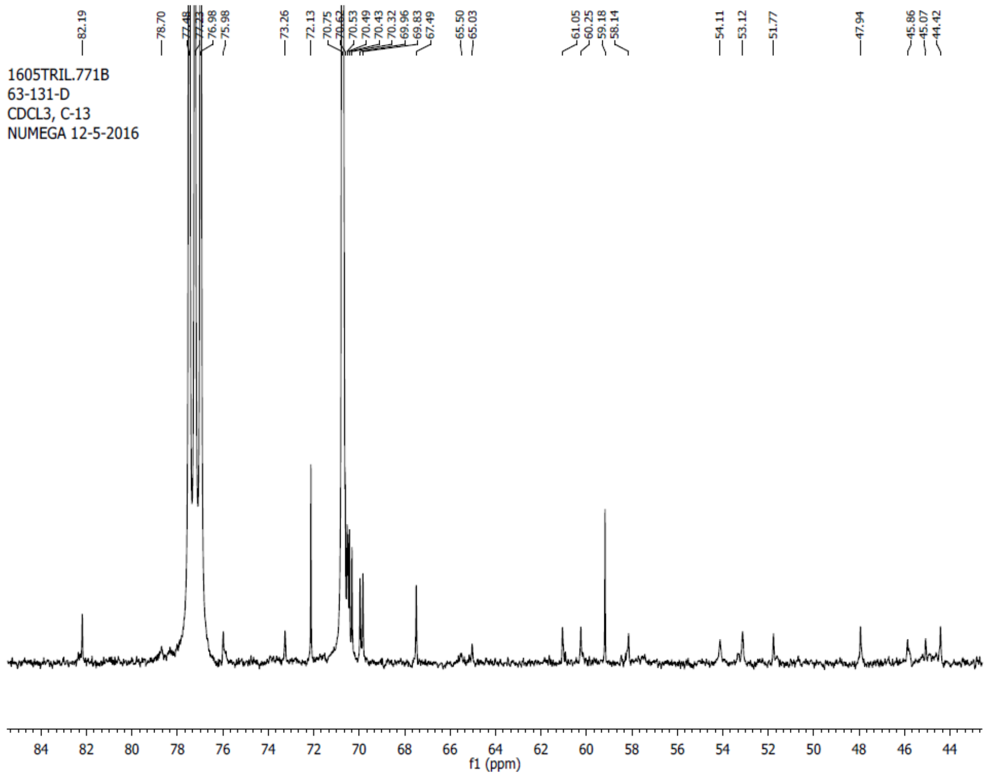

Supplementary Figure 72. ^{13}C NMR spectrum ($CDCl_3$) of compound 1.

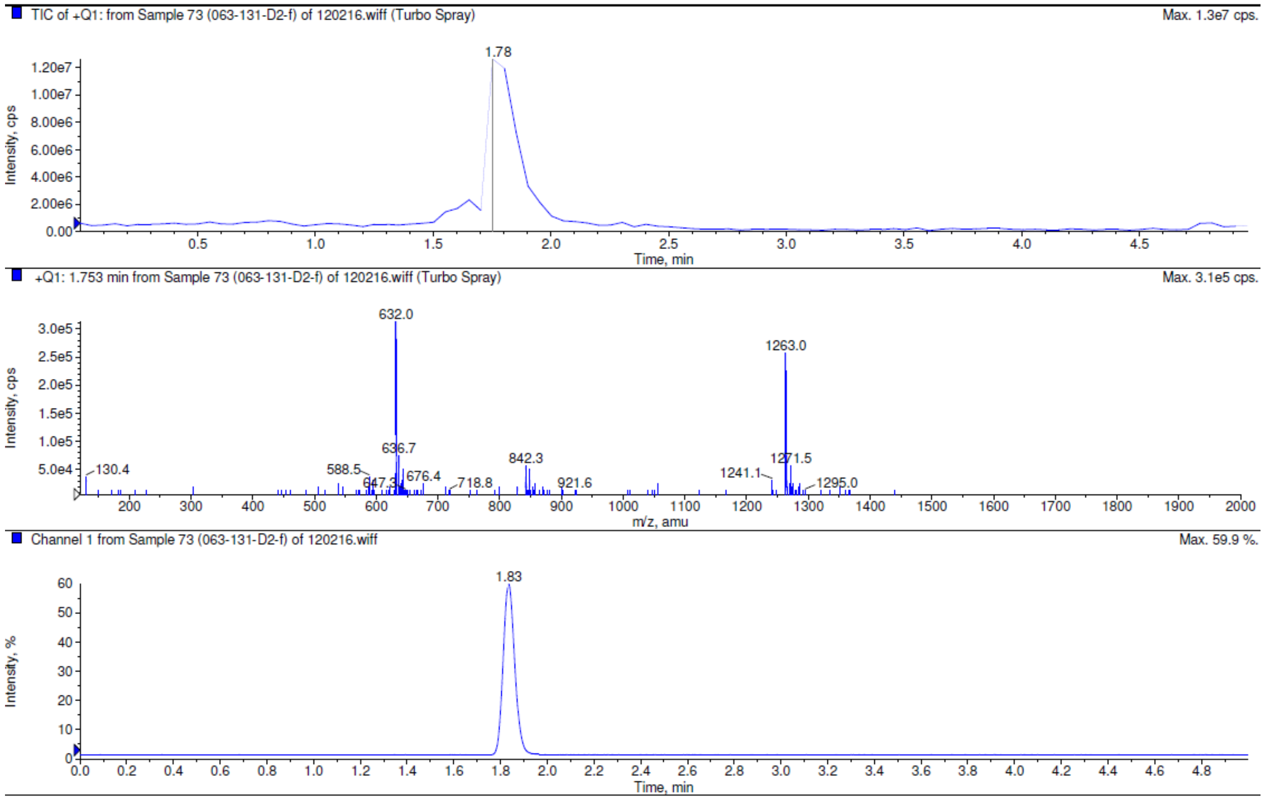

Supplementary Figure 73. LCMS chromatogram of compound 1.

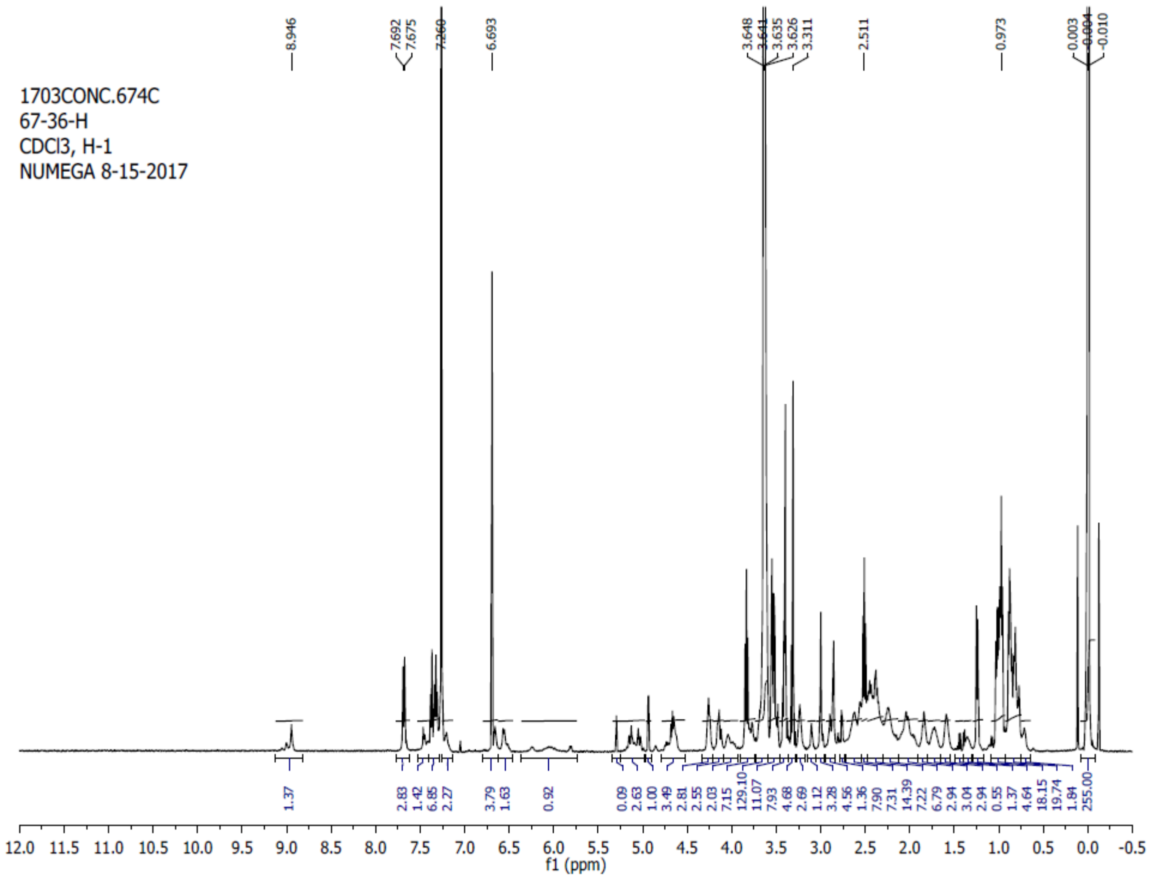

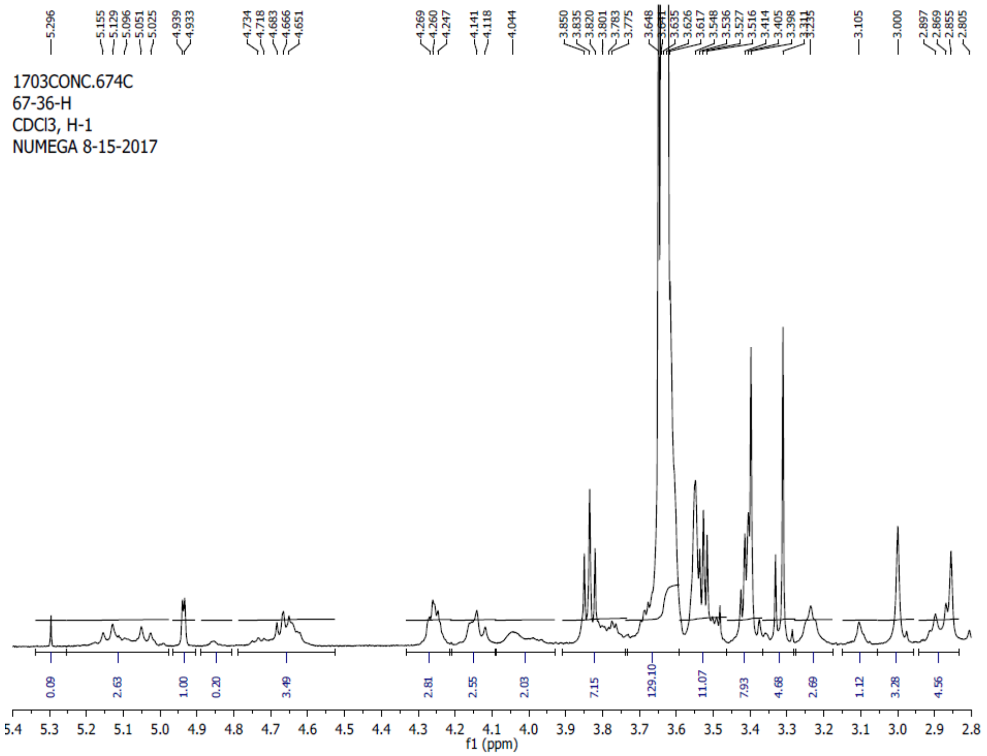

Supplementary Figure 74. **^{1}H NMR spectrum ($CDCl_3$) of compound 2.**

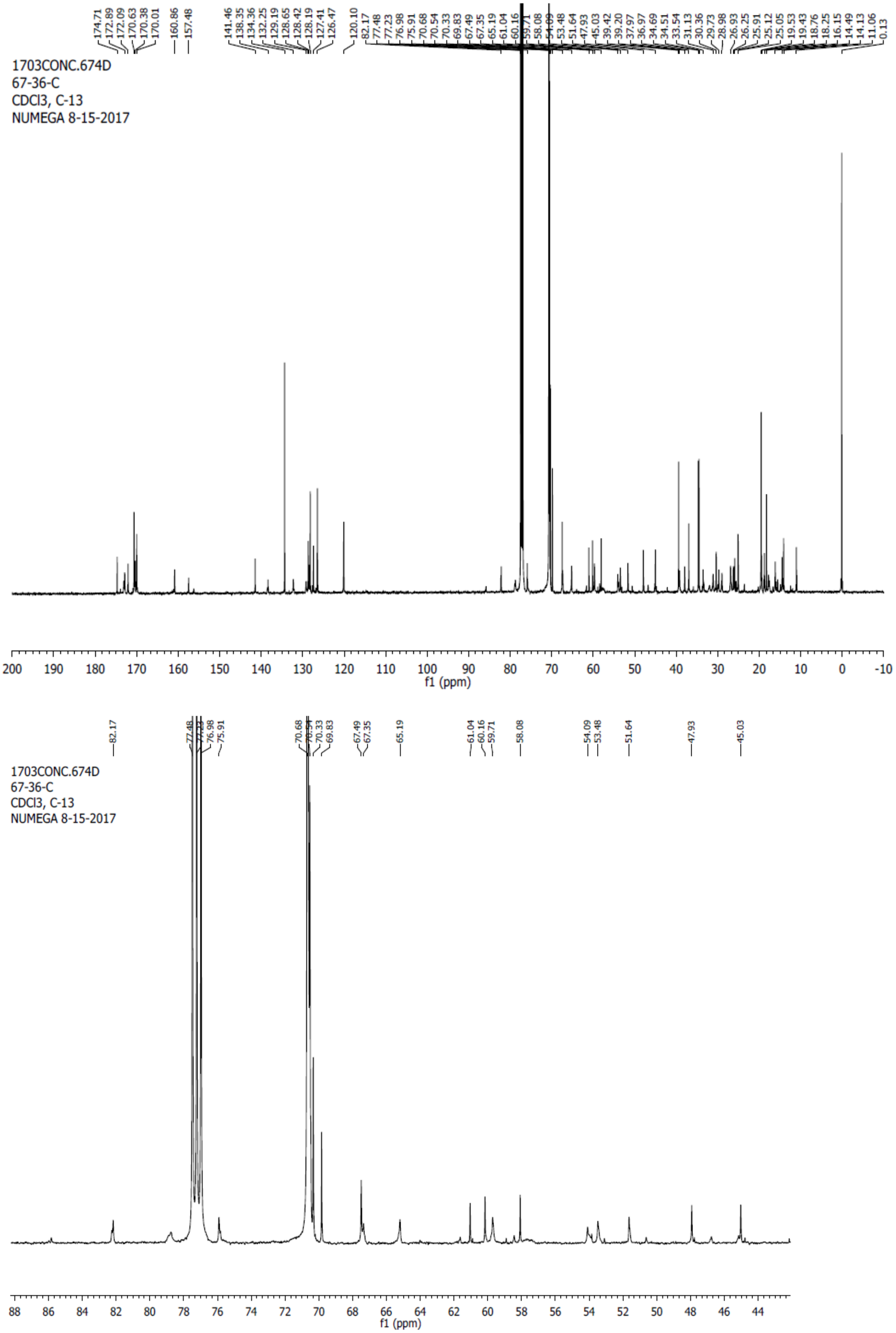

Supplementary Figure 75. ^{13}C NMR spectrum ($CDCl_3$) of compound 2.

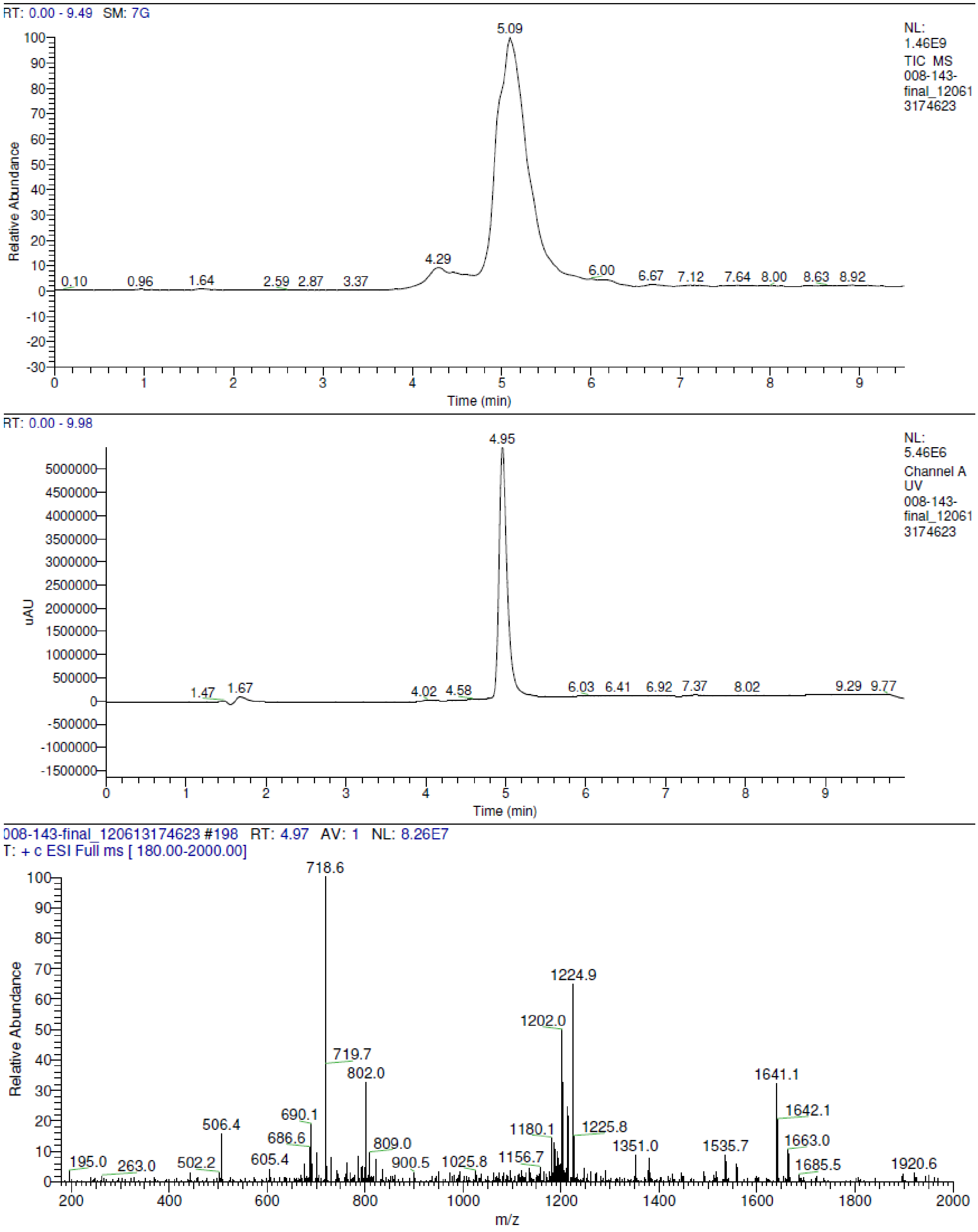

Supplementary Figure 76. LCMS chromatogram of compound 2.

Supplementary Tables

Supplementary Table 1. In vitro cytotoxicity assay: EC_{50} (half-maximal effective concentration) values in NIH:OVCAR-3, LS174T and HT-29 tumour cells.

**Compound**	**EC_{50}[a]**		
	NIH:OVCAR-3	LS174T	HT-29
**tc-ADC** + **3** [3 µM]	35 pM (28-47 pM)	185 pM (158-217 pM)	23 pM (21-26 pM)
**tc-ADC**	29 nM (18-48 nM)	71 nM (51-98 nM)	12 nM (8-17 nM)
**3**	0.79 mM (0.48-1.29 mM)	1.08 mM (0.31-3.67 mM)	> 10 mM
MMAE	39 pM (30-50 pM)	277 pM (236-325 pM)	29 pM (24-34 pM)

[a] 95% confidence interval is given in parentheses (n=3)

Supplementary Table 2. Summary of kinetic parameters for ^{18}F-labelled tetrazines in blood.

**Tetrazine**	**$t_{1/2,\alpha}$ (%)**	**$t_{1/2,\beta}$**	**AUC**
**S8**	1.57 min (70.9%)	20.0 min	132
**S12**	1.47 min (85.1%)	7.4 min	121
**S19**	1.19 min (81.9%)	8.4 min	67
**S24**	2.17 min (45.6%)	11.0 min	241

Supplementary Table 3. Blood half-life of ^{125}I-labelled **tc-ADC** administered at two different doses.

	**$t_{1/2,\alpha}$ (%)**	**$t_{1/2,\beta}$**	**$t_{1/2}$[a]**	**AUC**
1 mg/kg	0.71 h (55)	10.15 h	5.95 h	336.4
5 mg/kg	1.94 h (54)	12.55 h	7.17 h	374.3

[a] Calculated using $t_{1/2} = \ln2 \times AUC \times C_0^{-1}$

Supplementary Table 4. Biodistribution of ^{125}I-labelled **tc-ADC** administered at two different doses at 4 days post-injection. Data represent the mean % ID g^{-1} and % ID ± s.d. (n=4).[a]

Organ	1 mg kg^{-1}	5 mg kg^{-1}
	**% ID g^{-1}**	
Blood	0.108 ± 0.016	0.143 ± 0.034
Skin	0.056 ± 0.013	0.063 ± 0.011
Heart	0.045 ± 0.006	0.054 ± 0.012
Lung	0.086 ± 0.025	0.140 ± 0.021
Liver	0.073 ± 0.003	0.090 ± 0.010
Pancreas	0.025 ± 0.004	0.031 ± 0.004
Spleen	0.053 ± 0.008	0.085 ± 0.006
Kidney (left)	0.129 ± 0.006	0.133 ± 0.014
Fat	0.046 ± 0.008	0.047 ± 0.013
Muscle	0.019 ± 0.003	0.020 ± 0.003
Bone	0.055 ± 0.012	0.045 ± 0.008
Brain	0.004 ± 0.001	0.004 ± 0.001
	**% ID**	
Stomach	0.011 ± 0.006	0.009 ± 0.002
Small intestine	0.040 ± 0.007	0.044 ± 0.010
Large intestine	0.031 ± 0.007	0.034 ± 0.001
Thyroid	0.374 ± 0.066	0.423 ± 0.038

[a] Very low radioactivity levels were detected in all organs and tissues, including kidney, liver and full intestine. Low levels of radioactivity in thyroids and (full) stomachs confirm that the ADC did not dehalogenate in vivo.

Supplementary Table 5. Biodistribution of ^{125}I-labelled **tc-ADC**, **vc-ADC** and **nb-ADC** in LS174T, OVCAR-3 and HT-29 tumour-bearing mice at 48 h post-injection. Data represent the mean % ID g^{-1} and % ID ± s.d. (n=3-4).

**Organ**	**tc-ADC**			**vc-ADC**		**nb-ADC**	
	LS174T	OVCAR-3	HT-29	LS174T	OVCAR-3	LS174T	OVCAR-3[a]
				**% ID g^{-1}**			
Blood	0.60 ± 0.03	0.54 ± 0.11	0.54 ± 0.22	0.68 ± 0.13	1.13 ± 0.25	0.057 ± 0.007	0.15 ± 0.05
Tumour	29.39 ± 2.98	6.18 ±0.40	0.80 ± 0.25	25.79 ± 1.87	6.40 ± 1.28	0.24 ± 0.17	0.33 ± 0.11
Heart	0.21 ± 0.01	0.19 ± 0.03	0.17 ± 0.08	0.25 ± 0.03	0.35 ± 0.07	0.028 ± 0.003	0.05 ± 0.01
Lung	0.49 ± 0.06	0.45 ± 0.07	0.39 ± 0.16	0.69 ± 0.15	0.84 ± 0.12	0.09 ± 0.01	0.13 ± 0.01
Liver	0.51 ± 0.06	0.26 ± 0.05	0.23 ± 0.09	0.72 ± 0.16	0.45 ± 0.04	0.07 ± 0.01	0.10 ± 0.01
Spleen	0.25 ± 0.06	0.26 ± 0.04	0.22 ± 0.07	0.28 ± 0.07	0.23 ± 0.06	0.07 ± 0.01	0.12 ± 0.02
Pancreas	0.117 ± 0.004	0.10 ± 0.02	0.10 ± 0.05	0.15 ± 0.02	0.20 ± 0.04	0.015 ± 0.002	0.022 ± 0.004
Kidney L	0.35 ± 0.02	0.35 ± 0.03	0.45 ± 0.06	0.60 ± 0.06	0.71 ± 0.05	0.28 ± 0.02	0.32 ± 0.02
Muscle	0.07 ± 0.01	0.06 ± 0.01	0.06 ± 0.03	0.12 ± 0.05	0.15 ± 0.03	0.008 ± 0.001	0.014 ± 0.001
Bone	0.10 ± 0.01	0.11 ± 0.03	0.09 ± 0.03	0.10 ± 0.01	0.12 ± 0.03	0.027 ± 0.003	0.031 ± 0.002
Brain	0.018 ± 0.002	0.016 ± 0.004	0.02 ± 0.01	0.029 ± 0.004	0.05 ±0.01	0.0019 ± 0.0004	0.004 ± 0.001
				**% ID**			
Stomach	0.04 ± 0.01	0.034 ± 0.005	0.05 ± 0.03	0.07 ± 0.01	0.05 ±0.01	0.01 ± 0.01	0.010 ±0.001
Sm. int.	0.27 ± 0.03	0.28 ± 0.04	0.28 ± 0.14	0.36 ± 0.11	0.31 ± 0.06	0.04 ± 0.01	0.040 ± 0.007
Lg. int.	0.20 ±0.06	0.29 ± 0.02	0.17 ± 0.07	0.22 ± 0.02	0.25 ± 0.05	0.04 ± 0.01	0.04 ± 0.01
Thyroid	0.44 ± 0.19	0.86 ± 0.38	1.00 ± 0.60	0.54 ± 0.34	0.49 ± 0.15	0.57 ± 0.23	0.43 ± 0.10

[a] n=3

Supplementary Table 6. Biodistribution of [^{177}Lu]Lu-**3** at 1 and 24 h post-injection. Data represent the mean % ID g^{-1} and % ID ± s.d. (n=4).

**Organ**	**1 h**	**24 h**
	**% ID g^{-1}**	
Blood	0.05 ± 0.02	0.0025 ± 0.0005
Heart	0.03 ± 0.02	0.019 ± 0.004
Lung	0.12 ± 0.04	0.03 ± 0.01
Liver	0.08 ± 0.02	0.06 ± 0.01
Spleen	0.05 ± 0.02	0.039 ± 0.004
Pancreas	0.03 ± 0.01	0.02 ± 0.01
Kidney (left)	1.32 ± 0.35[a]	1.91 ± 0.12[a]
Muscle	0.05 ± 0.04	0.012 ± 0.004
Bone	0.05 ± 0.04	0.03 ± 0.01
Brain	0.01 ± 0.01	0.0010 ± 0.0002
	**% ID**	
Kidney (left)	0.29 ± 0.01	0.27 ± 0.04
Stomach	0.03 ± 0.01	0.02 ± 0.01
Small intestine	0.58 ± 0.27	0.08 ± 0.02
Large intestine	0.29 ± 0.36	0.11 ± 0.03

[a] The mice of the 1 h group were slightly heavier and had larger kidneys than those of the 24 h group (on average 0.2 gr *vs.* 0.15 g kidney) resulting in a lower % ID g^{-1} value for the 1 h group; the % ID values (i.e. not normalized per gram) are similar for the 1 and 24 h group, showing that the activity in the kidney was retained but did not increase over time.

Supplementary Table 7. Number of OVCAR-3 bearing mice removed from the single-dose efficacy study (based on pre-defined criteria) and median survival times.

	Tumour >1cm^3	Body weight loss	Poor physical condition	Median survival (days)
**tc-ADC** 0.75 mg kg^{-1} + **3**	7/8	0/8	0/8	83
**tc-ADC** 1.50 mg kg^{-1} + **3**	5/7	0/7	0/7	107
**tc-ADC** 3.75 mg kg^{-1} + **3**	3/9	2/9	2/9	62
**tc-ADC** 7.50 mg kg^{-1} + **3**	1/11	2/11	2/11	NA
**3**	7/9	0/9	1/9	55
vehicle	7/9	0/9	0/9	44
**tc-ADC** 7.5 mg kg^{-1}	6/7	0/7	0/7	62

Supplementary Table 8. Number of OVCAR-3 bearing mice removed from the multi-dose efficacy study (based on pre-defined criteria) and median survival times.

	Tumour >1cm^3	Body weight loss	Poor physical condition	Median survival (days)
**tc-ADC** + **3**	0/8	0/8	1/8	NA
**tc-ADC**	5/8	0/8	2/8	69
**vc-ADC**	3/8	1/8	3/8	86.5
**nb-ADC** + **3**	8/8	0/8	0/8	41
vehicle	7/8	0/8	0/8	55
**3**	8/8	0/8	0/8	48

Supplementary Table 9. Number of LS174T bearing mice removed from the multi-dose efficacy study (based on pre-defined criteria) and median survival times.

	Tumour >1cm^3	Body weight loss	Poor physical condition	Median survival (days)
**tc-ADC** 1 mg kg^{-1} + **3**	7/9	0/9	2/9	17
**tc-ADC** 3 mg kg^{-1} + **3**	8/10	2/10	0/10	34
**tc-ADC** 5 mg kg^{-1} + **3**	8/10	1/10	1/10	39
**tc-ADC** 3 mg kg^{-1}	9/10	0/10	1/10	13
**vc-ADC** 3 mg kg^{-1}	1/8	1/8	6/8	14.5
**nb-ADC** 3mg kg^{-1} + **3**	7/10	0/10	3/10	13
vehicle	9/10	0/10	1/10	12
**3**	7/10	0/10	3/10	14

Supplementary Table 10. Criteria for the assessment of renal damage[7].

Grade	Criteria
**0**	No irregularities
**1**	Little dilation of tubules; no basal membrane thickening
**2**	More pronounced tubule dilatation, basal membrane thickening and mitotic activity
**3**	Shrinkage of a small number of glomeruli; flat or lost tubule epithelium, strong tubule dilatation and more pronounced basal membrane thickening
**4**	Increased shrinkage of glomeruli leading to optical emptiness; strongly dilated tubules and signs of peripheral fibrosis

Supplementary Methods

Materials.

All reagents, chemicals, materials and solvents were obtained from commercial sources, and were used as received: Biosolve, Merck and Cambridge Isotope Laboratories for (deuterated) solvents, and Aldrich, Acros, ABCR, Merck, Fluka, and Fluorochem for chemicals, materials and reagents. All solvents were of AR quality. Tablets for the preparation of phosphate buffered saline solution (PBS) were purchased from Calbiochem. Water was distilled and deionized (18 MΩcm) by means of a milli-Q water filtration system (Millipore). O-(2-Aminoethyl)-O′-[2-(Boc-amino)ethyl]decaethylene glycol was obtained from Polypure. Monomethyl auristatin E (MMAE) was purchased from Selleck Chemicals. [^{177}Lu]Lutetium chloride and sodium [^{125}I]iodide solutions were purchased from PerkinElmer. [^{111}In]Indium chloride solution was purchased from Mallinckrodt Pharmaceuticals. The Bolton-Hunter reagent (N-succinimidyl-3-[4-hydroxyphenyl]propionate, SHPP) and Zeba desalting spin columns (7 and 40 kDa MW cut-off, 0.5 mL) were purchased from Pierce Protein Research (Thermo Fisher Scientific). Amicon Ultra centrifugal filter units (10 kDa MW cut-off) and PD-10 desalting columns were purchased from Merck and GE Healthcare Life Science, respectively. Mouse serum was purchased from Innovative Research.

General Methods.

^{1}H NMR and ^{13}C NMR spectra were recorded on a Bruker 400 Ultrashield NMR spectrometer (400 MHz for ^{1}H NMR and 100 MHz for ^{13}C NMR). Chemical shifts are reported in ppm downfield from TMS at 25°C. Abbreviations used for splitting patterns are s=singlet, t=triplet, q=quartet, m=multiplet and br=broad. Reverse phase (RP) medium pressure liquid column chromatograpy was performed on a Biotage Isolera One MPLC system using a GracePure C_{18} RP column (40 gram), and acetonitrile / water mixtures (containing 0.1 v/v% formic acid) as the eluent. HPLC-MS/PDA was performed using a Shimadzu LC-10 AD VP series HPLC coupled to a diode array detector (Finnigan Surveyor PDA Plus detector, Thermo Electron Corporation) and an Ion-Trap (LCQ Fleet, Thermo Scientific) MS-detector, employing an Alltech Alltima HP C_{18} 3μ column using an injection volume of 1-4 µL, a flow rate of 0.2 mL min^{-1} and typically a gradient (5% to 100% in 10 min, held at 100% for 3 min) of acetonitrile in H_2O (both containing 0.1 v/v% formic acid) at 35°C. Preparative RP-HPLC (acetonitrile / H_2O with 0.1 v/v% formic acid) was performed using a Shimadzu SCL-10A VP coupled to two Shimadzu LC-8A pumps and a Shimadzu SPD-10AV VP UV-vis detector on a Phenomenex Gemini 5μ C_{18} 110A column. Size exclusion chromatography (SEC) was performed on an Akta system equipped with a Superdex 200 column. HPLC-QTOF-MS analysis was performed on a Waters Acquity UPLC system equipped with a Sample Manager and a Xevo G2 Quadrupole Time of Flight (QTOF) detector, applying Zspray lockspray ionisation. Mass Lynx v4.1 software was used. Radio-HPLC was performed on an Agilent 1100 system, equipped with a Gabi radioactive detector (Raytest). The samples were loaded on an Alltima C_{18} column (4.6 × 150mm, 5μ), which was eluted at 1 mL min^{-1} with a linear gradient of water (A) and acetonitrile (B) containing 0.1% v/v% TFA (4 min at 20% B followed by an increase to 70% B in 11 min). Radio-ITLC was performed on ITLC-SG strips (Varian Inc.) eluted with 200 mM EDTA in saline solution (^{111}In- and ^{177}Lu-labeling) or a 1:1 mixture of methanol/ethyl acetate (^{125}I-labeling). In these conditions the radiolabelled products remain at the base while unbound ^{111}In/^{177}Lu and [^{125}I]I-SHPP migrates with an R_f

of 0.7-0.9. Radio-TLC for kinetics measurements was performed on silica gel RP-8 plates (Merck) eluted with a 40:60 mixture of water and acetonitrile (containing 0.1 v/v% TFA). In these conditions the ADC-bound ^{177}Lu-probe remains at the base while the unreacted probe migrated with a R_f of ca. 0.5. SDS polyacrylamide gel electrophoresis (SDS-PAGE) was performed on a Mini-PROTEAN Tetra Cell system using 4-15% precast Mini-PROTEAN TGX gels and Precision Plus Protein All Blue Prestained protein standards (Bio-Rad). The radioactivity distribution on TLC plates and SDS-PAGE gels was monitored with a Typhoon FLA 7000 phosphor imager (GE Healthcare Life Science) using the AIDA software.

ADC production and characterization.

The anti-TAG72 diabody AVP04-58 (MW 51088 Da) and the anti-PSMA diabody AVP06 (MW 49578 Da) were provided by Avipep Ltd. AVP04-58 was prepared as previously described[8] without the C-terminal His_6 tag, and differs from the parent anti-TAG72 diabody AVP04-07[8] by the substitution of cysteine residues into positions L8 and L11 in the VL domain, thus affording two additional cysteine residues per scFv monomer (4 cysteines per diabody). These 4 cysteine residues were used for site-specific conjugation of maleimide-TCO-MMAE **1** (for **tc-ADC**) and maleimide-Val-Cit-PABC-MMAE **2** (for **vc-ADC**). The anti-PSMA diabody AVP06 has a similar L8 and L11 cysteine substitution into the VH and VL domains and the V-domains were both taken from the deimmunised J591 sequence previously reported (SeqID#5/19)[9]. This diabody was site-specifically conjugated with maleimide-TCO-MMAE **1** to obtain the non-binding **nb-ADC** control.

Diabody functionalization was achieved via maleimide chemistry as described previously[8], by reduction of the disulfide bonds with dithiothreitol (DTT) followed by diabody reaction with **1** or **2**. Briefly, a ca. 2 mg mL^{-1} diabody solution in degassed 100 mM phosphate buffer pH 6.8 containing 2 mM EDTA (EDTA-PB) was combined with freshly dissolved 100 mM DTT in EDTA-PB (6 mM final DTT concentration) and incubated at room temperature for 1 h. The reduced diabody was then loaded on a PD-10 column and eluted from the column with EDTA-PB, and immediately afterwards combined with a solution of **1** or **2** (7.5 eq. per SH) in DMSO (ca. 15% DMSO v/v% in the final mixture) and incubated overnight at 4°C. Subsequently the ADC was purified by gel filtration on a AKTA system equipped with a Superdex 75 26/60 prep-grade column which was eluted with EDTA-PB at 2.5 mL min^{-1}. The purified fractions were combined and mixed with 5% DMSO . The solution was then concentrated using Amicon Ultra-15 centrifugal filters and the ADC concentration was measured by NanoDrop.

The ADCs were then analysed by SEC (Supplementary Fig. 1a-1c), SDS-PAGE (Supplementary Fig. 1d) and ESI-TOF MS (Supplementary Fig. 2 and 3) confirming the identity of the conjugates with drug-to-antibody ratios (DARs) of 4 in the presence of only trace amounts of aggregates.

Tetrazine radiofluorination.

Four tetrazine precursors containing an aminooxy function and different linkers (**S7**, **S11**, **S18**, and **S23**) were radiolabelled using the [^{18}F]fluorobenzaldehyde ([^{18}F]FBA) synthon. The radiofluorination reactions were performed in a custom-made Eckert&Ziegler modular lab, which was operated semi-automatically. [^{18}F]FBA was synthesized according to standard procedures starting from [^{18}F]fluoride/K_{222} and 4-formyl-N,N,N-trimethylanilinium triflate (ABX; ca. 6 mg, 10 min reaction at 100°C in 0.7 mL DMSO) and was

reacted with the tetrazine precursors (ca. 2 mg) in 0.1 M ammonium formate (pH 2.5) : MeOH (1:9) at 60-70°C for 20 min. The coupling reaction afforded a 30-80% yield, depending on the amount of precursor used. After reaction the ^{18}F-labelled tetrazines were purified by prep-HPLC using a SymmetryPrep C_{18} column (7.8×300 mm, 7 µm, Waters) eluted with H_2O/acetonitrile (containing 0.1 v/v% TFA) either isocratically or with a linear gradient and a 7 mL min^{-1} flow. Subsequently, the purified ^{18}F-tetrazines were trapped on a solid-phase extraction cartridge (C_{18} sep-pak light) and eluted with ethanol. The ethanolic solutions were concentrated at 70°C under a gentle stream of Ar (ca. 10 min) and analysed by radio-HPLC (Supplementary Fig. 10a) on an Agilent 1100 system equipped with a Symmetry C_{18} column (3.9×150 mm, 5 µm, Waters). The four ^{18}F-labelled tetrazines were combined with the respective ^{19}F-containing analogues (**S8**, **S12**, **S19**, and **S24**) to the desired molar activity (*vide infra*) and with gentisic acid for animal experiments.

Tetrazine ^{177}Lu/^{111}In-labeling.

The activator precursor **S4** and probe **4** were labelled with no-carrier added lutetium-177 and indium-111 in 0.2 M ammonium acetate pH 5.5 (^{177}Lu) or 6.0 (^{111}In), for 5-10 min at 60°C. Incubation was followed by a DTPA challenge (5 µL of a 10 mM DTPA solution) to chelate any unbound radiometal. In these conditions the radiolabelling yield was always greater than 98% and the radiochemical purity greater than 95%, as confirmed by radio-ITLC and radio-HPLC (Supplementary Fig. 10b and 10c), and the radiolabelled compounds were used in animal and in vitro experiments without any further purification. For animal experiments, [^{177}Lu]Lu-**3** was combined with the non-radioactive analogue to the desired molar activity (*vide infra*).

^{18}F-Labelled tetrazine probes in vivo evaluation: blood kinetics.

Four groups of LS174T tumour-bearing mice (n=4) were injected with ^{18}F-labelled tetrazines **S8**, **S12**, **S19** and **S23** (ca. 0.335 µmol kg^{-1}, 7-8 MBq in 80 µL PBS containing 100 µg gentisic acid per mouse) and the mice were serially bled via the vena saphena at 2, 5, 10, 20, 40 and 60 min post-tetrazine injection (ca. 20 µL per sample). Three hours post-injection the mice were euthanized, blood was obtained by cardiac puncture and selected organs and tissues were harvested for γ-counting (400-600 keV energy window). The half-lives in blood were calculated by fitting the data points to bi-exponential curves, as shown in Supplementary Fig. 14b. All tested ^{18}F-tetrazines showed rapid elimination from blood: most of the injected **S8**, **S12** and **S19** (70-85%) eliminated from blood within the first few minutes, whereas **S24** was retained longer and, as a result, the area under the curve (AUC) was 2-to-3 fold higher than for the other tracers (Supplementary Table 2).

^{18}F-Labelled tetrazine probes in vivo evaluation: tumour binding.

Four groups of LS174T tumour bearing mice (n=4) were pretreated with an anti-TAG72 [^{125}I]I-CC49-TCO conjugate[10] followed by two doses of clearing agent, according to an optimized tumour pretargeting protocol[11]. Two hours after clearing, the mice were injected the four ^{18}F-labelled tetrazine probes **S8**, **S12**, **S19**, and **S24** (ca. 0.335 µmol kg^{-1}, 7-9 MBq in 80 µL PBS containing 100 µg gentisic acid per mouse). Three hours post-tetrazine injection the mice were euthanized, a blood sample was obtained by cardiac

puncture and tumours and other tissues were harvested for γ-counting using a 400-600 keV energy window for ^{18}F. The same samples were measured again 72 h later using a 10-80 keV energy window for ^{125}I. The data obtained for ^{18}F-labelled tetrazines in CC49-TCO pretreated mice were compared to the 3 h biodistribution data from the previous blood kinetic study, obtained from mice injected with tetrazine probes only (Supplementary Fig. 14c).

In vivo reaction between ADC and activator: direct binding study

To further confirm on-tumour reaction between **tc-ADC** and activator **3** a direct binding study was carried out. A low dose of activator **3** was used in this study in order to quantify the extent of activator uptake in the tumour on a % ID g^{-1} basis. One group of LS174T tumour-bearing mice (n=3) was injected with **tc-ADC** (2 mg kg^{-1}) followed by 13 nmol kg^{-1} [^{177}Lu]Lu-**3** (1.5 MBq in 100 µL saline per mouse) 48 h later. A second group of mice was administered the same dose of [^{177}Lu]Lu-**3** without pre-administration of **tc-ADC**. All mice were euthanized 3 h post-activator injection and tumours and other tissues were harvested for γ-counting (Supplementary Fig. 16d).

MMAE concentration measurements

Groups of 3 mice bearing LS174T xenografts were injected with **tc-ADC** (2 mg kg^{-1}) followed by 0.335 mmol kg^{-1} activator **3** (in 130 µL PBS containing 5% DMSO) or vehicle 48 h later. Seventy-two or 96 h post-ADC administration, the mice were euthanized (Fig. 4d). One extra group of mice was injected **vc-ADC** (2 mg kg^{-1}) and euthanized 24 h post-injection. Tumour, liver and plasma samples were harvested from all groups, weighed and the MMAE concentration in tumour and liver samples was evaluated as described by Burke *et al.*[12]. Briefly, the samples (0.1-0.2 g) were transferred into MagNA Lyser green beads tubes (Roche) together with 1 mL methanol and d8-MMAE (MedChem Express) as internal standard. The tissues were homogenized (4×30 sec cycles, 6500 rpm, with 1 min cooling in between cycles) and the debris was removed by centrifugation (13,000 rpm, 5 min). The methanol was evaporated under a stream of N_2, the residue was reconstituted in water containing 10% acetonitrile and the solution was filtered through a 0.22 µm filter. The plasma samples were combined with d8-MMAE and the plasma proteins were precipitated by adding two parts of ice-cold acetonitrile. After vortexing, 10 min standing at -20°C and centrifugation (13,000 rpm, 5 min), the supernatants were separated from the protein pellets, diluted with five parts of PBS, and filtered. All samples were then analysed by the LC-QTOF-MS method in Supplementary Fig. 7 to quantify the amount of free MMAE based on the ratios with d8-MMAE. Tumour, liver and plasma samples from non-treated mice added with **tc-ADC** and/or d8-MMAE were used as controls. The limit of detection for MMAE in this assay was 0.2 nM.

SPECT/CT imaging

The SPECT/CT images were acquired using a dedicated small-animal SPECT/CT scanner (U-SPECT-II, MILabs) using a 1.0 mm pinhole general rat and mouse collimator and a total acquisition of 30 min. CT images were acquired for anatomical information (65 keV, 615 mA, 160-um spatial resolution). The SPECT images were reconstructed using the U-SPECT software (U-SPECT-Rec, MILabs) with the following settings: ordered-subsets expectation maximization, 3 iterations, 32 subsets, and a voxel size of

0.4 mm^3. Indium-111 uptake in volumes of interest drawn over tumour, kidney and muscle was quantified using Inveon Reconstruction Workplace (Siemens Healthcare).

Organic Syntheses

N-Boc-(3-(6-methyl-1,2,4,5-tetrazin-3-yl)propylamine (S1)

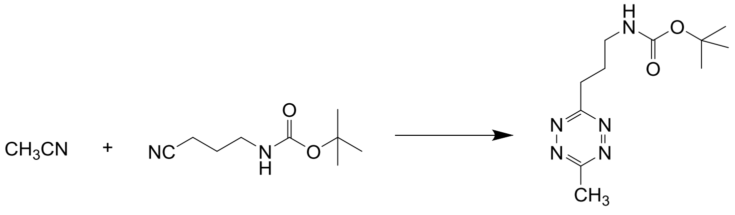

S1

3-Cyano-N-Boc-propylamine was prepared according to Houssin *et al.*[13]. 3-Cyano-N-Boc-propylamine (8.83 g; 48.0 mmol) was dissolved in acetonitrile (12.5 mL; 240 mmol) and ethanol (25 mL), hydrazine hydrate (70 mL), and zinc trifluoromethanesulfonate (10.4 g; 28.7 mmol) were added. The mixture was stirred at 60°C in an inert atmosphere for 22 h, and subsequently concentrated in vacuo. To the pink, turbid oil was added THF (100 mL), water (100 mL), and acetic acid (50 mL), and the mixture was cooled in an ice bath. Sodium nitrite (39.6 g; 574 mmol) was added in small portions over a period of 30 min and the mixture was stirred for an additional 60 min at 20°C, and the concentrated in vacuo. To the pink residue was added water (250 mL) and the crude product was extracted with methylene chloride (2 times 250 mL). The combined organic layers were washed with 0.5 M aqueous citric acid (100 mL), and 0.5 M aqueous sodium carbonate (100 mL), dried over sodium sulfate, and evaporated to dryness. The crude product was purified by column chromatography (silica gel, ethyl acetate / heptane = 1:3) and recrystallized from diisopropyl ether at -20°C, to yield product **S1** as pink crystals (2.31 g; 19%). ^1H NMR ($CDCl_3$): δ = 4.69 (br s, 1H), 3.35 (t, J = 7.6 Hz, 2H), 3.28 (q, J = 6.5 Hz, 2H), 2.15 (m, 2H), 1.44 (s, 9H) ppm. ^{13}C NMR ($CDCl_3$): δ = 169.50, 167.45, 155.88, 79.36, 39.71, 31.96, 28.39, 28.34, 21.10 ppm. HPLC-MS/PDA (5% to 100% in 10 min): t_r=5.33 min (*m/z*=+137.08, +154.08, +198.00, +253.92 $[M+H]^+$; calcd 254.16 for $C_{11}H_{20}N_5O_2$; λ_{max}=277, 524 nm). See Supplementary Fig. 25 and 26.

5-((3-(6-Methyl-1,2,4,5-tetrazin-3-yl)propyl)amino)-5-oxopentanoic acid (S2)

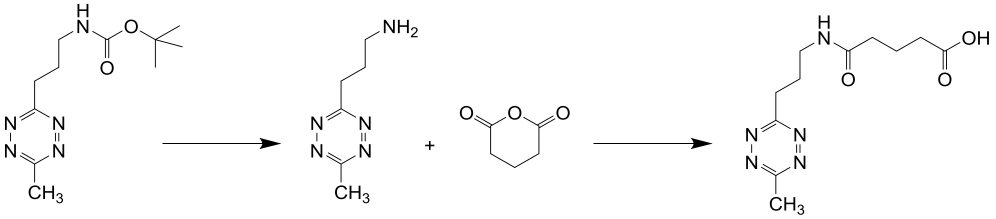

S1 **S2**

N-Boc-(3-(6-methyl-1,2,4,5-tetrazin-3-yl)propylamine (**S1**) (1.00 g; 3.95 mmol) was dissolved in methylene chloride (10 mL), and trifluoroacetic acid (5 mL) was added, and the pink solution was stirred at 20°C for 30 min, and subsequently concentrated, coevaporated with acetonitrile (2 times 10 mL), and dried in vacuo. HPLC-MS/PDA (5% to 100% in 10 min): t_r=0.6 min (m/z=+154 Da $[M+H]^+$; calcd 154.11 for $C_6H_{12}N_5$; λ_{max}=277, 524 nm).

The intermediate was dissolved in acetonitrile (50 mL), and triethylamine (5.5 mL) was added, followed by glutaric anhydride (0.45 g; 3.95 mmol). The pink solution was stirred at 20°C in an inert atmosphere for 1 h, and subsequently concentrated in vacuo. The pink residue was dissolved in ethyl acetate (50 mL) and washed with hydrochloric acid (50 mL, 3 M). The organic layer was isolated, and the aqueous layer was extracted with ethyl acetate until it became almost colourless. The combined organic layers were dried over sodium sulfate and concentrated in vacuo to give a pink oil. This crude product was triturated with cold diethyl ether (20 mL), to yield product **S2** as a pink powder (0.73 g; 69%). ^{1}H NMR ($CDCl_3$): δ = 6.11 (br s, 1H), 3.41 (q, *J* = 6.5 Hz, 2H), 3.35 (t, *J* = 7.6 Hz, 2H), 3.05 (s, 3H), 2.43 (t, *J* = 7.0 Hz, 2H), 2.31 (t, *J* = 7.3 Hz, 2H), 2.18 (m, 2H), 1.98 (m, 2H) ppm. ^{13}C NMR ($CDCl_3$): δ = 176.83, 173.31, 169.24, 167.46, 38.68, 35.19, 33.04, 31.88, 27.57, 21.00, 20.79 ppm. HPLC-MS/PDA (5% to 100% in 10 min): t_r=2.72 min (*m/z*=+268.17 Da $[M+H]^+$; calcd 268.14 for $C_{11}H_{18}N_5O_3$; λ_{max}=278, 518 nm). See Supplementary Fig. 27-29.

***tert*-Butyl (45-(6-methyl-1,2,4,5-tetrazin-3-yl)-37,41-dioxo-3,6,9,12,15,18,21,24,27,30,33-undecaoxa-36,42-diazapentatetracontyl)carbamate (S3)**

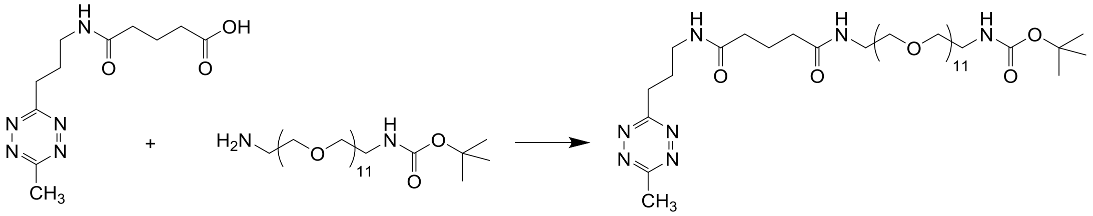

5-((3-(6-Methyl-1,2,4,5-tetrazin-3-yl)propyl)amino)-5-oxopentanoic acid (**S2**) (635 mg; 2.38 mmol) was dissolved in DMF (25 mL). Subsequently, O-(2-aminoethyl)-O′-[2-(Boc-amino)ethyl]decaethylene glycol (1.53 g; 2.38 mmol), DIPEA (1.23 g; 9.52 mmol), and PyBOP (1.24 g; 2.38 mmol) were added. The reaction mixture was stirred at 20°C in an inert atmosphere for 30 min, and then concentrated in vacuo. The residue was dissolved in chloroform (40 mL) and washed with 0.5 M citric acid (2 times 30 mL) and saturated sodium carbonate (2 times 30 mL). The organic layer was dried over sodium sulfate and evaporated to dryness. The crude product was dissolved in chloroform (7 mL) and precipitated in cold diisopropyl ether. The precipitate was collected by centrifugation and decantation, redissolved in chloroform (10 mL), and evaporated to dryness, to yield product **S3** as a pink solid (2.03 g; 95%), containing a trace amount of tri(pyrrolidin-1-yl) phosphine oxide. ^{1}H NMR ($CDCl_3$): δ = 6.39 (s, 1H), 6.37 (s, 1H), 5.05 (br s, 1H), 3.85 – 3.59 (m, 40H), 3.59 – 3.49 (m, 4H), 3.44 (d, *J* = 5.5 Hz, 2H), 3.41 – 3.23 (m, 6H), 3.04 (s, 3H), 2.27 (td, *J* = 7.1, 2.0 Hz, 4H), 2.17 (m, 2H), 1.96 (m, 2H), 1.44 (s, 9H) ppm. ^{13}C NMR ($CDCl_3$): δ = 172.73, 172.66, 169.35, 167.48, 155.96, 70.54 (m), 70.21, 70.19, 69.67, 46.28, 46.24, 40.35, 39.21, 38.41, 35.19, 35.12, 31.97, 28.41, 27.88, 26.44, 26.36, 21.84, 21.08 ppm. HPLC-

MS/PDA (5% to 100% in 10 min): t_r=5.05 min (*m/z*=+894.33 Da $[M+H]^+$; calcd 894.54 for $C_{40}H_{76}N_7O_{15}$; λ_{max}=277, 523 nm). See Supplementary Fig. 30 and 31.

2,2',2''-(10-(48-(6-Methyl-1,2,4,5-tetrazin-3-yl)-2,40,44-trioxo-6,9,12,15,18,21,24,27,30,33,36-undecaoxa-3,39,45-triazaoctatetracontyl)-1,4,7,10-tetraazacyclododecane-1,4,7-triyl)triacetic acid (S4)

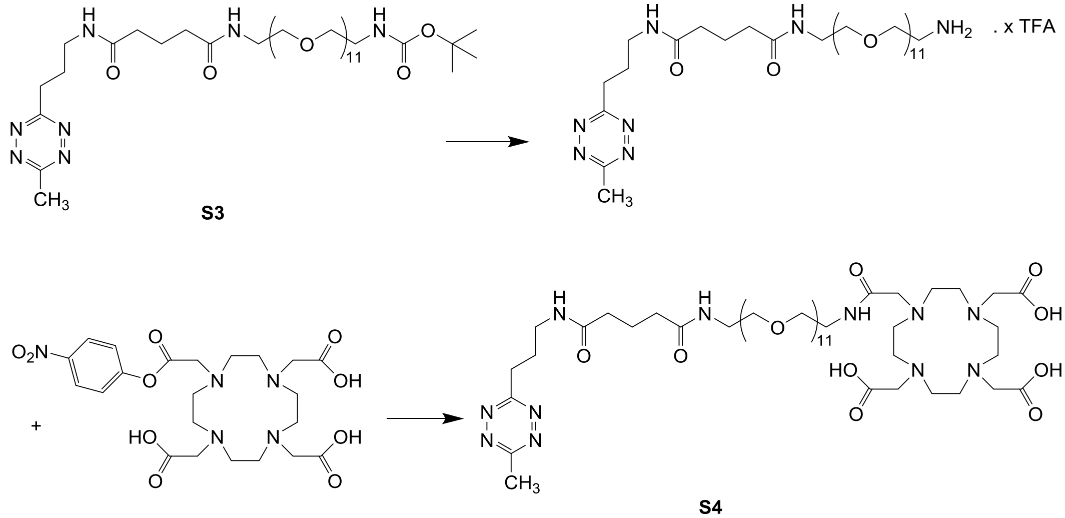

1,4,7,10-Tetraazacyclododecane-1,4,7,10-tetraacetic acid mono(4-nitrophenyl) ester was prepared according to Mier *et al.*[14]. Compound **S3** (2.03 g, 2.28 mmol) was dissolved in chloroform (25 mL), and trifluoroacetic acid (8 mL) was added. The pink solution was stirred at 20°C for 30 min, and subsequently concentrated and coevaporated with acetonitrile (2 times 15 mL). Then, the crude product was dissolved in acetonitrile (7 mL) and precipitated in cold diisopropyl ether (140 mL). The precipitate was collected by centrifugation and decantation, redissolved in acetonitrile (25 mL), and evaporated to dryness. HPLC-MS/PDA (5% to 100% in 10 min): t_r=3.80 min (*m/z*=+794.50 Da $[M+H]^+$; calcd 794.49 for $C_{35}H_{68}N_7O_{13}$; λ_{max}=278, 521 nm).

The intermediate was dissolved in DMF (20 mL) and DIPEA (2.32 g, 18.0 mmol) was added. Then, 1,4,7,10-tetraazacyclododecane-1,4,7,10-tetraacetic acid mono(4-nitrophenyl) ester (1.31 g, 2.50 mmol) was added, and the reaction mixture was stirred at 20°C in an inert atmosphere. After 1 h the pink solution was concentrated in vacuo, and the residue was dissolved in water and acidified with formic acid to pH=2. The product was purified by column chromatography (RP silica gel, acetonitrile / 0.1 v/v% aqueous formic acid = 15:85), and isolated by lyophilization, to yield product **S4** as a pink solid (1.94 g, 71%). ^1H NMR (D_2O): δ = 4.04 – 3.47 (m, 54H), 3.38 (m, 14H), 3.12 (m, 8H), 3.01 (s, 3H), 2.26 (m, 4H), 2.13 (m, 2H), 1.85 (m, 2H) ppm. HPLC-MS/PDA (5% to 100% in 10 min): t_r=3.82 min (*m/z*=+1180.83 Da $[M+H]^+$; calcd 1180.67 for $C_{51}H_{94}N_{11}O_{20}$; λ_{max}=276, 519 nm). See Supplementary Fig. 32-34.

Lutetium-(III) complex of 2,2',2''-(10-(48-(6-methyl-1,2,4,5-tetrazin-3-yl)-2,40,44-trioxo-6,9,12,15,18,21,24,27,30,33,36-undecaoxa-3,39,45-triazaoctatetracontyl)-1,4,7,10-tetraazacyclododecane-1,4,7-triyl)triacetic acid (3)

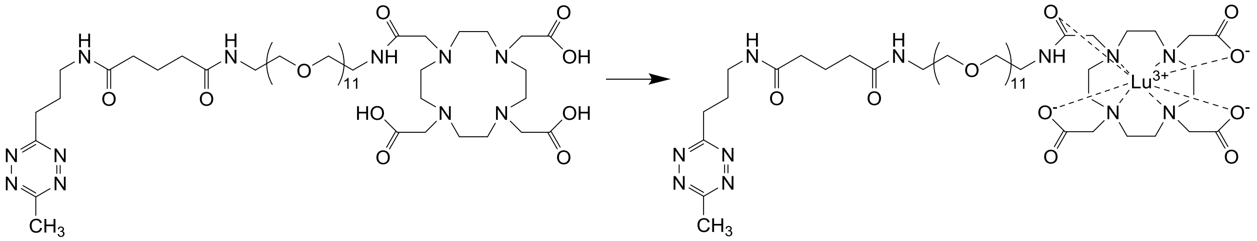

S4 **3**

To a solution of compound **S4** (1.94 g, 1.64 mmol) in 0.2 M aqueous sodium acetate buffer (60 mL, pH=5.5) was added lutetium(III) chloride hexahydrate (1.28 g, 3.28 mmol). The solution was stirred at 4°C for 16 h, and then the product was purified by column chromatography (RP silica gel, acetonitrile / 0.1 v/v% aqueous formic acid = 20:80), and isolated by lyophilization, to yield product **3** as a pink solid (1.70 g, 77%). ^{1}H NMR (D_2O): δ = 3.83 – 3.15 (m, 64H), 3.03 (s, 3H), 2.81 (m, 8H), 2.53 (m, 4H), 2.28 (m, 4H), 2.16 (m, 2H), 1.87 (m, 2H) ppm. ^{13}C NMR (D_2O): δ = 180.78, 175.99, 175.85, 175.74, 169.18, 167.52, 69.57, 69.37, 68.80, 68.55, 65.71, 55.81, 55.29, 39.67, 38.89, 38.35, 34.86, 34.82, 31.28, 26.61, 21.75, 20.05 ppm. HPLC-MS/PDA (5% to 100% in 10 min): t_r=3.62 min (*m/z* = +677.17 $[M+2H]^{2+}$, +1352.83 $[M+H]^{+}$, -1351.17 $[M-H]^{-}$, -1396.00 $[M+HCOO]^{-}$ Da; calcd 1352.58 for $C_{51}H_{91}N_{11}O_{20}Lu$ $[M+H]^{+}$). See Supplementary Fig. 35 and 36.

***tert*-Butyl ((2,40,44-trioxo-44-((6-(6-(pyridin-2-yl)-1,2,4,5-tetrazin-3-yl)pyridin-3-yl)amino)-6,9,12,15,18,21,24,27,30,33,36-undecaoxa-3,39-diazatetratetracontyl)oxy)carbamate (S6)**

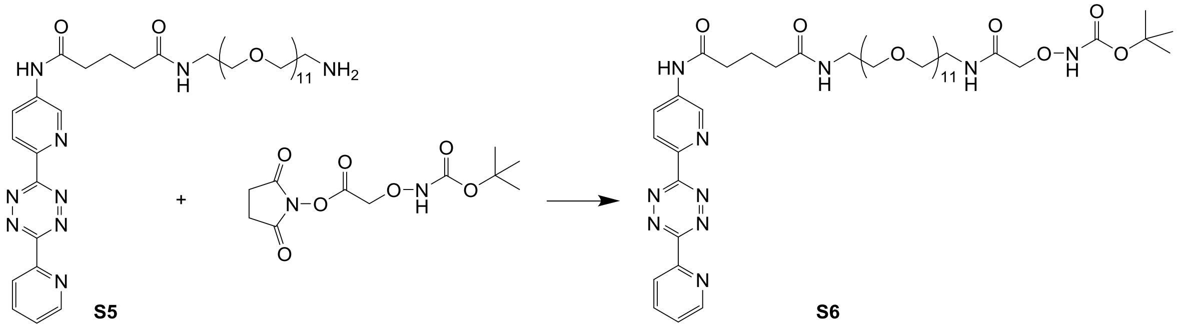

S5 **S6**

The synthesis of compound **S5** is described in Rossin *et al.*[4]. N-Boc-aminooxyacetic acid N-hydroxysuccinimide ester was prepared according to Aweda *et al.*[15]. To a solution of compound **S5** (169 mg, 0.189 mmol)) in acetonitrile (10 mL) was added 4-methylmorpholine (95 mg, 0.945 mmol) and N-Boc-aminooxyacetic acid N-hydroxysuccinimide ester (82 mg, 0.284 mmol). The pink solution was stirred at 20°C in an inert atmosphere for 1 h, and then concentrated in vacuo. The residue was redissolved in methylene chloride, and washed with 0.5 M citric acid (10 mL) and saturated sodium carbonate (2 times 10 mL). The organic layer was dried over sodium sulfate and evaporated to dryness. The crude product was purified by preparative RP-HPLC (35% acetonitrile / H_2O with 0.1 v/v% formic acid), and subsequently lyophilized to yield compound **S6** as a pink solid (128 mg, 65%). ^{1}H NMR ($CDCl_3$): δ =

9.67 (s, 1H), 8.98 (d, *J* = 6.3 Hz, 2H), 8.73 (dd, *J* = 8.4, 2.9 Hz, 2H), 8.64 (dd, *J* = 8.7, 2.3 Hz, 1H), 8.24 (s, 1H), 8.02 (t, *J* = 7.9 Hz, 1H), 7.89 (br s, 1H), 7.58 (dd, *J* = 7.5, 4.9 Hz, 1H), 6.72 (br s, 1H), 4.34 (s, 2H), 4.06 – 3.03 (m, 48H), 2.58 (t, *J* = 6.9 Hz, 2H), 2.38 (t, *J* = 6.9 Hz, 2H), 2.09 (t, *J* = 6.9 Hz, 2H), 1.47 (s, 9H) ppm. ^{13}C NMR ($CDCl_3$): δ = 173.10, 172.59, 169.06, 163.49, 163.36, 157.40, 150.90, 150.19, 143.84, 142.02, 138.62, 137.48, 126.47, 126.42, 125.14, 124.31, 82.39, 75.79, 70.53 (m), 70.50 (m), 70.47, 70.41, 70.33, 70.12, 69.60, 69.50, 39.34, 38.90, 35.98, 34.98, 28.16, 21.36 ppm. HPLC-MS/PDA (5% to 100% in 10 min): t_r=5.72 min (*m/z*=+1065.58 Da $[M+H]^+$; calcd 1065.55 for $C_{48}H_{77}N_{10}O_{17}$; λ_{max}=324, 529 nm). See Supplementary Fig. 37 and 38.

N1-(1-(Aminooxy)-2-oxo-6,9,12,15,18,21,24,27,30,33,36-undecaoxa-3-azaoctatriacontan-38-yl)-N5-(6-(6-(pyridin-2-yl)-1,2,4,5-tetrazin-3-yl)pyridin-3-yl)glutaramide (S7)

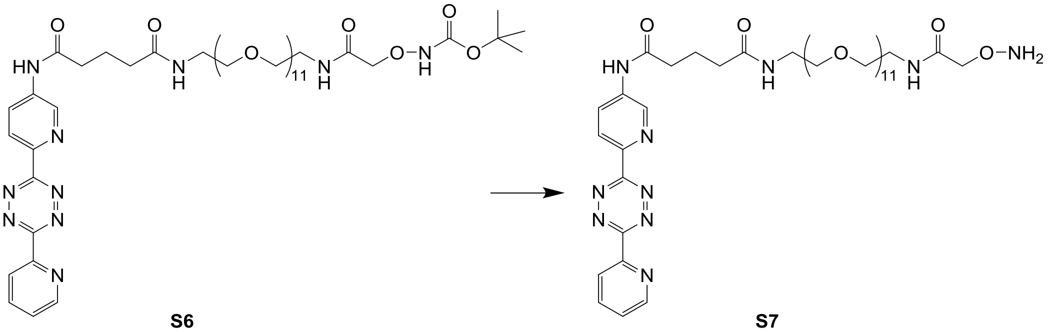

To a solution of compound **S6** (126 mg, 0.118 mmol) in methylene chloride (3.5 mL) was added trifluoroacetic acid (1 mL), and the pink solution was stirred at 20°C for 30 min. The mixture was concentrated in vacuo, redissolved in acetonitrile (2 mL), and precipitated in cold diethyl ether (20 mL). The product was collected by centrifugation and decantation, and dried in vacuo, to yield product **S7** as its TFA salt as a pink solid (76 mg, 60%). ^{1}H NMR (D_2O): δ = 8.78 (d, *J* = 4.5 Hz, 1H), 8.68 (d, *J* = 2.5 Hz, 1H), 8.54 (d, *J* = 7.9 Hz, 1H), 8.42 (d, *J* = 8.7 Hz, 1H), 8.19 – 8.05 (m, 2H), 7.73 (d, *J* = 2.2 Hz, 1H), 4.30 (s, 2H), 3.93 – 3.52 (m, 44H), 3.46 (t, *J* = 5.4 Hz, 2H), 3.41 (t, *J* = 5.3 Hz, 2H), 2.44 (t, *J* = 7.4 Hz, 2H), 2.34 (t, *J* = 7.4 Hz, 2H), 1.91 (m, 2H) ppm. HPLC-MS/PDA (5% to 100% in 10 min): t_r=5.05 min (*m/z*=+965.67 Da $[M+H]^+$; calcd 965.49 for $C_{43}H_{69}N_{10}O_{15}$; λ_{max}=324, 528 nm). See Supplementary Fig. 39 and 40.

N1-(1-(4-Fluorophenyl)-5-oxo-3,9,12,15,18,21,24,27,30,33,36,39-dodecaoxa-2,6-diazahentetracont-1-en-41-yl)-N5-(6-(6-(pyridin-2-yl)-1,2,4,5-tetrazin-3-yl)pyridin-3-yl)glutaramide (S8)

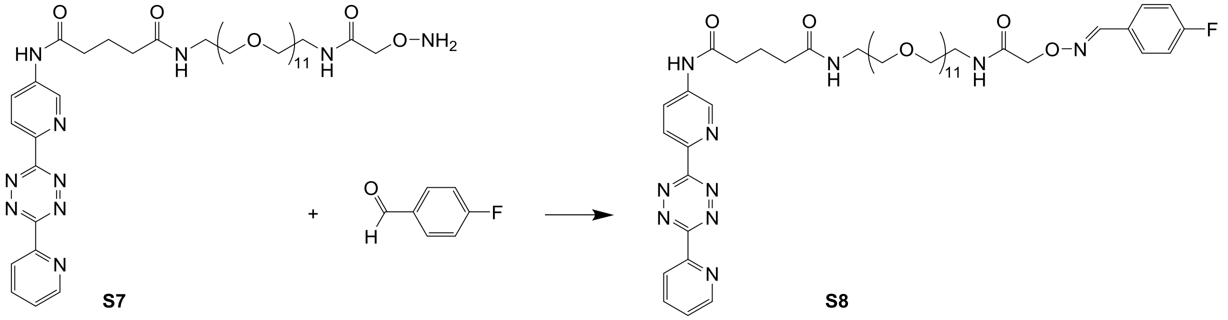

To a solution of compound **S7** (4.14 mg, 3.83 μmol) in methanol (2 mL) was added 0.1 M aqueous formic acid (0.2 mL) and 4-fluorobenzaldehyde (1.30 mg, 10.5 μmol). The mixture was heated at 60°C for 1 h and subsequently concentrated in vacuo. The crude product was purified by preparative RP-HPLC (41% acetonitrile / H_2O with 0.1 v/v% formic acid), and subsequently lyophilized to yield compound **S8** as a pink solid (3.6 mg, 88%). ^1H NMR ($CDCl_3$): δ = 9.66 (s, 1H), 8.99 (m, 2H), 8.74 (m, 2H), 8.64 (m, 1H), 8.18 (s, 1H), 8.01 (t, *J* = 7.8, 1H), 7.58 (m, 3H), 7.08 (t, *J* = 8.4 Hz, 2H), 6.72 (br m, 2H), 4.63 (s, 2H), 4.03 – 3.16 (m, 48H), 2.58 (t, *J* = 6.8 Hz, 2H), 2.38 (t, *J* = 6.8 Hz, 2H), 2.10 (m, 2H) ppm. ^{19}F NMR ($CDCl_3$): δ = -109.29 ppm. ^{13}C NMR ($CDCl_3$): δ = 172.97, 172.57, 169.48, 163.53, 163.39, 162.72, 150.96, 150.26, 149.61, 143.93, 142.05, 138.56, 137.41, 129.26, 129.18, 126.41, 125.12, 124.29, 116.09, 115.87, 109.98, 73.26, 70.51 (m), 70.12, 69.76, 69.64, 39.31, 38.78, 36.01, 34.99, 21.40 ppm. HPLC-MS/PDA (5% to 100% in 10 min): t_r=5.68 min (*m/z*=+1071.75 Da $[M+H]^+$; calcd 1071.52 for $C_{50}H_{72}FN_{10}O_{15}$; λ_{max}=324, 535 nm). See Supplementary Fig. 41-43.

10-(3-(*tert*-Butoxy)-3-oxopropyl)-2,2-dimethyl-4,8-dioxo-3,6-dioxa-5,9-diazaundecan-11-oic acid (S9)

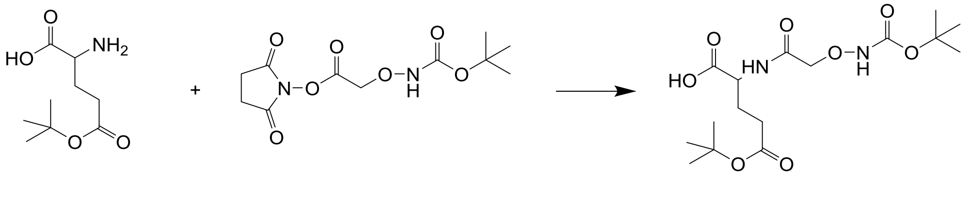

D-Glutamic acid 5-*tert*-butyl ester (123 mg, 0.603 mmol) was dissolved in a mixture of DMF (8 mL) and water (3 mL), 4-methylmorpholine (183 mg, 1.81 mmol) was added, followed by N-Boc-aminooxyacetic acid N-hydroxysuccinimide ester (158 mg, 0.548 mmol). The clear solution was stirred at 20°C for 1 h, and subsequently concentrated in vacuo, redissolved in DMF (1.5 mL), and precipitated in cold water (20 mL). The white precipitate was isolated by filtration, washed with water, suspended in acetonitrile (10 mL), evaporated to dryness, and dried in vacuo, to yield product **S9** as a white powder (180 mg, 87%). ^1H NMR (DMSO-d6): δ = 12.78 (s, 1H), 10.36 (s, 1H), 8.28 (d, *J* = 8.1 Hz, 1H), 4.30 (m, 1H), 4.20 (q, *J* = 17.3 Hz, 2H), 2.25 (m, 2H), 1.99 (m, 1H), 1.80 (m, 1H), 1.41 (s, 9H), 1.39 (s, 9H) ppm. HPLC-MS/PDA (5% to 100% in 10 min): t_r=5.94 min (*m/z*=+399.17 Da $[M+Na]^+$; calcd 399.17 for $C_{16}H_{28}N_2NaO_8$). See Supplementary Fig. 44 and 45.

tert-Butyl 4-(2-(((tert-butoxycarbonyl)amino)oxy)acetamido)-5,43,47-trioxo-47-((6-(6-(pyridin-2-yl)-1,2,4,5-tetrazin-3-yl)pyridin-3-yl)amino)-9,12,15,18,21,24,27,30,33,36,39-undecaoxa-6,42-diazaheptatetracontanoate (S10)

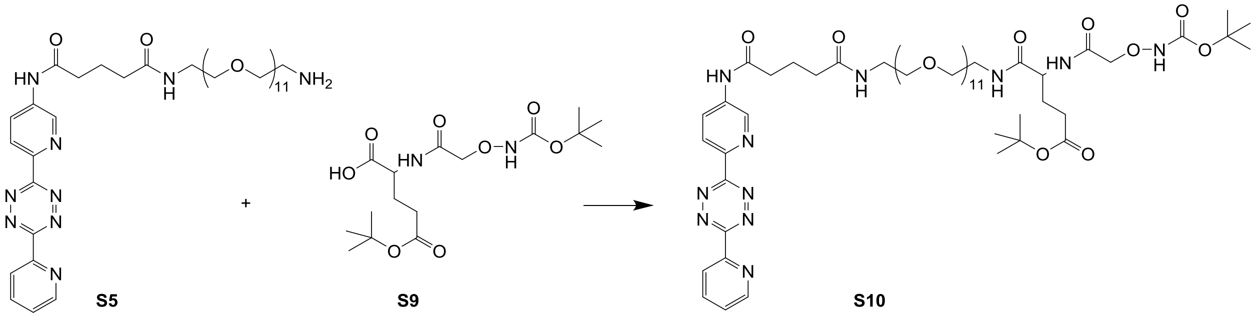

To a solution of compounds **S5** (182 mg, 0.205 mmol) and **S9** (77.0 mg, 0.205 mmol) in DMF (5 mL) was added 4-methylmorpholine (104 mg, 1.03 mmol) and PyBOP (107 mg, 0.205 mmol). The mixture was stirred at 20°C in an inert atmosphere for 30 min, and then concentrated in vacuo. The crude product was purified by preparative RP-HPLC (43% acetonitrile / H_2O with 0.1 v/v% formic acid), and subsequently lyophilized to yield compound **S10** as a pink solid (191 mg, 75%). ^{1}H NMR ($CDCl_3$): δ = 9.61 (s, 1H), 9.01 (d, J = 2.5 Hz, 1H), 9.98 (d, J = 4.6 Hz, 1H), 8.75 (s, 1H), 8.73 (s, 1H), 8.64 (dd, J = 8.7, 2.5 Hz, 1H), 8.26 (m, 2H), 8.01 (td, J = 7.8, 1.8 Hz, 1H), 7.58 (ddd, J = 7.7, 4.8, 1.2 Hz, 1H), 6.82 (t, J = 5.6 Hz, 1H), 6.56 (t, J = 5.6 Hz, 1H), 4.48 (td, J = 8.2, 5.2 Hz, 1H), 4.37 (d, J = 1.9 Hz, 2H), 3.89 – 3.27 (m, 48H), 2.58 (t, J = 6.8 Hz, 2H), 2.45 – 2.26 (m, 4H), 2.19 (m, 1H), 2.09 (m, 2H), 1.99 (m, H), 1.47 (s, 9H), 1.43 (s, 9H) ppm. ^{13}C NMR ($CDCl_3$): δ = 172.97, 172.57, 172.30, 170.89, 169.28, 163.51, 163.28, 157.67, 150.93, 150.20, 141.89, 138.65, 137.46, 126.62, 126.43, 125.17, 124.32, 82.84, 80.58, 75.84, 70.52 (m), 70.41, 70.33, 70.15, 69.64, 69.60, 52.49, 39.32, 35.94, 35.01, 31.79, 28.16, 28.07, 27.09, 21.33 ppm. HPLC-MS/PDA (5% to 100% in 10 min): t_r=6.37 min (*m/z*=+1250.42 Da $[M+H]^+$; calcd 1250.65 for $C_{57}H_{92}N_{11}O_{20}$, λ_{max}=325, 529 nm). See Supplementary Fig. 46 and 47.

4-(2-(Aminooxy)acetamido)-5,43,47-trioxo-47-((6-(6-(pyridin-2-yl)-1,2,4,5-tetrazin-3-yl)pyridin-3-yl)amino)-9,12,15,18,21,24,27,30,33,36,39-undecaoxa-6,42-diazaheptatetracontanoic acid (S11)

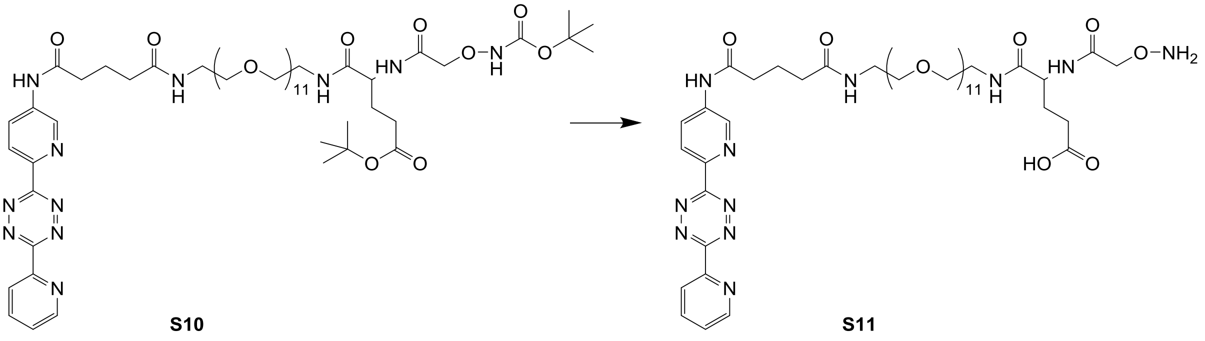

To a solution of compound **S10** (191 mg, 0.153 mmol) in chloroform (5 mL) was added trifluoroacetic acid (1 mL), and the pink mixture was stirred at 20°C in an inert atmosphere for 2 h, and then concentrated in vacuo. The residue was dissolved in acetonitrile (2 mL) and precipitated in diethyl ether (20 mL). The precipitate was collected by centrifugation and decantation, dried in vacuo, redissolved in

water (5 mL), and lyophilized, to yield the TFA salt of product **S11** as a pink solid (150 mg; 81%). ^{1}H NMR (DMSO-d6): δ = 10.56 (s, 1H), 9.06 (d, J = 2.5 Hz, 1H), 8.94 (d, J = 4.5 Hz, 1H), 8.61 (m, 2H), 8.43 (dd, J = 8.7, 2.5 Hz, 1H), 8.28 – 8.00 (m, 3H), 7.92 (t, J = 5.6 Hz, 1H), 7.73 (ddd, J = 7.6, 4.7, 1.2 Hz, 1H), 4.35 (m, 3H), 3.50 (m, 40H), 3.41 (m, 4H), 3.21 (m, 4H), 2.44 (t, J = 7.4 Hz, 2H), 2.28 – 2.11 (m, 4H), 1.98 – 1.64 (m, 4H) ppm. ^{13}C NMR (DMSO-d6): δ = 174.26, 172.53, 172.10, 171.12, 163.46, 163.18, 151.02, 150.60, 144.19, 141.67, 138.95, 138.24, 127.01, 126.52, 125.32, 124.61, 72.43, 70.19 (m), 69.99, 69.58, 69.29, 51.93, 39.01, 38.91, 36.09, 34.84, 30.44, 28.09, 21.35 ppm. HPLC-MS/PDA (5% to 100% in 10 min): t_r=5.02 min (*m/z*=+1094.42 Da $[M+H]^+$; calcd 1094.54 for $C_{48}H_{76}N_{11}O_{18}$, λ_{max}=324, 529 nm). See Supplementary Fig. 48 and 49.

4-(2-(((4-Fluorobenzylidene)amino)oxy)acetamido)-5,43,47-trioxo-47-((6-(6-(pyridin-2-yl)-1,2,4,5-tetrazin-3-yl)pyridin-3-yl)amino)-9,12,15,18,21,24,27,30,33,36,39-undecaoxa-6,42-diazaheptatetracontanoic acid (S12)

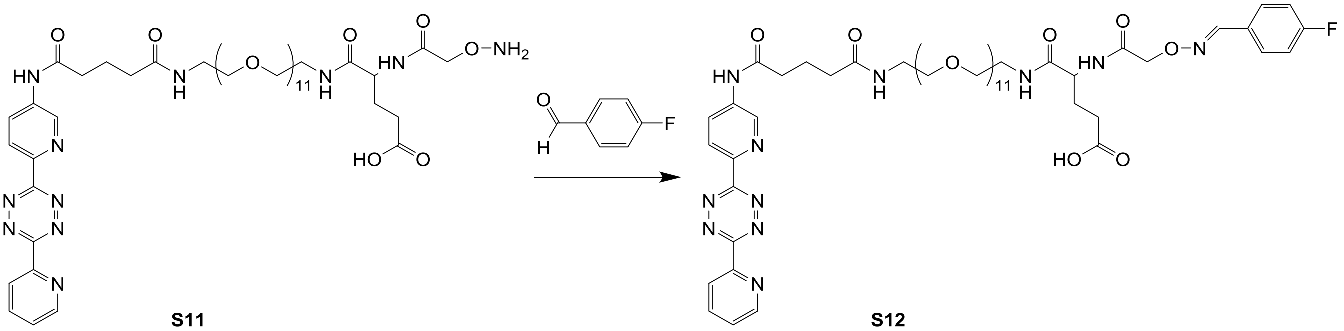

To a solution of compound **S11** (7.4 mg, 6.76 µmol) in methanol (2 mL) was added 0.1 M aqueous formic acid (0.2 mL) and 4-fluorobenzaldehyde (2.52 mg, 20.3 µmol). The mixture was heated at 60°C for 1 h and subsequently concentrated in vacuo. The crude product was purified by preparative RP-HPLC (37% acetonitrile / H_2O with 0.1 v/v% formic acid), and subsequently lyophilized to yield compound **S12** as a pink solid (2.5 mg, 31%). ^{1}H NMR (D_2O): δ = 8.88 (m, 2H), 8.71 (d, J = 8.1 Hz, 1H), 8.61 (d, J = 8.7 Hz, 1H), 8.36 – 8.23 (m, 2H), 8.22 (s, 1H), 7.85 (m, 1H), 7.52 (dd, J = 8.8, 5.5 Hz, 2H), 7.09 (t, J = 8.8 Hz, 2H), 4.62 (d, J = 1.6 Hz, 2H), 4.42 (dd, J = 9.3, 5.1 Hz, 1H), 3.65 (m, 48H), 3.55 (t, J = 5.3 Hz, 2H), 3.41 (t, J = 5.3 Hz, 2H), 3.36 (t, J = 5.3 Hz, 2H), 2.54 (t, J = 7.4 Hz, 2H), 2.39 (q, J = 7.2 Hz, 4H), 2.20 – 1.84 (m, 6H). ppm. ^{19}F NMR (D_2O): δ = -112.00 ppm. HPLC-MS/PDA (5% to 100% in 10 min): t_r=5.67 min (*m/z*=+1200.67 Da $[M+H]^+$; calcd 1200.56 for $C_{55}H_{79}FN_{11}O_{18}$, λ_{max}=324, 534 nm). See Supplementary Fig. 50 and 51.

***tert*-Butyl (6-((6-cyanopyridin-3-yl)amino)-6-oxohexyl)carbamate (S13)**

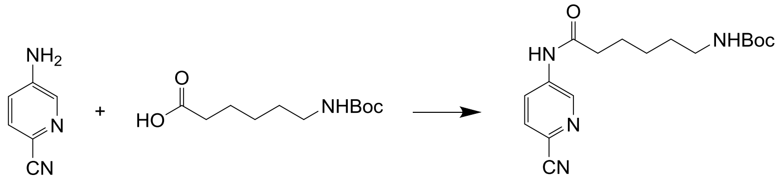

S13

5-Amino-2-cyanopyridine (1.07 g; 9.0 mmol), *N*-Boc-6-amino-hexanoic acid (1.08 g; 4.7 mmol), DMAP (0.9 g; 7.4 mmol) and pyridinium p-toluenesulfonate (PPTS; 0.37 g; 1.47 mmol) were suspended in chloroform (15 mL) and DCC (1.8 g; 8.7 mmol) in chloroform (2 mL) was added. The reaction mixture was stirred at room temperature for 18 h and subsequently concentrated to dryness. Acetonitrile (20 mL) was added and the precipitate was removed by filtration. The filtrate was evaporated to dryness, dissolved in chloroform (20 mL) and washed with 0.5 M citric acid (15 mL), 1 M $KHCO_3$ (15 mL) and water (15 mL). The organic phase was dried (Na_2SO_4), evaporated to dryness and the crude product was purified by column chromatography (silica, hexane / ethyl acetate 1:1) yielding product **S13** as a white solid (0.95 g; 61%). ^{1}H NMR ($CDCl_3$): δ = 9.10 (br, 1H), 8.70 (s, 1H), 8.45 (m, 1H), 7.70 (m, 1H), 4.80 (bs, 1H), 3.10 (m, 2H), 2.40 (t, 2H), 1.75 (m, 2H), 1.55 (m, 2H), 1.50-1.35 (multiple signals, 11H) ppm. ^{13}C NMR ($CDCl_3$): δ = 172.7, 156.4, 141.9, 138.5, 129.1, 127.1, 126.0, 117.4, 79.4, 40.1, 37.1, 29.6, 28.4, 26.1, 24.6 ppm. HPLC-MS/PDA: *m/z* = 355.3 $[M+Na]^+$ and 333.2 $[M+H]^+$; calcd 332.2 for $C_{17}H_{24}N_4O_3$).

***tert*-Butyl 6-oxo-6-(6-(6-(pyridin-2-yl)-1,2-dihydro-1,2,4,5-tetrazin-3-yl)pyridin-3-ylamino)hexyl carbamate (S14)**

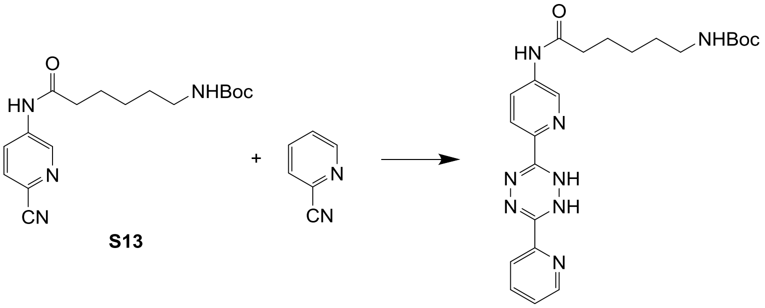

S13 **S14**

tert-Butyl 6-(6-cyanopyridin-3-ylamino)-6-oxohexylcarbamate (**S13**) (0.70 g; 2.1 mmol), 2-cyanopyridine (0.96 g; 9.2 mmol) and hydrazine hydrate (1.55 g; 31 mmol) were dissolved in ethanol (1.8 mL), and sulfur (240 mg; 7.5 mmol) was added. The mixture was stirred at 70°C under an Ar atmosphere for 2 h and subsequently at 50°C for 16 h. The orange suspension was diluted with chloroform (10 mL) and washed with water (2 times 15 mL) after which the organic phase was dried (Na_2SO_4) and evaporated to dryness. Finally, the crude product was purified by column chromatography (silica, chloroform / acetone 4:1) yielding product **S14** as an orange solid (0.65 g; 66%). ^{1}H NMR ($CDCl_3$ + 1 drop of DMSO-d6): δ = 8.65 (m, 1H), 8.60 (m, 1H), 8.55 (s, 1H), 8.50 (s, 1H), 8.20 (m, 1H), 8.05 (multiple signals, 2H), 7.90 (br, 1H), 7.75 (m, 1H), 7.35 (m, 1H), 4.65 (br, 1H), 3.10 (m, 2H), 2.40 (t, 2H), 1.75 (m, 2H), 1.55 (m, 2H), 1.50-1.35 (multiple signals, 11H) ppm. ^{13}C NMR ($CDCl_3$ + 1 drop of DMSO-d6): δ = 171.9, 155.5, 147.8,

146.8, 146.0, 145.8, 140.9, 138.8, 136.8, 136.2, 126.3, 124.3, 120.6, 120.5, 78.0, 39.9, 36.3, 29.1, 27.8, 25.7, 24.3 ppm. HPLC-MS/PDA: *m/z* = 467.4 $[M+H]^+$ (calcd 466.2 for $C_{23}H_{30}N_8O_3$). During HPLC-MS/PDA analysis some tetrazine is formed by oxidation, which is observed as a minor separate peak (*m/z* = 465.3 $[M+H]^+$).

tert-Butyl 6-oxo-6-(6-(6-(pyridin-2-yl)-1,2,4,5-tetrazin-3-yl)pyridin-3-ylamino) hexylcarbamate (S15)

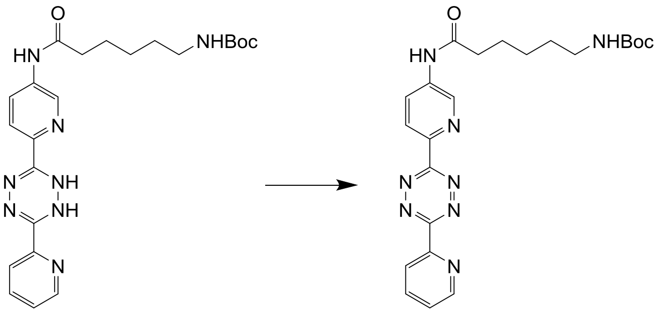

tert-Butyl 6-oxo-6-(6-(6-(pyridin-2-yl)-1,2-dihydro-1,2,4,5-tetrazin-3-yl)pyridin-3-ylamino) hexylcarbamate (**S14**) (0.30 g; 0.64 mmol) was dissolved in THF (1.5 mL) and acetic acid (2 mL). A solution of $NaNO_2$ (0.25 g; 3.62 mmol) in water (1 mL) was added dropwise and the solution turned red immediately. After brief stirring it was poured into 1 M $KHCO_3$ (50 mL), and the product was extracted with chloroform (50 mL). The organic layer was washed with water (50 mL), dried over Na_2SO_4, concentrated to dryness and the resulting material was suspended and stirred in hexane / chloroform 2:1 (25 mL). Filtration and drying of the residue yielded pure product **S15** (250 mg; 83%) as a purple solid. ^1H NMR ($CDCl_3$): δ = 9.10 (br, 1H), 8.95 (m, 1H), 8.85 (s, 1H), 8.75 (m, 3H), 8.00 (m, 1H), 7.55 (m, 1H), 4.70 (br, 1H), 3.15 (m, 2H), 2.50 (t, 2H), 1.80 (m, 2H), 1.50 (m, 2H), 1.50-1.35 (m, 11H) ppm. ^{13}C NMR ($CDCl_3$): δ = 172.6, 163.6, 163.3, 156.3, 151.0, 150.1, 144.1, 141.7, 138.5, 137.5, 126.9, 126.5, 125.3, 124.4, 79.4, 40.1, 37.3, 29.8, 28.4, 26.2, 24.8 ppm. HPLC-MS/PDA: *m/z* = 465.4 $[M+H]^+$; calcd 464.2 for $C_{23}H_{28}N_8O_3$).

6-Amino-*N*-(6-(6-(pyridin-2-yl)-1,2,4,5-tetrazin-3-yl)pyridin-3-yl)hexanamide (S16)

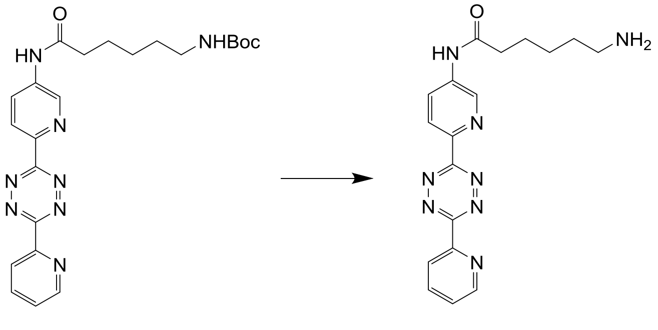

tert-Butyl 6-oxo-6-(6-(6-(pyridin-2-yl)-1,2,4,5-tetrazin-3-yl)pyridin-3-ylamino) hexylcarbamate **S15** (66 mg; 0.14 mmol) was dissolved in chloroform (6 mL) and TFA (6 mL) was added. The solution was stirred at room temperature for 2 h and subsequently evaporated to dryness. This yielded the product **S16** as its TFA salt as a reddish solid (68 mg; 100%) which was used immediately in the next step without further purification. HPLC-MS/PDA: *m/z* = 365.3 $[M+H]^+$; calcd 364.2 for $C_{18}H_{20}N_8O$).

***tert*-Butyl 2,2-dimethyl-4,8-dioxo-10-((6-oxo-6-((6-(6-(pyridin-2-yl)-1,2,4,5-tetrazin-3-yl)pyridin-3-yl)amino)hexyl)carbamoyl)-3,6-dioxa-5,9-diazatridecan-13-oate (S17)**

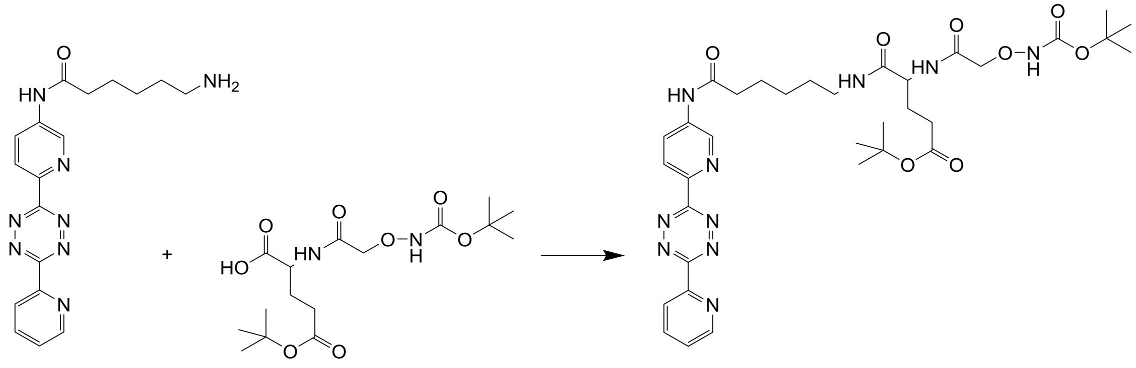

S16 S9 S17

To a solution of compounds **S16** (134 mg, 0.280 mmol) and **S9** (105 mg, 0.280 mmol) in DMF (4.5 mL) was added 4-methylmorpholine (141 mg, 1.40 mmol) and PyBOP (146 mg, 0.280 mmol). The mixture was stirred at 20°C in an inert atmosphere for 60 min, and then concentrated in vacuo. The crude product was purified by preparative RP-HPLC (45% acetonitrile / H_2O with 0.1 v/v% formic acid), and subsequently lyophilized to yield compound **S17** as a pink solid (144 mg; 71%). ^1H NMR ($CDCl_3$): δ = 9.26 (s, 1H), 8.97 (m, 2H), 8.84 – 8.63 (m, 3H), 8.38 (d, *J* = 7.8 Hz, 1H), 8.15 (s, 1H), 8.02 (td, *J* = 7.8, 1.8 Hz, 1H), 7.59 (m, 1H), 6.83 (t, *J* = 6.0 Hz, 1H), 4.48 (q, *J* = 5.3 Hz, 1H), 4.38 (d, *J* = 5.0 Hz, 2H), 3.28 (m, 2H), 2.45 (t, *J* = 7.5 Hz, 2H), 2.37 (m, 2H), 2.17 (m, 1H), 2.01 (m, 3H), 1.77 (m, 2H), 1.55 (m, 2H), 1.45 (s, 9H), 1.43 (s, 9H) ppm. ^{13}C NMR ($CDCl_3$): δ = 172.97, 172.55, 171.26, 169.60, 163.43, 162.97, 157.84, 150.77, 149.89, 141.40, 138.92, 137.74, 127.50, 126.63, 125.41, 124.44, 83.21, 80.97, 76.06, 52.84, 38.97, 37.29, 31.78, 28.58, 28.11, 28.06, 27.03, 25.93, 24.61 ppm. HPLC-MS/PDA (5% to 100% in 10 min): t_r=6.51 min (*m/z*=+723.17 Da $[M+H]^+$; calcd 723.36 for $C_{34}H_{47}N_{10}O_8$, λ_{max}=325, 539 nm). See Supplementary Fig. 52 and 53.

4-(2-(Aminooxy)acetamido)-5-oxo-5-((6-oxo-6-((6-(6-(pyridin-2-yl)-1,2,4,5-tetrazin-3-yl)pyridin-3-yl)amino)hexyl)amino)pentanoic acid (S18)

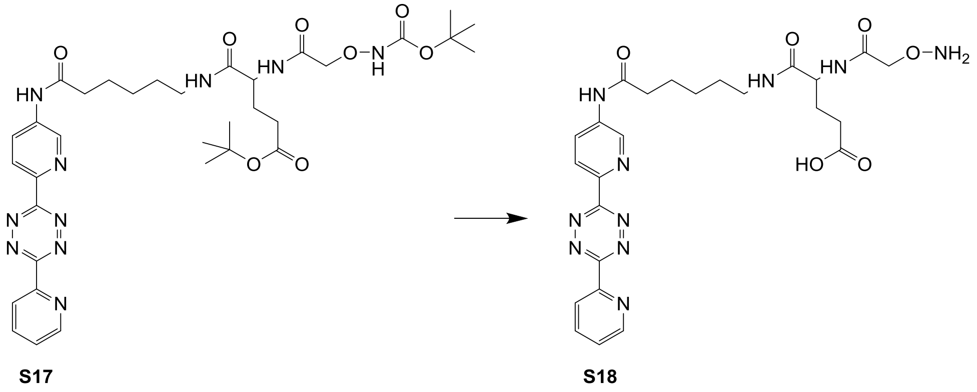

To a solution of compound **S17** (144 mg, 0.199 mmol) in chloroform (5 mL) was added trifluoroacetic acid (1 mL), and the pink mixture was stirred at 20°C in an inert atmosphere for 1 h, and then concentrated in vacuo. The residue was dissolved in acetonitrile (2 mL) and precipitated in diethyl ether (20 mL). The precipitate was collected by centrifugation and decantation, washed with diethyl ether (6 mL), and dried in vacuo, to yield the TFA salt of product **S18** as a pink solid (114 mg; 84%). ^{1}H NMR (DMSO-d6): δ = 10.55 (s, 1H), 9.05 (d, *J* = 2.5 Hz, 1H), 8.94 (d, *J* = 4.6 Hz, 1H), 8.61 (m, 2H), 8.43 (dd, *J* = 8.7, 2.5 Hz, 1H), 8.16 (dt, *J* = 7.8, 1.8 Hz, 1H), 8.11 (d, *J* = 8.2 Hz, 1H), 8.02 (t, *J* = 5.6 Hz, 1H), 7.73 (ddd, *J* = 7.7, 4.7, 1.2 Hz, 1H), 4.41 – 4.17 (m, 3H), 3.08 (m, 2H), 2.43 (t, *J* = 7.4 Hz, 2H), 2.23 (m, 2H), 1.93 (m, 1H), 1.78 (m, 1H), 1.65 (q, *J* = 7.5 Hz, 2H), 1.46 (q, *J* = 7.0 Hz, 2H), 1.33 (m, 2H) ppm. ^{13}C NMR (DMSO-d6): δ = 173.78, 172.35, 170.41, 168.05, 163.00, 162.72, 150.56, 150.15, 143.74, 141.22, 138.49, 137.76, 126.54, 126.07, 124.86, 124.14, 72.34, 51.55, 38.41, 36.28, 30.08, 28.78, 27.61, 25.97, 24.51 ppm. HPLC-MS/PDA (5% to 100% in 10 min): t_r=4.42 min (*m/z*=+567.25 Da [M+H]$^+$; calcd 567.24 for $C_{25}H_{31}N_{10}O_6$, λ_{max}=324, 530 nm). See Supplementary Fig. 54 and 55.

4-(2-(((4-Fluorobenzylidene)amino)oxy)acetamido)-5-oxo-5-((6-oxo-6-((6-(6-(pyridin-2-yl)-1,2,4,5-tetrazin-3-yl)pyridin-3-yl)amino)hexyl)amino)pentanoic acid (S19)

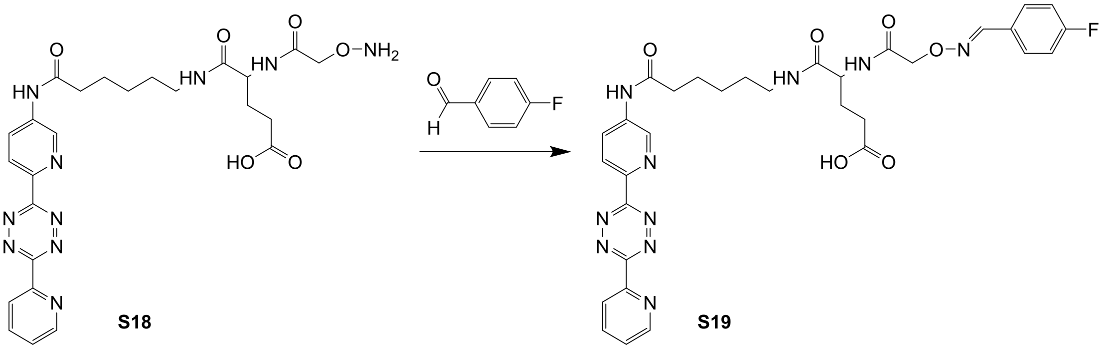

To a solution of compound **S18** (3.5 mg, 6.1 µmol) in methanol (2 mL) was added 0.1 M aqueous formic acid (0.2 mL) and 4-fluorobenzaldehyde (2.3 mg, 18.5 µmol). The mixture was heated at 60°C for 30 min and subsequently concentrated in vacuo. The crude product was purified by preparative RP-HPLC (34% acetonitrile / H_2O with 0.1 v/v% formic acid), and subsequently lyophilized to yield compound **S19** as a

pink solid (2.0 mg, 49%). ^{1}H NMR ($CDCl_3$/ MeOD-d4=5:1): δ = 8.94 (d, *J* = 4.7 Hz, 1H), 8.84 (d, *J* = 2.4 Hz, 1H), 8.80 – 8.71 (m, 2H), 8.68 (dd, *J* = 8.7, 2.5 Hz, 1H), 8.24 (s, 1H), 8.07 (td, *J* = 7.8, 1.8 Hz, 1H), 7.71 – 7.53 (m, 3H), 7.08 (t, *J* = 8.6 Hz, 2H), 4.63 (d, *J* = 3.4 Hz, 2H), 4.50 (dd, *J* = 8.2, 5.3 Hz, 1H), 3.22 (m, 2H), 2.45 (t, *J* = 7.5 Hz, 2H), 2.37 (m, 2H), 2.09 (m, 1H), 1.93 (m, 1H), 1.75 (q, *J* = 7.5 Hz, 2H), 1.55 (q, *J* = 7.1 Hz, 2H), 1.43 (m, 2H) ppm. ^{19}F NMR (MeOD-d4=5:1): δ = -105.63 ppm. HPLC-MS/PDA (5% to 100% in 10 min): t_r=5.52 min (*m/z*=+673.50 Da $[M+H]^+$; calcd 673.26 for $C_{32}H_{34}FN_{10}O_6$, λ_{max}=325, 525 nm). See Supplementary Fig. 56 and 57.

N6-(*tert*-Butoxycarbonyl)-N2-(2-(((*tert*-butoxycarbonyl)amino)oxy)acetyl)lysine (S20)

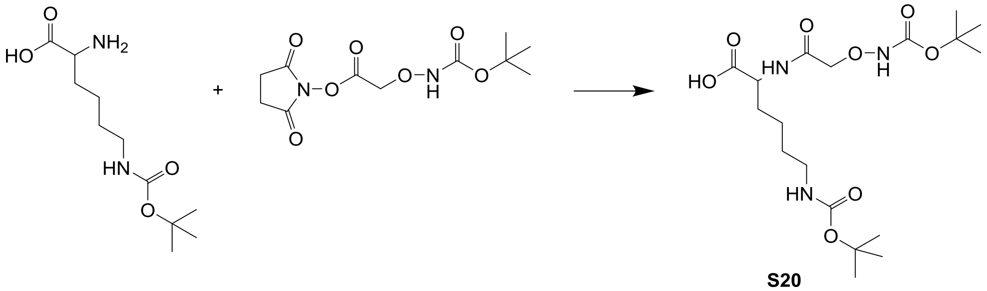

N-ε-Boc-*D*-lysine (90.7 mg, 0.368 mmol) was suspended in a mixture of DMF (2 mL) and water (2 mL). 4-Methylmorpholine (112 mg, 1.10 mmol) was added, followed by N-Boc-aminooxyacetic acid N-hydroxysuccinimide ester (106 mg, 0.368 mmol). The reaction mixture was stirred for 45 min in an inert atmosphere at 20°C, and then concentrated in vacuo, and coevaporated with DMF (2 times 3 mL). The crude product **S20** was used without further purification. HPLC-MS/PDA (5% to 100% in 10 min): t_r=5.28 min (*m/z*=+442.33 Da $[M+Na]^+$; calcd 442.22 for $C_{18}H_{33}N_3NaO_8$, λ_{max} < 200 nm). See Supplementary Fig. 58.

tert-Butyl ((4-(4-((tert-butoxycarbonyl)amino)butyl)-2,5,43,47-tetraoxo-47-((6-(6-(pyridin-2-yl)-1,2,4,5-tetrazin-3-yl)pyridin-3-yl)amino)-9,12,15,18,21,24,27,30,33,36,39-undecaoxa-3,6,42-triazaheptatetracontyl)oxy)carbamate (S21)

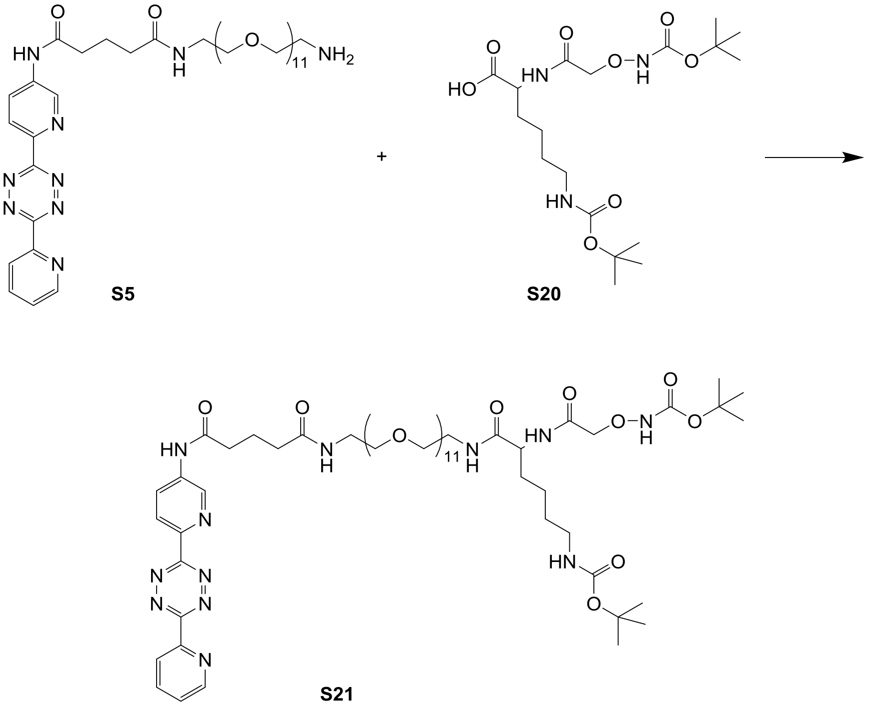

To a solution of compounds **S5** (173 mg, 0.193 mmol) and **S20** (81.0 mg, 0.193 mmol) in DMF (5 mL) was added 4-methylmorpholine (98 mg, 0.97 mmol) and PyBOP (100 mg, 0.193 mmol). The mixture was stirred at 20°C in an inert atmosphere for 60 min, and then concentrated in vacuo. The residue was dissolved in chloroform (5 mL) and washed with 0.5 M aqueous citric acid (2 times 3 mL), and 0.5 M aqueous sodium carbonate (3 mL), dried over sodium sulfate, and evaporated to dryness. The crude product was purified by preparative RP-HPLC (40% acetonitrile / H_2O with 0.1 v/v% formic acid), and subsequently lyophilized to yield compound **S21** as a pink solid (235 mg; 94%). ^{1}H NMR ($CDCl_3$): δ = 9.71 (s, 1H), 9.06 – 8.88 (m, 2H), 8.80 – 8.68 (m, 2H), 8.64 (dd, *J* = 8.7, 2.5 Hz, 1H), 8.42 (br s, 1H), 8.24 (br s, 1H), 8.01 (td, *J* = 7.8, 1.6 Hz, 1H), 7.58 (ddd, *J* = 7.5, 4.8, 1.2 Hz, 1H), 6.87 (t, *J* = 5.6 Hz, 1H), 6.72 (t, *J* = 5.7 Hz, 1H), 4.75 (br s, 1H), 4.43 (td, *J* = 8.1, 5.2 Hz, 1H), 4.38 (s, 2H), 3.94 – 3.23 (m, 48H), 3.09 (m, 2H), 2.58 (t, *J* = 6.9 Hz, 2H), 2.38 (t, *J* = 6.9 Hz, 2H), 2.09 (q, *J* = 7.0 Hz, 2H), 1.94 (m, 1H), 1.72 (m, 1H), 1.57 – 1.33 (m, 4H), 1.47 (s, 9H), 1.43 (s, 9H) ppm. ^{13}C NMR ($CDCl_3$): δ = 173.00, 172.58, 171.38, 169.29, 163.50, 163.37, 157.79, 155.98, 150.93, 150.22, 143.87, 142.05, 138.61, 137.43, 126.44, 125.12, 124.28, 82.79, 75.79, 70.49 (m), 70.30, 70.12, 69.67, 69.63, 53.10, 40.21, 39.29, 36.01, 35.02, 31.33, 29.54, 28.42, 28.15, 22.92, 21.37 ppm. HPLC-MS/PDA (5% to 100% in 10 min): t_r=6.13 min (*m/z*=+1293.75 Da $[M+H]^+$; calcd 1293.69 for $C_{59}H_{97}N_{12}O_{20}$, λ_{max} = 324, 526 nm). See Supplementary Fig. 59 and 60.

N1-(4-(4-Aminobutyl)-1-(aminooxy)-2,5-dioxo-9,12,15,18,21,24,27,30,33,36,39-undecaoxa-3,6-diazahentetracontan-41-yl)-N5-(6-(6-(pyridin-2-yl)-1,2,4,5-tetrazin-3-yl)pyridin-3-yl)glutaramide (S22)

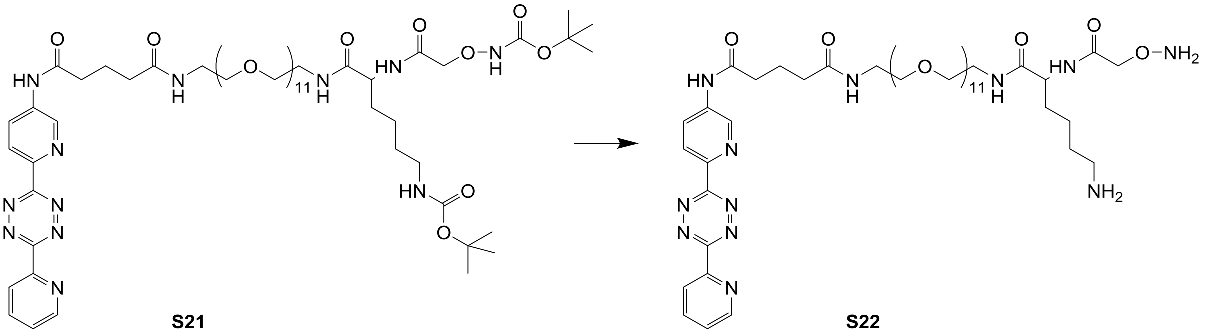

To a solution of compound **S21** (150 mg, 0.116 mmol) in chloroform (6 mL) was added trifluoroacetic acid (1 mL), and the pink mixture was stirred at 20°C in an inert atmosphere for 15 min, and then concentrated in vacuo. The crude product was purified by preparative RP-HPLC (20% acetonitrile / H_2O with 0.1 v/v% formic acid), and subsequently lyophilized to yield compound **S22** as a pink solid (77 mg; 61%). ^{1}H NMR (MeOD-d4): δ = 9.07 (d, *J* = 2.3 Hz, 1H), 8.88 (d, *J* = 4.8, Hz, 1H), 8.83 – 8.69 (m, 2H), 8.48 (dd, *J* = 8.8, 2.5 Hz, 1H), 8.18 (td, *J* = 7.8, 1.7 Hz, 1H), 7.73 (ddd, *J* = 7.7, 4.8, 1.2 Hz, 1H), 4.43 (dd, *J* = 7.8, 6.2 Hz, 1H), 4.21 (d, *J* = 2.1 Hz, 2H), 3.90 – 3.42 (m, 46H), 3.38 (t, *J* = 5.5 Hz, 2H), 2.97 (t, *J* = 7.7 Hz, 2H), 2.54 (t, *J* = 7.3 Hz, 2H), 2.34 (t, *J* = 7.4 Hz, 2H), 2.04 (q, *J* = 7.3 Hz, 2H), 1.88 (m, 1H), 1.80 – 1.61 (m, 3H), 1.47 (m, 2H) ppm. ^{13}C NMR (MeOD-d4): δ = 173.98, 172.89, 170.95, 163.26, 163.09, 150.09, 149.83, 143.89, 141.27, 138.96, 138.15, 126.94, 126.85, 124.78, 124.18, 73.22, 70.24, 69.94 (m), 69.59, 69.13, 68.89, 52.45, 39.32, 38.91, 35.45, 34.60, 31.32, 26.64, 22.17, 21.18 ppm. HPLC-MS/PDA (5% to 100% in 10 min): t_r=4.25 min (*m/z*=+1093.75 Da $[M+H]^+$; calcd 1093.59 for $C_{49}H_{81}N_{12}O_{16}$, λ_{max} = 324, 526 nm). See Supplementary Fig. 61 and 62.

2,2',2''-(10-(8-(2-(Aminooxy)acetamido)-2,9,47,51-tetraoxo-51-((6-(6-(pyridin-2-yl)-1,2,4,5-tetrazin-3-yl)pyridin-3-yl)amino)-13,16,19,22,25,28,31,34,37,40,43-undecaoxa-3,10,46-triazahenpentacontyl)-1,4,7,10-tetraazacyclododecane-1,4,7-triyl)triacetic acid (S23)

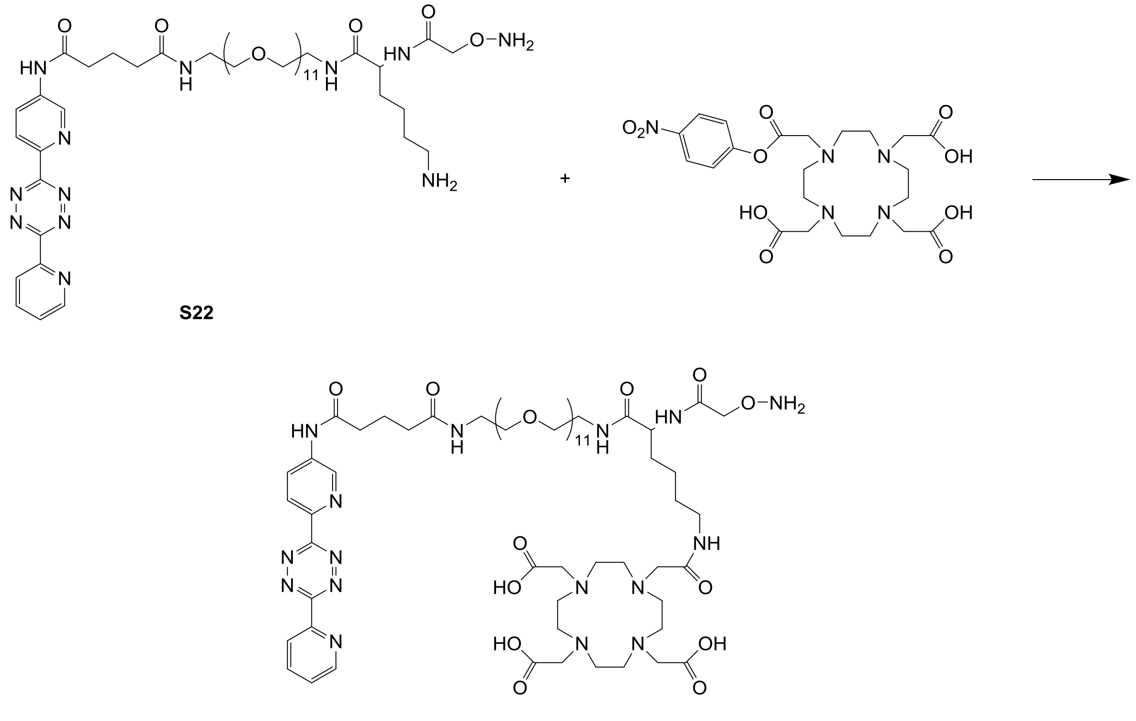

Compound **S22** (75 mg, 68.6 μmol) was dissolved in DMF (3 mL) and DIPEA (88.5 mg, 686 μmol) was added. The mixture was cooled to -10°C, and then 1,4,7,10-tetraazacyclododecane-1,4,7,10-tetraacetic acid, mono(4-nitrophenyl) ester (34.4 mg, 68.6 μmol) was added. After stirring at -10°C for 60 min, the pink solution was concentrated in vacuo, and the residue was dissolved in water and acidified with formic acid to pH=2. The crude product was purified by preparative RP-HPLC (21% acetonitrile / H_2O with 0.1 v/v% formic acid), and subsequently lyophilized to yield compound **S23** as a pink solid (40 mg; 39%). ^{1}H NMR (MeOD-d4): δ = 9.06 (d, J = 2.4 Hz, 1H), 8.88 (d, J = 4.8, Hz, 1H), 8.76 (m, 2H), 8.50 (dd, J = 8.7, 2.5 Hz, 1H), 8.17 (td, J = 7.8, 1.7 Hz, 1H), 7.73 (ddd, J = 7.7, 4.8, 1.2 Hz, 1H), 4.41 (dd, J = 9.4, 4.8 Hz, 1H), 4.19 (d, J = 2.8 Hz, 2H), 3.70 – 3.50 (m, 50H), 3.38 (m, 12H), 3.11 (m, 10H), 2.54 (t, J = 7.3 Hz, 2H), 2.34 (t, J = 7.3 Hz, 2H), 2.03 (t, J = 7.3 Hz, 2H), 1.83 (m, 1H), 1.72 m, 1H), 1.53 (m, 2H), 1.47 – 1.22 (m, 2H) ppm. ^{13}C NMR (MeOD-d4): δ = 173.96, 172.93, 171.42, 163.26, 150.10, 149.87, 143.85, 141.31, 138.97, 138.12, 126.92, 126.81, 124.80, 124.18, 73.62, 70.08 (m), 69.84, 69.13, 69.04, 55.83, 52.95, 50.87, 50.48, 38.96, 38.55, 35.47, 34.60, 31.21, 28.13, 22.72, 21.17 ppm. HPLC-MS/PDA (5% to 100% in 10 min): t_r=5.25 min (*m/z*=+1501.75 Da $[M+Na]^+$; calcd 1501.75 for $C_{65}H_{106}N_{16}NaO_{23}$, λ_{max} = 324, 529 nm). See Supplementary Fig. 63 and 64.

2,2',2''-(10-(8-(2-(((4-Fluorobenzylidene)amino)oxy)acetamido)-2,9,47,51-tetraoxo-51-((6-(6-(pyridin-2-yl)-1,2,4,5-tetrazin-3-yl)pyridin-3-yl)amino)-13,16,19,22,25,28,31,34,37,40,43-undecaoxa-3,10,46-triazahenpentacontyl)-1,4,7,10-tetraazacyclododecane-1,4,7-triyl)triacetic acid (S24)

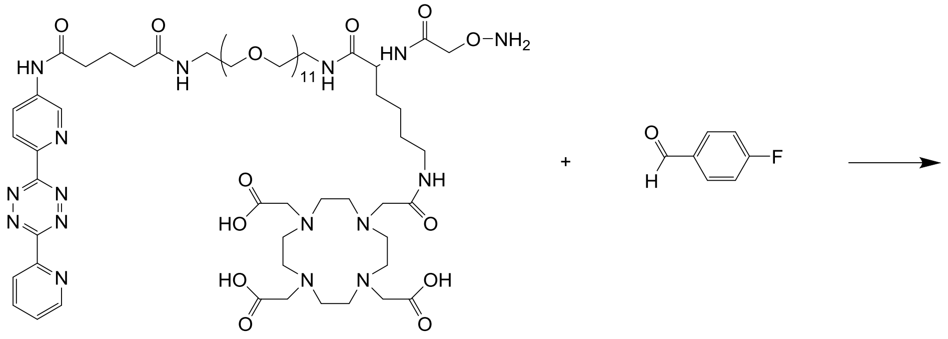

S23

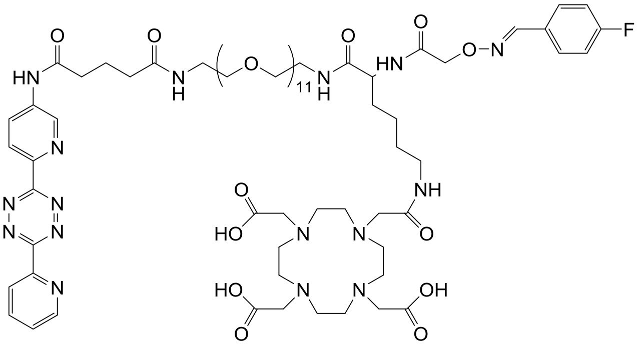

S24

To a solution of compound **S23** (5.2 mg, 3.5 μmol) in methanol (1.5 mL) was added 0.1 M aqueous formic acid (0.2 mL) and 4-fluorobenzaldehyde (1.3 mg, 10.4 μmol). The mixture was heated at 60°C for 30 min and subsequently concentrated in vacuo. The crude product was purified by preparative RP-HPLC (29% acetonitrile / H_2O with 0.1 v/v% formic acid), and subsequently lyophilized to yield compound **S24** as a pink solid (3.5 mg; 63%). ^{1}H NMR (MeOD-d4): δ = 9.05 (d, J = 2.5 Hz, 1H), 8.88 (d, J = 4.8, 1H), 8.81 – 8.68 (m, 2H), 8.50 (dd, J = 8.7, 2.5 Hz, 1H), 8.28 (s, 1H), 8.17 (td, J = 7.8, 1.8 Hz, 1H), 7.73 (ddd, J = 7.7, 4.8, 1.2 Hz, 1H), 7.69 – 7.57 (m, 2H), 7.14 (t, J = 8.7 Hz, 2H), 4.64 (s, 2H), 4.43 (dd, J = 9.0, 5.2 Hz, 1H), 3.80 – 3.34 (m, 68H), 3.22 – 2.85 (m, 10H), 2.54 (t, J = 7.3 Hz, 2H), 2.34 (t, J = 7.4 Hz, 2H), 2.03 (q, J = 7.3 Hz, 2H), 1.88 – 1.75 (m, 1H), 1.69 (m, 1H), 1.49 (m, 2H), 1.44 – 1.18 (m, 2H). ppm. ^{19}F NMR (MeOD-d4): δ = -111.97 ppm. ^{13}C NMR (MeOD-d4): δ = 175.89, 175.33, 174.85, 174.72, 172.56, 172.40, 170.38, 165.18, 165.01, 151.79, 140.90, 130.95, 126.84, 126.60, 72.23, 71.99, 71.76, 58.00, 40.87, 23.09. ppm. HPLC-MS/PDA (5% to 100% in 10 min): t_r=5.32 min (m/z=+1585.75 Da $[M+H]^+$; calcd 1585.79 for $C_{72}H_{110}FN_{16}O_{23}$, λ_{max} = 324, 531 nm). See Supplementary Fig. 65-67.

(E)-6-((76-Amino-75-oxo-2,5,8,11,14,17,20,23,26,29,32,35,38,41,44,47,50,53,56,59,62,65,68,71-tetracosaoxa-74-azaoctacontan-80-yl)carbamoyl)-6-methylcyclooct-2-en-1-yl ((S)-1-(((S)-1-(((3R,4S,5S)-1-((S)-2-((1R,2R)-3-(((1S,2R)-1-hydroxy-1-phenylpropan-2-yl)amino)-1-methoxy-2-methyl-3-oxopropyl)pyrrolidin-1-yl)-3-methoxy-5-methyl-1-oxoheptan-4-yl)(methyl)amino)-3-methyl-1-oxobutan-2-yl)amino)-3-methyl-1-oxobutan-2-yl)(methyl)carbamate (S27)

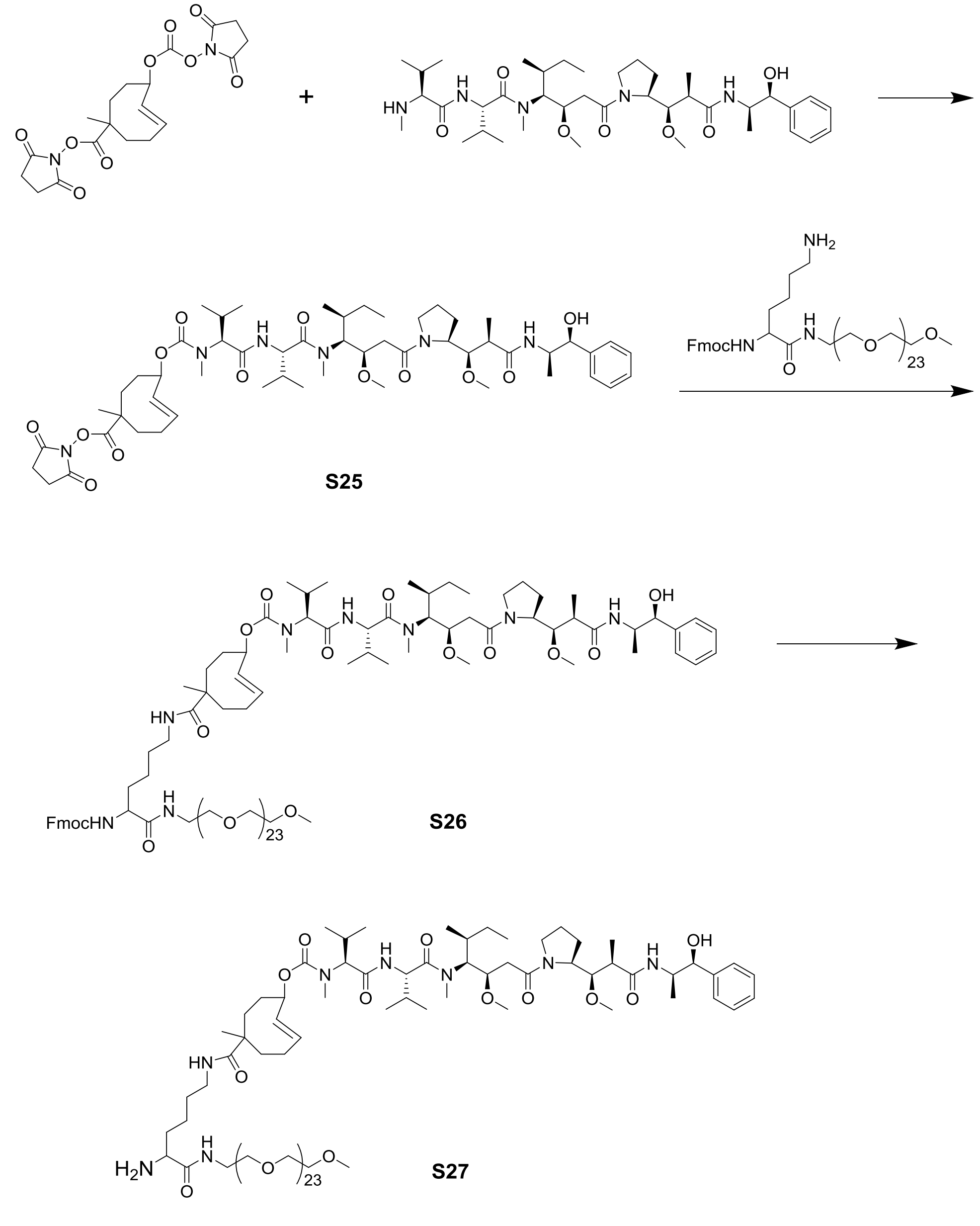

Bis-NHS-TCO was synthesized as reported in Rossin *et al.*[6]. Compound **S27** was prepared in several steps in situ. To a solution of monomethyl auristatin E (MMAE; 112 mg as mono TFA salt, 135 µmol, Levena Biopharma) in 2 mL of anhydrous DMF in a glass vial was added bis-NHS-TCO (57 mg, 135 µmol) and DIEA (70 µL, 405 µmol). The mixture was stirred at room temperature in the dark for 3 days, at which point HPLC-MS analysis indicated >94% conversion to intermediate **S25**. To this reaction mixture was added Fmoc-Lys-PEG$_{24}$ (as mono TFA salt, 251 mg, 162 µmol, Levena Biopharma) and DIEA (28 µL, 161 µmol). The reaction mixture was stirred at room temperature in the dark for 3 days, at which point

HPLC-MS analysis showed formation of compound **S26** and that all **S25** was consumed. To this mixture was added piperidine (150 µL, 1.52 mmol) and the stirring was continued for 20 min, at which point HPLC-MS analysis indicated complete formation of compound **S27**. The product was purified by preparative HPLC (20 min run, 10 to 70% acetonitrile at 50 mL/min). The collected fractions were analysed by HPLC-MS. The pure fractions were lyophilized in the dark to give **S27** as acetate salt as a viscous colourless gum (275 mg; 93%). ^{1}H NMR ($CDCl_3$): δ = 7.97 (br s, 1H), 7.36 (m, 2H), 7.31 (m, 2H), 7.24 (m, 1H), 6.53-6.59 (br m, 1H), 5.77-5.91 (br m, 1H), 5.57-5.62 (br m, 1H), 4.66-4.72 (br m, 1H), 4.25 (br m, 1H), 4.13 (br m, 1H), 3.9-4.1 (br m, 10H), 3.85 (m, 1H), 3.77 (m, 1H), 3.63 (br s, 92H), 3.48-3.54 (m, 7H), 3.38-3.41 (m, 6H), 3.36 (s, 3H), 3.29-3.32 (m, 3H), 3.15-3.22 (m, 2H), 2.97-3.03 (m, 2H), 2.94 (m, 2H), 2.89 (m, 1H), 2.43 (m, 1H), 2.35 (m, 1H), 2.17-2.30 (br m, 3H), 1.95-2.10 (m, 8H), 1.73-1.87 (br m, 6H), 1.55-1.67 (br m, 2H), 1.45-1.53 (m, 2H), 1.30-1.41 (br m, 3H), 1.23 (d, J = 6.5 Hz, 3H), 1.11 (s, 3H), 0.95-1.05 (m, 7H), 0.78-0.94 (m, 15H) ppm. ^{13}C NMR ($CDCl_3$): δ = 180.62, 174.82, 173.73, 170.79, 170.17, 161.81, 161.54, 156.62, 141.44, 131.77, 131.41, 128.45, 128.22, 127.43, 126.54, 82.20, 78.75, 75.97, 73.33, 72.11, 71.21, 70.69, 70.59 (br), 70.45, 70.31, 70.04, 65.20, 64.99, 61.11, 60.28, 59.18, 58.51, 58.16, 54.75, 54.22, 51.74, 51.36, 48.00, 46.86, 45.77, 45.17, 44.88, 44.61, 44.44, 39.32, 39.24, 37.92, 36.12, 33.71, 33.64, 31.89, 31.63, 31.52, 31.35, 31.20, 29.73, 29.23, 26.29, 25.93, 25.17, 25.09, 23.58, 22.87, 21.61, 19.56, 19.48, 18.77, 18.16, 17.98, 16.17, 14.58, 14.16, 11.10 ppm. HPLC-MS: *m/z* 1064.4 Da $[M+2H]^{2+}$; calcd 1063.68 for $C_{105}H_{194}N_8O_{35}$. See Supplementary Fig. 68-70.

Maleimide-PEG$_{24}$-TCO-MMAE (1)

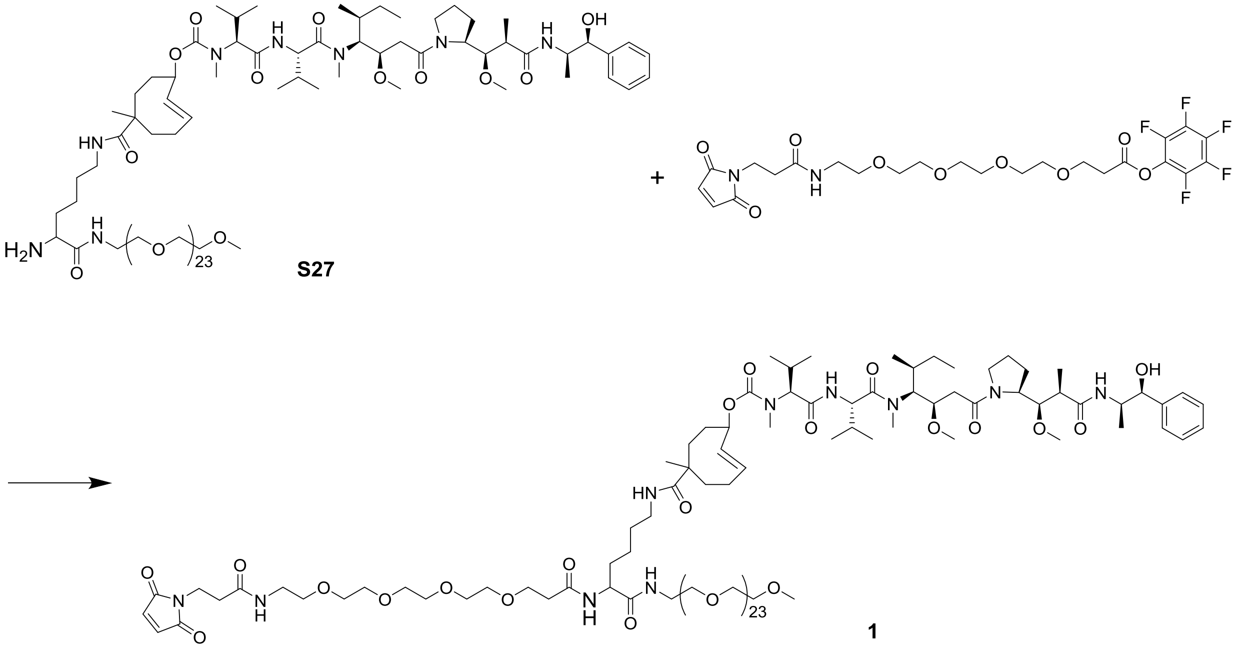

To a solution of **S27** (mono acetate salt, 275 mg, 126 µmol) in 3 mL of anhydrous DMF in a glass vial was added Mal-NH-PEG$_4$-$CH_2CH_2COOPFP$ (75 mg, 129 µmol, Levena Biopharma) and DIEA (44 µL, 253 µmol). The mixture was stirred at room temperature for 10 min in the dark, at which point HPLC-MS analysis indicated complete conversion to compound **1**. The reaction was purified by preparative HPLC

(20 min run, from 10 to 80% acetonitrile at 50 mL min^{-1}). The collected fractions were analysed by HPLC-MS and the pure fractions were lyophilized in a dark to give **S29** as viscous gum (181 mg; 57%). ^{1}H NMR ($CDCl_3$): δ = 7.36 (m, 2H), 7.31 (m, 2H), 7.23 (m, 1H), 6.85-7.16 (br m, 2H), 6.67-6.73 (br m, 2H), 6.45-6.65 (br m, 1H), 5.55-5.90 (m, 3H), 4.55-5.25 (m, 4H), 4.35 (m, 1H), 4.23 (m, 1H), 4.00-4.18 (br m, 3H), 3.81 (m, 3H), 3.67-3.77 (br m, 3H), 3.55-3.67 (br m, 104H), 3.45-3.55 (m, 8H), 3.34-3.43 (m, 11H), 3.28-3.31 (m, 3H), 3.07-3.20 (br m, 3H), 2.85-3.05 (m, 5H), 2.58 (br m, 1H), 2.49 (m, 5H), 2.42 (m, 1H), 2.35 (m, 1H), 2.23 (m, 4H), 1.90-2.10 (br m, 6H), 1.82 (m, 6H), 1.59 (m, 2H), 1.47 (m, 2H), 1.31 (m, 2H), 1.22 (d, J = 7.0 Hz, 3H), 1.09 (m, 3H), 0.93-1.04 (m, 7H), 0.78-0.93 (m, 15H) ppm. ^{13}C NMR ($CDCl_3$): δ = 180.51, 174.70, 173.84, 171.96, 171,67, 170.71, 170.09, 156.56, 141.50, 134.41, 131.43, 128.20, 127.40, 126.50, 82.19, 75.98, 73.26, 72.13, 70.75 (br.), 70.62, 70.53, 70.49, 70.43, 70.32, 69.96, 69.83, 67.49, 65.50, 65.03, 61.05, 60.25, 59.18, 58.14, 54.11, 53.12, 51.77, 47.94, 45.86, 45.07, 44.42, 39.47, 39.43, 37.95, 37.09, 36.13, 34.68, 34.56, 33.66, 32.00, 31.54, 31.29, 31.17, 29.71, 29.25, 26.27, 25.93, 25.16, 25.10, 22.97, 19.86, 19.55, 18.75, 18.19, 16.17, 14.53, 14.08, 11.11 ppm. HPLC-MS: *m/z* 1263.0 Da $[M+2H]^{2+}$; calcd 1262.77 for $C_{123}H_{220}N_{10}O_{43}$. See Supplementary Fig. 71-73.

Maleimide-PEG$_{24}$-Val-Cit-PABC-MMAE (2)

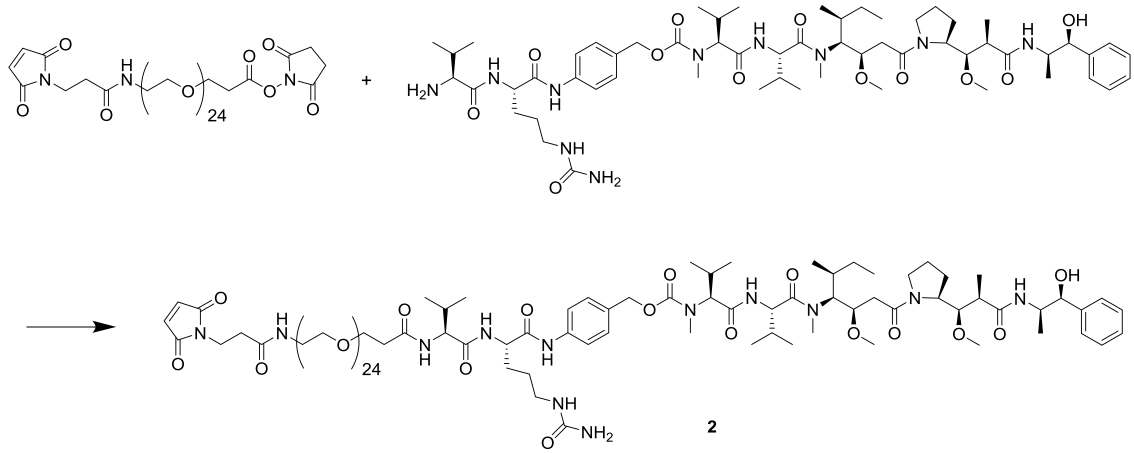

To a stirred solution of Mal-PEG$_{24}$-NHS ester (70 mg, 50 µmol, Quanta Biodesign) and Val-Cit-PABC-MMAE (45 mg, 40 µmol, Levena Biopharma)[16] in anhydrous DMF (1 mL) was added DIEA (9 µL). The reaction mixture was stirred at room temperature for 1 h and purified directly by preparative HPLC (20 min run, from 20 to 70% acetonitrile at 50 mL min^{-1}) to give the desired product **2** as a colourless syrup after lyophilization (70 mg; 73%). ^{1}H NMR ($CDCl_3$): δ = 8.94 (s, 1H), 7.68 (d, J = 8.5 Hz, 2H), 7.36 (m, 2H), 7.32 (m, 2H), 7.20-7.30 (m, 3H), 6.51-6.65 (m, 4H), 4.91-5.18 (br m, 3H), 4.68 (m, 3H), 4.26 (m, 2H), 4.11 (m, 2H), 3.95-4.07 (br m, 2H), 3.83 (m, 4H), 3.77 (m, 2H), 3.64 (br m, 100H), 3.54 (m, 9H), 3.40 (m, 6H), 3.31 (s, 3H), 3.23 (m, 2H), 3.10 (m, 1H), 3.00 (m, 2H), 2.85-2.95 (m, 3H), 2.52-2.68 (br m, 6H), 2.51 (m, 6H), 2.45 (m, 6H), 2.38 (m, 6H), 2.25 (m, 6H), 2.04 (m, 4H), 1.95 (m, 1H), 1.84 (m, 2H), 1.73 (m, 2H), 1.59 (m, 2H), 1.38 (m, 1H), 1.24 (m, 3H), 0.95-1.05 (m, 15H), 0.75-0.90 (m, 15H), 0.71 (m, 1H) ppm. ^{13}C NMR ($CDCl_3$): δ = 174.71, 173.03, 172.89, 172.09, 170.63, 170.38, 170.01, 160.86, 157.48, 141.46, 138.35, 134.36, 132.25, 129.19, 128.65, 128.42, 128.19, 127.41, 126.47, 120.10, 82.17, 78.23, 75.91, 70.68 (br.), 70.54, 70.33, 69.83, 67.49, 67.35, 65.19, 61.04, 60.16, 59.71,58.08, 54.09, 53.48, 51.64, 47.93, 45.03, 39.42, 39.20, 37.97, 36.97, 34.69, 34.51, 33.54, 31.13, 30.36, 29.73, 28.98, 26.93, 26.25,

25.91, 25.12, 25.05, 19.53, 19.43, 18.76, 18.25, 17.75, 16.15, 14.49, 14.13, 11.06 ppm. HPLC-MS: *m/z* 1202.0 $[M+2H]^{2+}$; calcd 1201.71 for $C_{116}H_{202}N_{12}O_{40}$. See Supplementary Fig. 74-76.

Supplementary References

1. van Duijnhoven, S. M. J., *et al.* Diabody Pretargeting with Click Chemistry In Vivo. *J. Nucl. Med.* **56**, 1422-1428 (2015).

2. Ngai, W. M. & Reilly, R. M. A simple method to determine the immunoreactivity of radiolabelled monoclonal antibodies to the TAG-72 antigen. *Appl. Radiat. Isot.* **44**, 1193-1197 (1993).

3. Lindmo, T., Boven, E., Cuttitta, F., Fedorko, J. and Bunn Jr, P. A. Determination of the immunoreactive fraction of radiolabeled monoclonal antibodies by linear extrapolation to binding at infinite antigen excess. *J. Immunol. Meth.* **72**, 77-89 (1984).

4. Rossin, R., *et al.* In Vivo Chemistry for Pretargeted Tumor Imaging in Live Mice. *Angew. Chem. Int. Ed.* **49**, 3375-3378 (2010).

5. Rossin, R., *et al.* Highly Reactive trans-Cyclooctene Tags with Improved Stability for Diels-Alder Chemistry in Living Systems. *Bioconjug. Chem.* **24**, 1210-1217 (2013).

6. Rossin, R., *et al.* Triggered Drug Release from an Antibody–Drug Conjugate Using Fast "Click-to-Release" Chemistry in Mice. *Bioconjug. Chem.* **27**, 1697-1706 (2016).

7. Heskamp, S., *et al.* Alpha- versus beta-emitting radionuclides for pretargeted radioimmunotherapy of CEA-expressing human colon cancer xenografts. *J. Nucl. Med.* **58**, 926-933 (2017).

8. Li, L., *et al.* Site-Specific Conjugation of Monodispersed DOTA-PEGn to a Thiolated Diabody Reveals the Effect of Increasing PEG Size on Kidney Clearance and Tumor Uptake with Improved 64-Copper PET Imaging. *Bioconjug. Chem.* **22**, 709-716 (2011).

9. Patent US20110268656A1

10. Rossin, R., van Duijnhoven, S. M. J., Läppchen, T., van den Bosch, S. M. & Robillard, M. S. Trans-Cyclooctene Tag with Improved Properties for Tumor Pretargeting with the Diels–Alder Reaction. *Mol. Pharm.* **11**, 3090-3096 (2014).

11. Rossin, R., Läppchen, T., van den Bosch, S. M., Laforest, R. & Robillard, M. S. Diels-Alder Reaction for Tumor Pretargeting: In Vivo Chemistry Can Boost Tumor Radiation Dose Compared with Directly Labeled Antibody. *J. Nucl. Med.* **54**, 1989-1995 (2013).

12. Burke, P. J., *et al.* Optimization of a PEGylated Glucuronide-Monomethylauristatin E Linker for Antibody-Drug Conjugated. *Mol. Cancer Ther.* **16**, 116-123 (2017).

13. Houssin, R., Bernier, J. -L. & Hénichart, J. -P. A Convenient and General Method for the Preparation of tert-Butoxycarbonylaminoalkanenitriles and Their Conversion to Mono-tert-butoxycarbonylalkanediamines. *Synthesis* **1988**, 259-261 (1988).

14. Mier, W., *et al.* Conjugation of DOTA Using Isolated Phenolic Active Esters: The Labeling and Biodistribution of Albumin as Blood Pool Marker. *Bioconjug. Chem.* **16**, 237-240 (2005).

15. Aweda, T. A., *et al.* New Covalent Capture Probes for Imaging and Therapy, Based on a Combination of Binding Affinity and Disulfide Bond Formation. *Bioconjug. Chem.* **22**, 1479-1483 (2011).

16. Synthesized according to published procedures, WO/2004/010957.
